# Light and Alternating Temperatures Release Seed Dormancy in the Invasive *Dipsacus fullonum* L. Through ROS Homeostasis and ABA Regulation

**DOI:** 10.1111/ppl.70642

**Published:** 2025-11-19

**Authors:** Paola Frazzetto, Héctor R. Huarte, Giuseppe D. Puglia, Andrey Prjibelski, Shashank Sagar Saini, Valentina Giglio, Sandro Dattilo, Antonia Cristaudo

**Affiliations:** ^1^ Department of Biological, Geological and Environmental Sciences University of Catania Catania Italy; ^2^ Universidad Nacional de Lomas de Zamora/CONICET. Camino de Cintura y Juan XXIII Buenos Aires Argentina; ^3^ Institute for Agricultural and Forestry Systems in the Mediterranean (ISAFoM), National Research Council of Italy (CNR) Catania Italy; ^4^ Department of Computer Science University of Helsinki Helsinki Finland; ^5^ Department of Biomedical and Dental Sciences and Morphofunctional Imaging University of Messina Messina Italy; ^6^ Institute for Polymers, Composites, and Biomaterials CNR‐IPCB Via Paolo Gaifami 18 Catania Italy

**Keywords:** common teasel, environmental cues, histone deacetylase, long‐reads RNA‐seq, PIF1, ROS scavengers, seed germination, weeds

## Abstract

Seeds have developed mechanisms to perceive environmental signals, such as light and temperature, which govern germination and enhance the chance of seedling establishment. This study examined the foundations of light and temperature sensitivity in the seed dormancy release of a common weed, *Dipsacus fullonum* . By screening six accessions from two different regions, we identified two unique germination behaviors: one sensitive to environmental stimuli and one neutral to them. In the sensitive accession, ABA is crucial for regulating dormancy release, as it accumulates in seeds subjected to darkness, while it decreases under other conditions. We observed a rise of reactive oxygen species (ROS) under conditions that stimulate germination and highlighted that their presence enhanced germination even in the absence of light. This study employed a long‐read RNA‐seq technology to examine the regulation of key genes associated with germination. We identified the essential nodes in this process: *DfPIF1*, which maintains the dormancy state in darkness at constant temperatures mainly by promoting ABA biosynthesis and signaling, and antioxidant enzymatic machinery, *DfMSD1*, *DfCSD2*, *DfAPX*, and *DfPRX1*, whose activity regulates ROS homeostasis, promoting or inhibiting germination. This study provides novel mechanisms that regulate seed germination in weeds, specifically involving ABA regulation and ROS in response to environmental stimuli.

## 1 Introduction

The seed germination process is a stage in a plant's life cycle during which the seed, under certain conditions and water availability, resumes metabolic processes that will lead to the establishment of a new generation. Seed dormancy is an adaptive trait that allows seedling survival by avoiding germination at an unsuitable time and place, synchronizing natural plant populations to changing environmental conditions (Footitt et al. 2013; Buijs et al. 2020). It is modulated by environmental signals such as mean temperature, as it conveys information on the season (temporal sensing) (Probert and Sussex 2000; Finch‐Savage and Footitt 2017). Once dormancy decreases, seeds become sensitive to another set of signals related to their spatial environment (spatial sensing), such as light (day/night) and alternating temperatures (Finch‐Savage and Leubner‐Metzger 2006; Finch‐Savage and Footitt 2017). For instance, alternation in white light and temperature indicates that the seed is located at the soil surface with no shading, which signifies adequate conditions for germination and for the establishment of a new generation. For weeds, such responsiveness is critical, as it enables rapid emergence and colonization of open niches (Cristaudo et al. 2007; Catara et al. 2016). Currently, the physiological and molecular processes governing the germinative response of invasive weeds to light and alternating temperatures remain largely unexplored.

Light is perceived through specific phytochromes that are activated by different wavelengths. Phytochrome B is activated by white light and exists in two interconvertible forms: the inactive form (Pr), which predominates in the far‐red wavelength, and the active form (Pfr), which is induced by red light (Sweere et al. 2001; Klose et al. 2015). Phytochrome activity influences seed germination as Pfr migrates into the nucleus and concurs with the sequestration of the PHYTOCHROME‐INTERACTING FACTOR (PIF1), a key transcription factor repressing seed germination in the dark (Oh et al. 2004; Shen et al. 2005). PIF1 directly enhances ABA signaling by interacting with ABSCISIC ACID INSENSITIVE 3 (ABI3), which regulates seed growth and reserve storage (Lepiniec et al. 2018; Baud et al. 2022). Additionally, PIF1 promotes the expression of the *SOMNUS* (*SOM*) gene, which encodes a CCCH‐type Tandem Zinc Finger (TZF) protein that modulates the transcription of Jumonji demethylases (*JMJ20* and *JMJ22*) responsible for the removal of histone methylations at the *GA 3‐OXIDASEs* (*GA3OX1* and *GA3OX2*) gene loci (Park et al. 2011; Cho et al. 2012). On the other hand, PIF1 also acts in other ways, for example, by repressing, together with COP1 (CONSTITUTIVE PHOTOMORPHOGENESIS 1) and SPA (SUPPRESSOR OF PHYA‐105 1 family), photomorphogenesis in the dark (Paik et al. 2019; Han et al. 2020).

The molecular mechanisms regulating seed germination in response to alternating temperatures are considerably less explored than those for light perception. They were described only for the model species *Arabidopsis thaliana* , which is a non‐competitive weed (Finch‐Savage and Footitt 2017). In the last decades, authors assessed that in Arabidopsis, the alternating temperatures mainly affect circadian clock functioning, facilitating dormancy release, likely through the indirect modulation of ABA and GA biosynthesis and their distribution within various embryo regions (Penfield and Hall 2009; Topham et al. 2017). Arana et al. (2017) proposed a working model of the alternate temperature perception system for Arabidopsis seeds based on phytochrome B that requires functioning *PSEUDO‐RESPONSE REGULATOR 7 (PRR7)* and *TIMING OF CAB EXPRESSION 1* (*TOC1*, also known as *APRR1*) clock genes to induce germination. In addition, the LUX ARRHYTHMO (LUX), a transcription factor within the evening clock complex (Nusinow et al. 2011), has been demonstrated to modulate the expression of *DELAY OF GERMINATION1* (*DOG1*), a key regulator in the initiation of seed dormancy (Zha et al. 2020). Furthermore, alternating temperatures in darkness reduced the protein levels of DOG1, allowing the expression of TOC1 to induce germination in Arabidopsis seeds (Arana et al. 2017).

Beyond transcriptional control, epigenetic regulation via histone acetylation influences dormancy and germination by modifying gene accessibility. Among histone deacetylases (HDAs), members of the RPD3 class 1, such as HDA19 and HDA6, redundantly repress seed maturation genes (Tanaka et al. 2008; Zhou et al. 2020). HDA15 has been linked to the repression of *XYLOGLUCAN ENDOTRANSGLUCOSYLASES/HYDROLASES* (XTHs), thereby modulating cell wall loosening (Oh et al. 2009; Gu et al. 2017), while HDA9 acts as a negative regulator of germination by deacetylating photomorphogenesis‐related genes (Van Zanten et al. 2014). In general, the use of HDA inhibitors, such as valproic acid, during seed imbibition represses germination (Tai et al. 2005; Gomez‐Cabellos et al. 2022). Overall, although epigenetic changes clearly contribute to dormancy regulation in response to the environment, their interpretation is complicated by the coexistence of multiple HDAs with distinct target genes.

Reactive Oxygen Species (ROS) are a group of extremely reactive molecules, including superoxide anion (O_2_^•−^), hydrogen peroxide (H_2_O_2_), hydroxyl radical (•OH), and singlet oxygen (^1^O_2_), whose accumulation naturally occurs in the seed during desiccation. Their role in releasing seed dormancy has been largely documented in the last decades in many plant species (Bailly 2004; Oracz et al. 2007; Puglia 2024). For example, in sunflower seeds, their addition resulted in dormancy alleviation, and a crosstalk between ROS and ABA/GA has been proposed (Leymarie et al. 2012; El‐Maarouf‐Bouteau et al. 2015). In *Cynara cardunculus L*., the addition of ROS donors enhanced germination, while it was inhibited by the addition of antioxidants (Huarte et al. 2014; Huarte et al. 2018; Huarte et al. 2020; Puglia et al. 2022). At moderate amounts, ROS may change the ABA and GA ratio by controlling genes involved in their metabolism and signaling, which leads to the promotion of seed germination. For example, they were shown to control HDA10 (Menge et al. 2017), to affect transcript stability of *DELLA GA‐INSENSITIVE* (*GAI*) during after‐ripening (Nelson et al. 2017), and to induce ABA catabolism and GA biosynthesis through the upregulation of *ABA 8′‐HYDROXYLASES* (*CYP707As*), *GA3OXs*, and *GA 20‐OXIDASE* (*GA20OX*) gene expressions (Liu et al. 2010; Ishibashi et al. 2017). However, at high concentrations, they can damage seed tissues with detrimental effects (Bailly et al. 2008). The maintenance of oxidative stress within a safe critical range, namely the “oxidative window of germination” (Bailly 2004), is controlled also by the enzymatic antioxidative machinery, which includes GLUTATHIONE S‐TRANSFERASES (GSTUs) (Wu et al. 2020), ASCORBATE PEROXIDASE 1 (APX1) (Davletova et al. 2005), 1‐CYS PEROXIREDOXIN (PRX1) (Haslekås et al. 2003), Cu/Zn SUPEROXIDE DISMUTASE (CSDs), and Mn‐SOD (MSD1) (Xi et al. 2010), which contribute to preventing oxidative damage (Bailly 2019; Koramutla et al. 2021). Moreover, a cross‐phyla study on peroxiredoxin proteins showed that PRX1 can constitute a universal marker for circadian rhythms by being responsive to their oscillations in a variety of model organisms: Bacteria, Archaea, and Eukaryota (Edgar et al. 2012). Similarly, other ROS antioxidant enzymes, such as MSD and APX, have been shown to have circadian clock‐dependent activity (Jiménez et al. 2021). Therefore, the responsiveness of seeds to temperature alternation could be linked to ROS antioxidant enzymatic machinery activity, providing further evidence for the identification of the role of ROS in dormancy release. However, there are no proposed mechanistic models for weeds that incorporate the action of ROS antioxidant enzymes in the seed dormancy release in response to alternating temperatures and light.

Seed responses to environmental cues can vary not only among species but also within populations of the same species, reflecting local adaptation to contrasting habitats (Fernández‐Pascual et al. 2013; López et al. 2019). Such interpopulation variation in dormancy and germination traits is often overlooked, yet it may provide a critical advantage under fluctuating climates by enhancing the persistence of natural populations (Cochrane et al. 2015). For example, distinct populations of *Polygonum lapathifolium* displayed different light sensitivities along altitudinal gradients (Bhatt et al. 2023). In invasive weeds, this variability can further promote colonization success by broadening the ecological niche for germination. *Dipsacus fullonum* L. subsp. *fullonum* (common teasel), Caprifoliaceae, is a widespread invasive biennial species of disturbed habitats. Previous studies showed that its seeds germinate readily under light or alternating temperatures but remain dormant in constant darkness, unless ROS donors are applied (Huarte et al. 2016). This responsiveness, together with variability among populations, makes *D. fullonum* a promising model for investigating the integration of environmental cues with ROS homeostasis in seed dormancy release. However, the molecular mechanisms governing the achene germination of *D. fullonum* under light or at alternating temperatures remain entirely unexplored, as such investigations are significantly impeded in non‐model species due to the lack of molecular data. Nonetheless, the current advancement of long‐read RNA‐seq technology offers novel opportunities to explore the genetic foundations of unique processes in overlooked plant species (Puglia et al. 2020; Riaño‐Pachón et al. 2021).

The present study aims to extend our understanding of the regulatory mechanisms controlling germination in the invasive *D. fullonum* in response to environmental stimuli. We hypothesized that dormancy release under light and alternating temperatures involves the integration of ROS homeostasis and ABA regulation, potentially modulated by PIF1 and other factors.

## 2 Materials and Methods

### 2.1 Plant Materials and Germination Tests

Achenes (hereafter referred to as seeds) of *D. fullonum* were collected at the natural time of dispersion in the years 2021, 2023, and 2024 from 6 different populations: Simeto (SIM), Etna (ETN), Pietraperzia (PIE), Lomas (LOM), La Plata (LAP), and Bahía (BAH) (Table S1). After collection, the seeds were kept dry at 20°C and 20% RH until the start of the germination tests. All tests were conducted within a month after collection using incubators at an irradiation of 145 μmol m^−2^ s^−1^ as the light condition unless otherwise specified. For all germination tests, we used 3 technical replicates of 50 seeds for each population incubated in 90 mm Petri dishes with 3.5 mL of distilled water or a solution with the specific compound. For all germination experiments, the seeds were monitored daily in light and every 2 days under dim‐green safe light for continuous darkness treatments and were considered germinated when the radicle reached 1 mm in length. Furthermore, to evaluate the effect of the different treatments used, we calculated the final germination percentage and performed a *t*‐test for each condition. To screen the collected accessions with respect to light and temperature regime sensitivity, we used constant temperatures of 5°C, 10°C, 15°C, 20°C, 25°C, 30°C, or 35°C, and corresponding alternating temperatures of 5°C/15°C, 10°C/20°C, 15°C/25°C, or 20°C/30°C (thermoperiod 12/12 h). Regarding the light conditions, we used light/dark (photoperiod 12/12 h) or continuous dark simulated with a double layer of aluminum foils. In order to evaluate the role of phytochrome in the germination process, seeds were imbibed in Petri dishes in water for 1 h and then LED‐irradiated with red (660 nm, 110 μmol m^−2^ s^−1^) or far‐red (730 nm, 37 μmol m^−2^ s^−1^) pulses for 5 min or 24 h. After the LED‐irradiation treatment, the Petri dishes were wrapped in aluminum foil and transferred to 10°C, 15°C, 20°C, or 10°C/20°C.

For hormone and ROS treatments, ROS localization, and molecular analyses, we used the following contrasting conditions: 15°C in continuous dark, hereafter referred to as “dark”; 15°C in light/dark (photoperiod 12/12 h), hereafter referred to as “light”; 10°C/20°C (thermoperiod 12/12 h) in continuous dark, hereafter referred to as “alternating temperatures”.

### 2.2 Effect of Hormones, Fluridone, Histone Deacetylase Inhibitor and ROS on Germination

The effect of GA_4+7_ (gibberellic acid 4 + 7, Duchefa) and fluridone (Sigma Aldrich) was tested in the dark, while ABA (Sigma Chemical Company) was tested in light conditions and at alternating temperatures. Depending on the test, seeds were alternatively imbibed in 1, 10, or 50 μM solution for fluridone; 50, 100, or 200 μM for GA_4+7_ or 1, 5, 10, 25, 50, or 100 μM of ABA. To evaluate the effect of the alteration of ROS homeostasis on germination, we used methyl viologen (MV) (Sigma Chemical Company), an ROS donor, or N‐acetylcysteine (NAC) (Sigma Chemical Company), a thiol‐containing compound that directly scavenges ROS, and diphenyleneiodonium (DPI) (Sigma Chemical Company), an inhibitor of NADPH oxidases (Rboh). For MV treatment, we immersed seeds for 3 h at 0.1, 0.25, 0.4, or 0.5 mM (Oracz et al. 2007; El‐Maarouf‐Bouteau et al. 2015). After the treatment, seeds were rinsed with distilled water and transferred to Petri dishes at 15°C in complete darkness. In contrast, for NAC or DPI, we imbibed seeds in 10, 20, or 25 mM solution of NAC or 0.1, 0.3, or 0.5 mM DPI. The effect of the inhibition of HDAs on germination was tested by performing germination tests in the presence/absence of light and at alternating/constant temperatures, imbibing seeds either with 10 mM of valproic acid (Gomez‐Cabellos et al. 2022) (Sigma Chemical Company) or water (control).

### 2.3 In Situ ROS Localization

ROS localization was performed using embryos dissected from seeds imbibed for 45 h (i.e., the time to reach 1% of total germination in the most stimulating condition) in dark, light, or alternating temperatures. For the superoxide anion localization, the embryos were immersed in a solution of 0.2% Nitro Blue Tetrazolium (NBT, Sigma Aldrich) in 50 mM sodium phosphate buffer (pH 7.0) and incubated for 20 min at room temperature in complete darkness. For the visualization of the accumulation of H_2_O_2_, the seeds were immersed in a solution of 1 mg/mL of 3,3′‐Diaminobenzidine tetrahydrochloride (DAB, Sigma Aldrich), pH 3.7, for 4 h in complete darkness. In both cases, the embryos were washed three times with distilled water and then photographed using the stereo microscope (Leica M205 C).

### 2.4 LC‐MS/MS Measurement of ABA Levels

For ABA analysis, seeds were incubated as for the ROS in situ localization assay, then collected during the dark photoperiod, frozen in liquid nitrogen and ground into a fine powder. When all the required material had been collected, the samples were lyophilized for 48 h. The extraction method used was based on the procedure described by Zhou et al. (2003) with some modifications as reported below. Around 100 mg for each sample was ground up and extracted for 6 h at 4°C at 2773 × *g* with 1.5 mL of acetone‐water‐acetic acid (80:20:1, v/v) in a thermomixer (Thermomixer C, Eppendorf). Afterwards, the samples were centrifuged at 11,148 × *g* for 15 min, the supernatants were collected, and the residual pellets were re‐extracted for 15 h at 4°C overnight. The second group of extracts was centrifuged, and then the supernatants were combined and dried under a nitrogen stream. Finally, the evaporated samples were reconstituted in 1 mL of acetonitrile‐water‐acetic acid (80:19.5:0.5, v/v), filtered through a 0.22 μM nylon filter, and 5 μL of each sample was then injected onto a reversed‐phase column C18 (Hypersil Gold 3 μM 100 × 2.1 mm; ThermoFisher) coupled to a Triple Quadrupole Mass Spectrometer (TSQ Fortis; ThermoFisher). Chromatographic separations were conducted using a linear gradient at a flow rate of 300 μL/min of water with 12 mM acetic acid (A) and acetonitrile (B), from 85:15 A:B (v/v) to 100 (v/v) over 7 min. Finally, the column was washed with 100% B (5 min) and equilibrated to initial conditions (85:15 A:B, v/v) for 4 min. The retention time of ABA was 5.85 ± 0.02 min. MS and MS/MS experiments were performed in ESI (+) ion mode with the following settings: capillary voltage 3200 V, sheet gas (N_2_) 30 (arbitrary units), auxiliary gas (N_2_) 5 (arbitrary units), capillary temperature 275°C, and vaporization temperature 250°C. Full scan data acquisition was performed by scanning from *m*/*z* 50 to 500 in profile mode. In product ion scan experiments, MS/MS product ions were produced by collision‐induced dissociation (CID) of selected precursor ions with 28 eV energy. MRM acquisition was carried out through monitoring the 265.1–135.1 transition. The MS data were processed through Xcalibur 4.0 (ThermoFischer Scientific). ABA was quantified using an ABA calibration curve with an ABA standard solution (Sigma Aldrich) following the method reported by Perin et al. (2018). A good linearity was obtained in the range 2–500 ng/mL with an *R* ^2^ value of 0.9987 (Figure S1).

### 2.5 ONT‐Nanopore RNA Sequencing

As for ABA analysis, RNA was isolated from seeds incubated as for the ROS in situ localization assay, then collected during the dark photoperiod and frozen in liquid nitrogen and ground into a fine powder. We used approximately 50 mg of starting material extracted following the RNeasy PowerPlant Kit with DNase treatment (Qiagen). The quality and integrity of RNA were measured with the Eppendorf BioSpectrometer and QIAxcel RNA QC Kit (Qiagen), respectively. For the transcriptome sequencing, we used ONT‐Nanopore technologies with 50 ng of total RNA following the cDNA‐PCR sequencing protocol (SQK‐PCS109, ONT‐Nanopore). We took into account different conditions in order to collect the widest range of expressed transcripts to be used for annotation and gene expression analysis. The treatments we considered included dark, light, alternating temperatures in the dark, and alternating temperatures with light. The samples were loaded on the SpotON flow cells inserted in the MinION Mk18, and then the sequencing was monitored with the MinKnow software for 72 h. The data obtained in the fast5 format were processed with the software Guppy (4.2.2 version) using a server of Google CoLab in super accuracy (SUP) base calling mode.

### 2.6 Transcriptome Assembly and Functional Annotation

Since the genome sequence of *D. fullonum* is not available, we followed a dual approach, mapping the RNA‐seq reads with a genome reference‐guided mode to the closest phylogenetically related species within the Caprifoliaceae plant family, while the unmapped reads were de novo assembled. For the genome reference‐guided approach, we aligned reads to the *Lonicera japonica* Thumb. genome (NCBI accession number ASM2146441v1) with IsoQuant 3.6.2 in the annotation‐free mode (Prjibelski et al. 2023). The de novo assembly was obtained using RNA‐Bloom 2.0 with the ONT read assembly mode (Nip et al. 2020). The two transcriptomes were combined and optimized using the CD‐HIT tool to reduce redundancy (Fu et al. 2012), the EvidentialGene tr2aacds function to select the best isoforms (coding‐aware coding) and remove transcript fragments or duplications (Gilbert 2013), and the ONT long reads to find potential chimera transcripts. The completeness of the obtained transcriptome was assessed using BUSCO 5.6.0 (Seppey et al. 2019), selecting Viridiplantae as lineage and a BLAST *e*‐value < 1e‐15. Additionally, the transcriptome was functionally annotated using the OmicsBox suite version 3.4.6 (https://www.biobam.com/omicsbox/) with BLASTx, applying an *e*‐value < 1e‐15 for Viridiplantae and utilizing Goa version 2025.03 for mapping gene ontology (GO) terms (available upon request). Annotated sequences were searched against Plant Reactome Pathways (Naithani et al. 2020) and their representation was analyzed with Fisher's test to identify enriched pathways.

### 2.7 Differential Gene Expression and Enrichment Analysis

Read quantification was performed using the Salmon tool (Patro et al. 2017) with the ONT reads optimization option. Pairwise differential expression analysis was performed using the NOISeq package (Tarazona et al. 2015), and it was used to perform the enrichment analysis of GO terms using Fisher's exact test to find over‐ and under‐represented terms within the different comparisons using the total transcriptome annotation as the reference, and only GO terms with an adjusted *p*‐value < 0.05 were considered.

### 2.8 Gene Prediction Annotation

From the transcriptome assembly, we selected target sequences based on the annotation results. We focused on sequences that translated into proteins known from literature to be mostly related to the production and signaling of ABA and GAs and the maintenance of ROS homeostasis. To confirm their function, these transcripts were translated into amino acid sequences with the Expasy translate tool and then aligned with the first hits of BLASTx with MAFFT software (Katoh et al. 2002). The specific domains of the proteins were analyzed with CD‐Search of the Conserved Domain Database (CDD) (Wang et al. 2023). From these analyses, we obtained 23 sequences to use for gene expression analysis (File S1).

### 2.9 Gene Expression Analysis

For the gene expression analysis, the total RNA was extracted following the same method used for the ONT‐Nanopore sequencing explained before. The cDNA was obtained using the iScript cDNA Synthesis kit (Bio‐Rad), while RT‐qPCR was carried out on a CFX Opus 96 Real‐Time PCR System (Bio‐Rad) with 1–10 ng of sample using the PowerUp SYBR Green Master Mix (Applied Biosystems) protocol. The set of primers used in this study was specifically designed for each target gene using the PABLOG tool (Ferrigno et al. 2024), which generates primers in the exon‐intron junction. The list of primers is shown in Table S3. Primer pairs were tested at different temperatures with a gradient qPCR to evaluate their specificity and functionality and to determine the optimal annealing temperature (Ta). From the genes analyzed, we selected two constitutively expressed genes as references: *ACTIN4* and *ELONGATION‐FACTOR 1 (EF1)*. For each gene, the expression level under different conditions is presented as a fold change relative to the level of the LOM seeds imbibed at constant temperature (15°C) in the dark, where the mean expression was set to a value of 1. Furthermore, for the statistical significance among conditions, we performed a Student's *t*‐test (*p* < 0.05 *, *p* < 0.01 **, *p* < 0.001 ***) using the same LOM condition.

## 3 Results

### 3.1 Sensitiveness of Different *D. fullonum* Accessions to Light and Temperature Fluctuation

**FIGURE 1:**
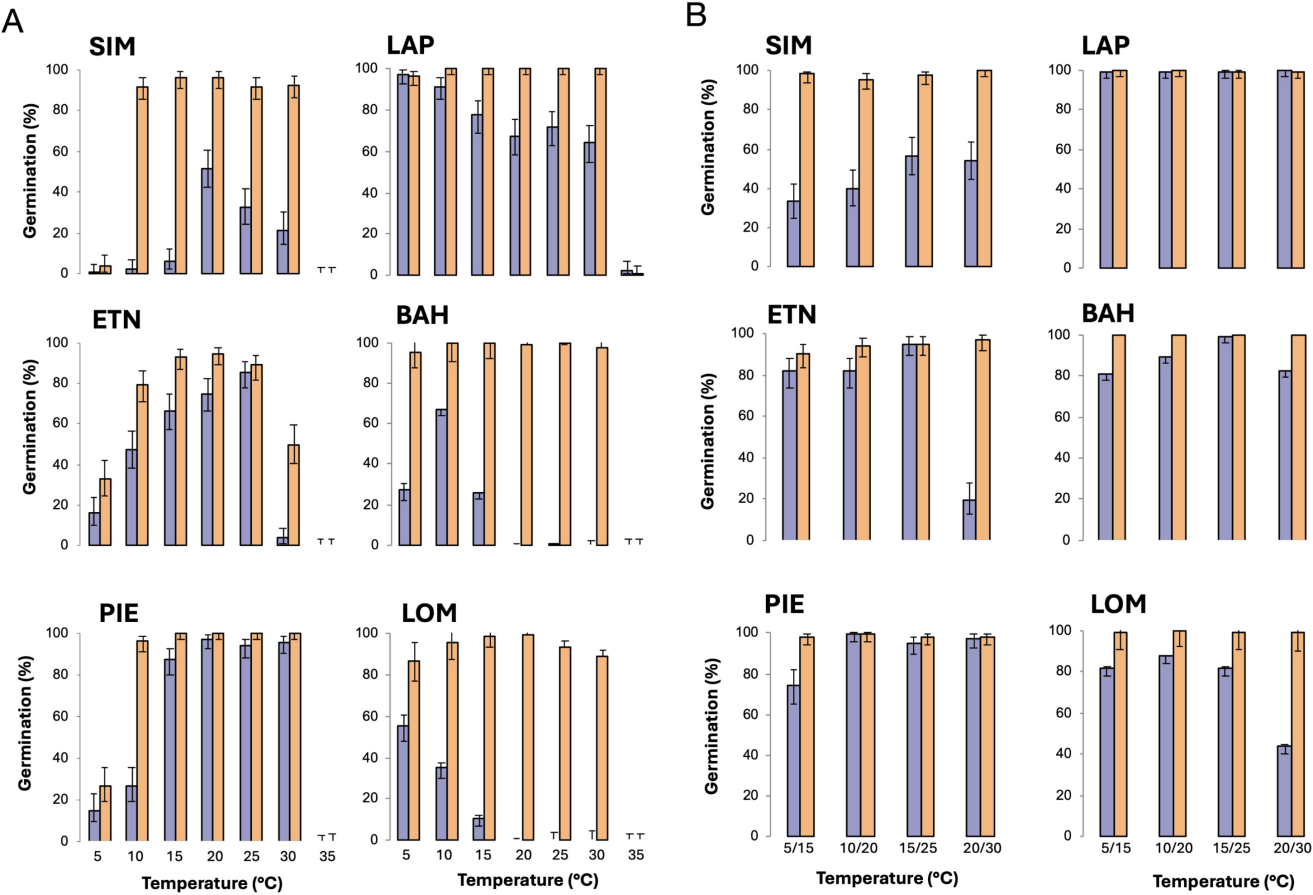
Germination behavior of different *D. fullonum* accessions: SIM, LAP, ETN, BAH, PIE, and LOM. Mean final germination (%) of seeds imbibed at constant temperatures (A) or at alternating temperatures (B) in L/D (yellow bars) (12 h of photoperiod) or in continuous dark (violet bars). Error bars represent confidence intervals calculated over 150 seeds.

Germination data revealed significant differences among the sampled populations in response to the light and temperature conditions considered in this study (Figure 1). All the sampled accessions, for most of the temperatures tested, did not show significant variation in germination percentage when they were imbibed to constant or alternating temperatures if exposed to light. Furthermore, at the extreme temperature of 35°C, the germination was inhibited regardless of light presence for all the populations, while we observed two main different behaviors at 10°C–25°C imbibition temperatures: accessions sensitive to light, whose germination progressively declined following the gradient LOM>BAH>SIM, and accessions whose germination was only partially (not significantly) inhibited (neutral) by the darkness following the gradient ETN>PIE>LAP (Table S2). In addition, for seeds imbibed in the dark, the germination response to alternating temperatures showed a similar pattern compared to constant ones, with the germination of light‐sensitive accessions being highly promoted by alternating temperatures (LOM>BAH>SIM) compared to neutral accessions, whose germination was only partially enhanced (ETN>LAP>PIE) (Table S2). Thus, among the sampled accessions, LOM proved to be highly sensitive to light and temperature alternation, and, for this reason, we only used seeds from the LOM accession to deepen the investigation of *D. fullonum* 's germination response to these environmental signals. This pattern was further confirmed by the germination tests carried out with seeds sampled at the same sites over 3 years (Figure S2).

**FIGURE 2:**
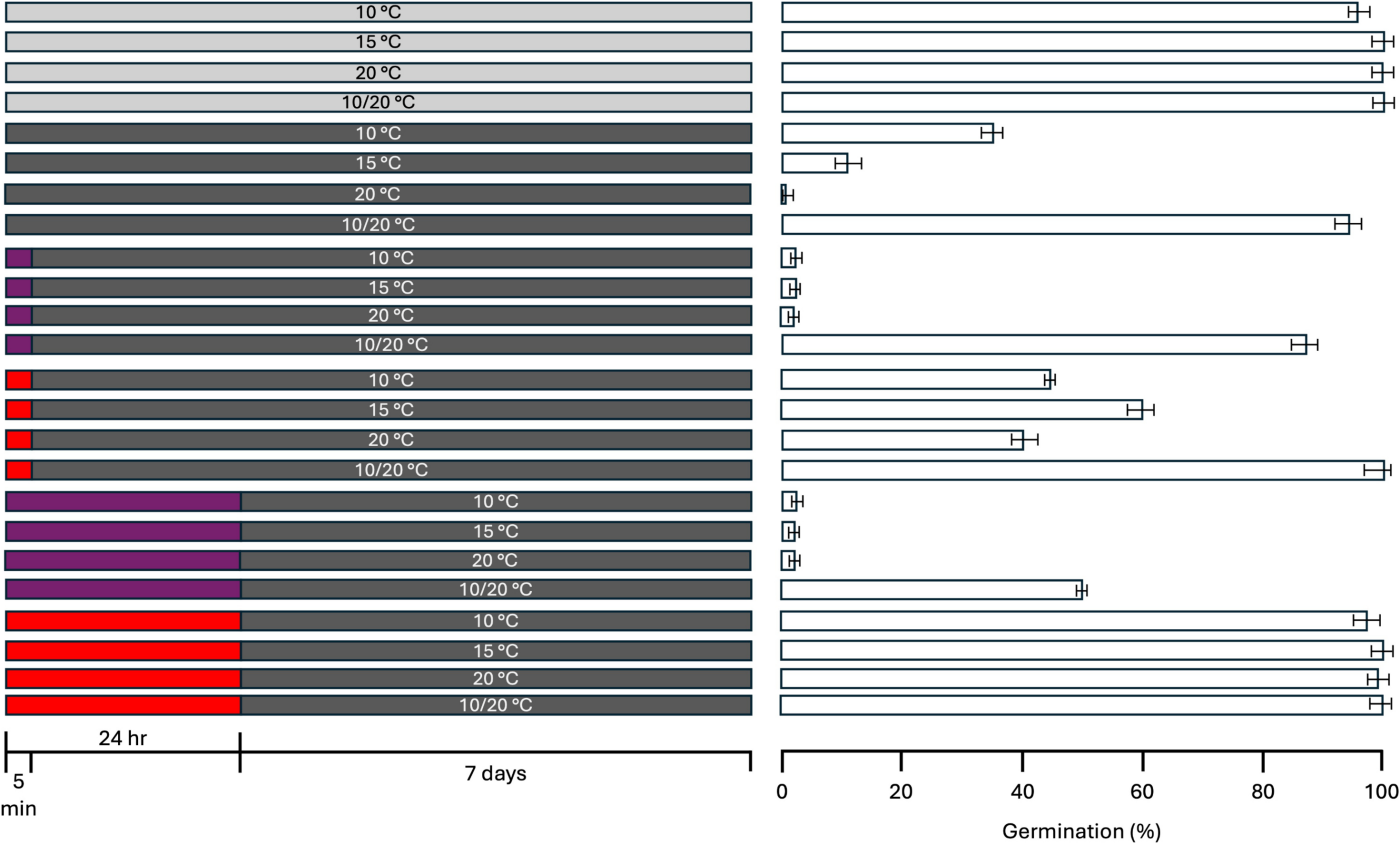
Seed germination of LOM seeds with red or far‐red pulse irradiation. Left panel, irradiation treatments used for the experiment. Seeds were imbibed at the indicated temperatures in the L/D 12 h photoperiod (light grey) or in the continuous dark (dark grey) or with a preliminary exposure to far‐red (purple rectangles) or red light (red rectangles). Small rectangles indicate 5 min of exposition, while large ones indicate 24 h of exposition. Right panel, mean final germination percentage of seeds imbibed at the corresponding conditions described in the left panel. Error bars represent confidence intervals calculated over 150 seeds for each treatment.

To investigate the role of phytochrome in the mediation of germination of LOM seeds in relation to light and alternating temperatures, we tested their germination in response to far‐red and red pulse pretreatment, which promote the Pr and Pfr forms, respectively (Figure 2). When seeds were exposed to a short (5 min) far‐red pulse, germination was totally inhibited at constant temperatures, while the presence of alternating temperatures almost partially reconstituted the germination percentages compared to imbibition in the dark without any treatment. The longer (24 h) exposure to the far‐red pulse caused a stronger inhibition of germination at the alternating temperatures, although 48% of germination was reached. On the other hand, a 24 h red‐light pulse resulted in the complete restoration of germination, while the short exposure (5 min) resulted in intermediate germination percentages compared to light and dark. This indicates that the induction of germination at constant temperatures is largely dependent on phytochrome activation, while its contribution is likely to be only partial at alternating temperatures.

### 3.2 ROS Homeostasis Affects Germination Response

**FIGURE 3:**
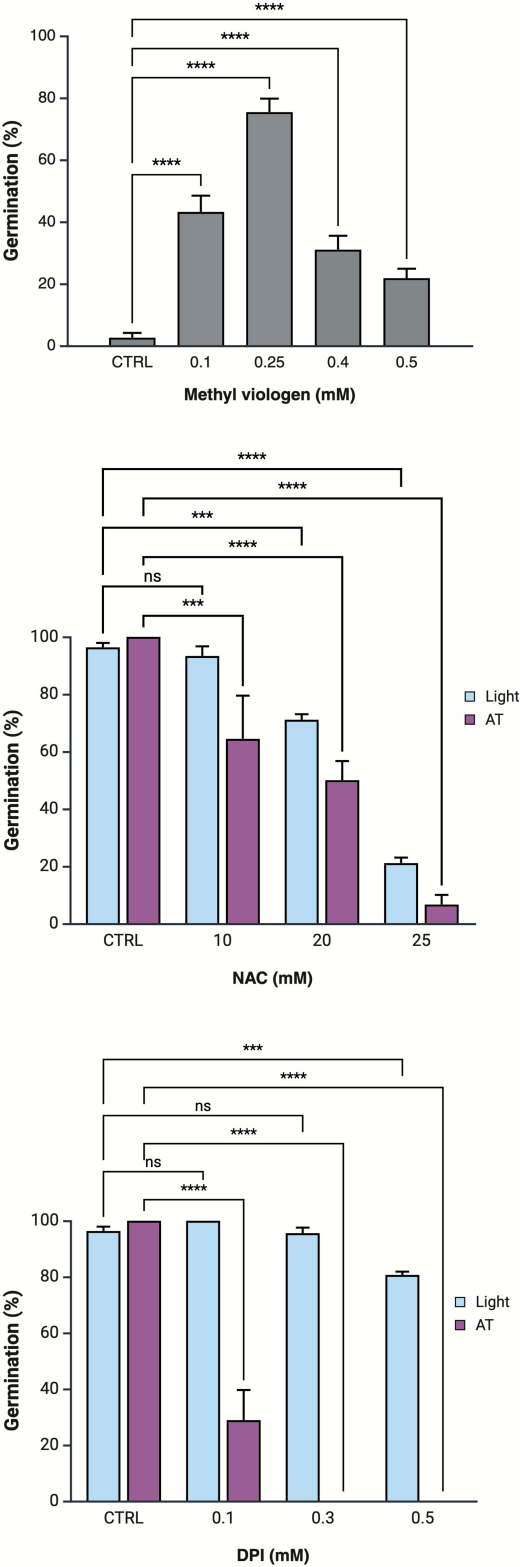
Comparison of the effect of the addition of a ROS donor or inhibitors on germination of LOM seeds. Mean final germination of LOM seeds imbibed in the dark at constant temperature in the presence of MV (uppermost panel) or imbibed in the light at constant temperature with NAC (central panel) or DPI (lowermost panel) at different concentrations (or no treatment: CTRL). Error bars represent confidence intervals calculated over 150 seeds for each treatment.

Pretreatment with MV between the range of 0.1 and 0.5 mM highly favored the germination response of LOM seeds (Figure 3). Outside this range, germination was inhibited, indicating the overcoming of the safe oxidative window. With the pretreatment of 0.25 mM MV, germination of LOM seeds in the dark at constant temperature was similar to that scored in the light at the same temperature regime, indicating that, in the absence of light, moderate ROS accumulation effectively promotes germination. Furthermore, the addition of different amounts of NAC, a ROS scavenger, progressively affected the germination percentages, resulting in almost complete inhibition of germination at 25 mM (Figure 3). In general, its effect was more pronounced in the seeds exposed to dark at alternating temperatures, claiming that at this condition, the ROS homeostasis is the crucial driver for dormancy release. This pattern was more marked in the presence of DPI, an inhibitor of NADPH oxidases (i.e., Rboh) that plays a key role in ROS production. DPI addition produced the complete inhibition of germination even at 0.3 mM, while in the presence of light, germination was still not compromised at 0.5 mM of DPI. This further confirms the relationship between ROS level regulation and dormancy release in the dark at alternating temperatures.

**FIGURE 4:**
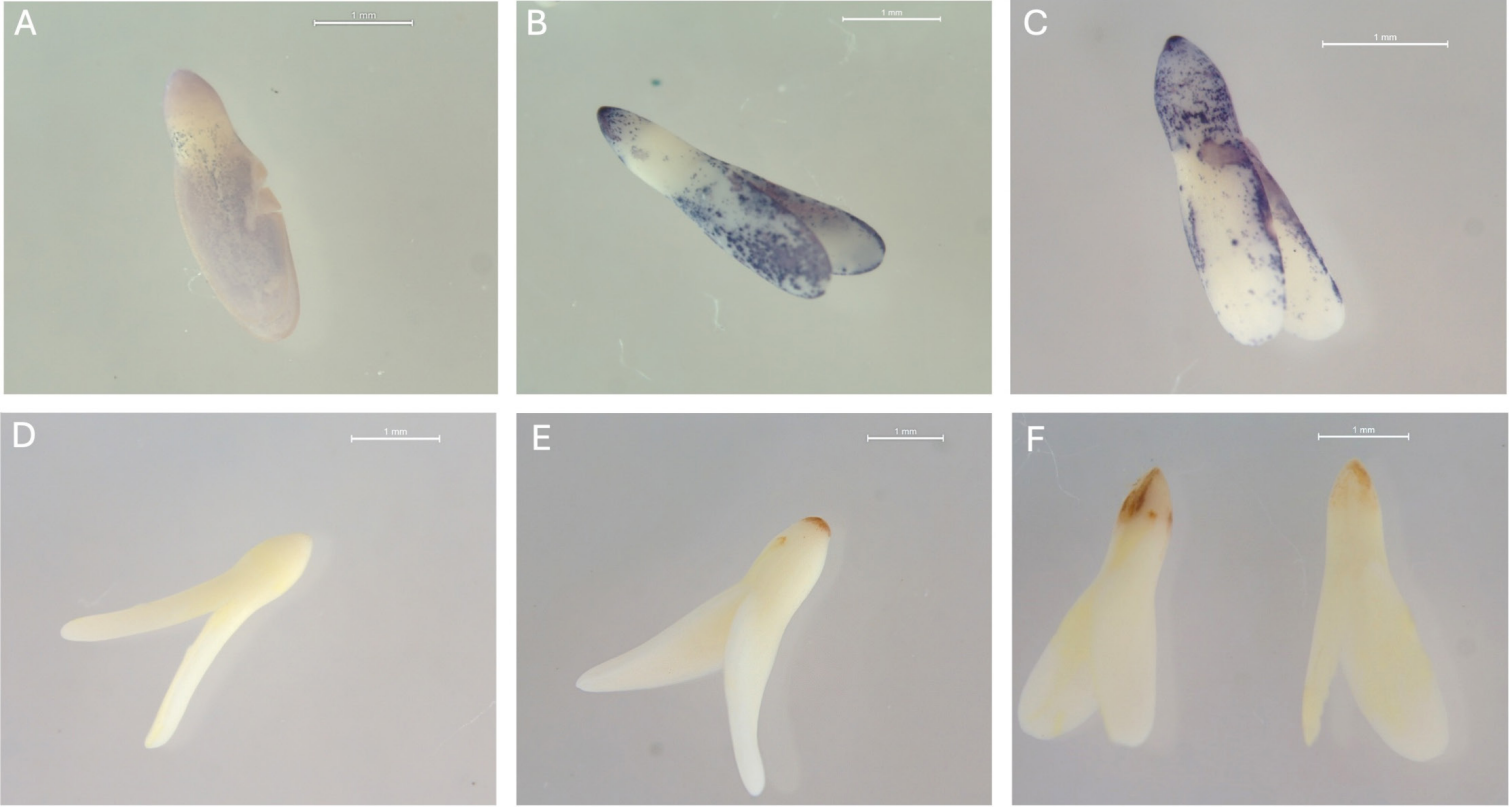
In situ localization of superoxide anions (NBT) and H_2_O_2_ (DAB) in excised embryos of LOM accession. (A) NBT staining in seeds imbibed in the dark, or in the light (B), or at alternating temperatures (C), respectively. (D) DAB staining in seeds imbibed in the dark, or in the light (E), or at alternating temperatures (F), respectively.

To further elucidate the link between ROS generation and dormancy release, we analyzed their appearance on excised embryos when imbibed in light/dark or at constant/alternating temperatures (Figure 4). In LOM accession, the presence of both NBT‐ and DAB‐stained spots was observable mostly at the radicle tip in embryos imbibed in light (both constant and alternating temperatures) and in the dark only at alternating temperatures, while no clear staining was visible on embryos imbibed in the dark at 15°C. This finding shows that both superoxide anions and H_2_O_2_ accumulate in *D. fullonum* seeds when optimal conditions are met for dormancy release.

### 3.3 ABA and GA Levels Variation in the Dormancy Release

**FIGURE 5:**
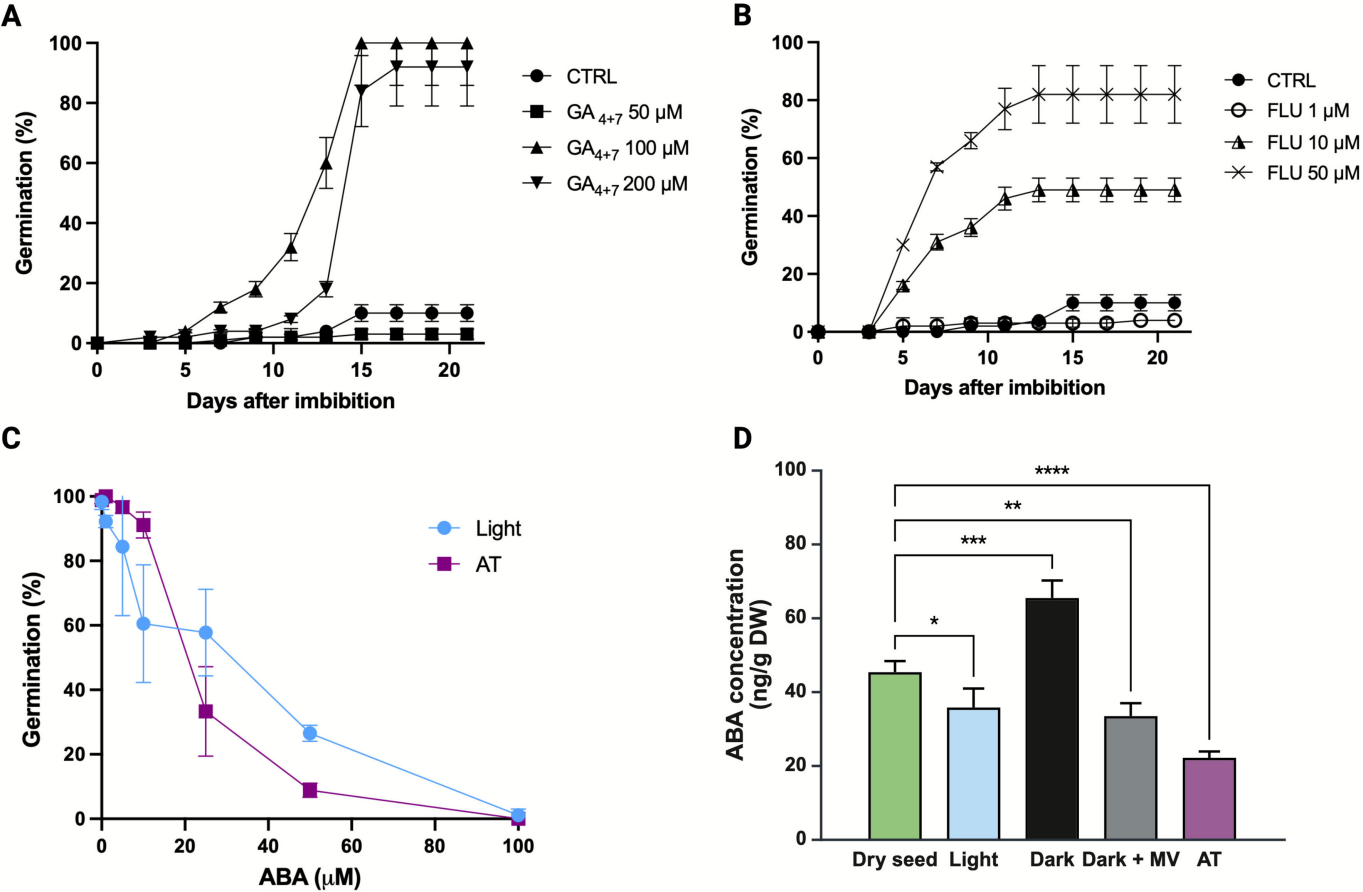
Seed germination response in the presence of gibberellins, fluridone, or ABA and quantification of ABA. Time course germination of LOM seeds imbibed at 15°C in the dark with water (CTRL) or with 50, 100, or 200 μM of GA_4+7_ (A) or with 1, 10, or 50 μM of fluridone (FLU) (B). Final germination percentage means of LOM seeds in the presence of 1, 5, 10, 25, 50, or 100 μM of ABA incubated in L/D at 15°C or 10°C/20°C (C). Error bars represent confidence intervals calculated over 150 seeds for each treatment. (D) ABA concentrations in ng g^−1^ dry weight (DW) in LOM dry seeds (green), seeds imbibed at constant temperature in light (light blue), in darkness (black), in darkness after MV treatment (grey) and in darkness at alternating temperatures (purple). Error bars represent standard deviation.

To investigate the ABA and gibberellin role in the regulation of seed germination in relation to light and/or temperature regime, we imbibed the seeds in the presence of ABA, GA_4+7_, or fluridone, an inhibitor of carotenoid biosynthesis required for de novo ABA synthesis (Figure 5). In the dark, the addition of fluridone and GA_4+7_ resulted in almost full germination (Figure 5). Nevertheless, fluridone was more effective than gibberellins in favoring germination, as its addition resulted in a higher and faster response at lower concentrations. This lag time between the two treatments can be due to the action of fluridone, which is an inhibitor of de novo ABA synthesis, highlighting the importance of de novo synthesized ABA in the control of germination of LOM seeds in the dark. However, the presence of GA_4+7_ ultimately resulted in germination completion because of the alteration of ABA/GA in favor of GA. Moreover, the analyses of ABA showed that the highest accumulation of this hormone occurs in the seeds imbibed in the dark, while the exposure to light or alternating temperatures determined a decrease in the amount of ABA compared to dry seeds (Figure 5). Also, when dark‐imbibed seeds were first treated with MV, ABA levels dropped significantly compared to seeds that had not been treated. This pattern could provide further evidence for the hypothesis that ABA production in LOM seeds is stimulated by the concomitant presence of dark and constant temperatures.

### 3.4 Effect of Histone Deacetylase Inhibitor

**FIGURE 6:**
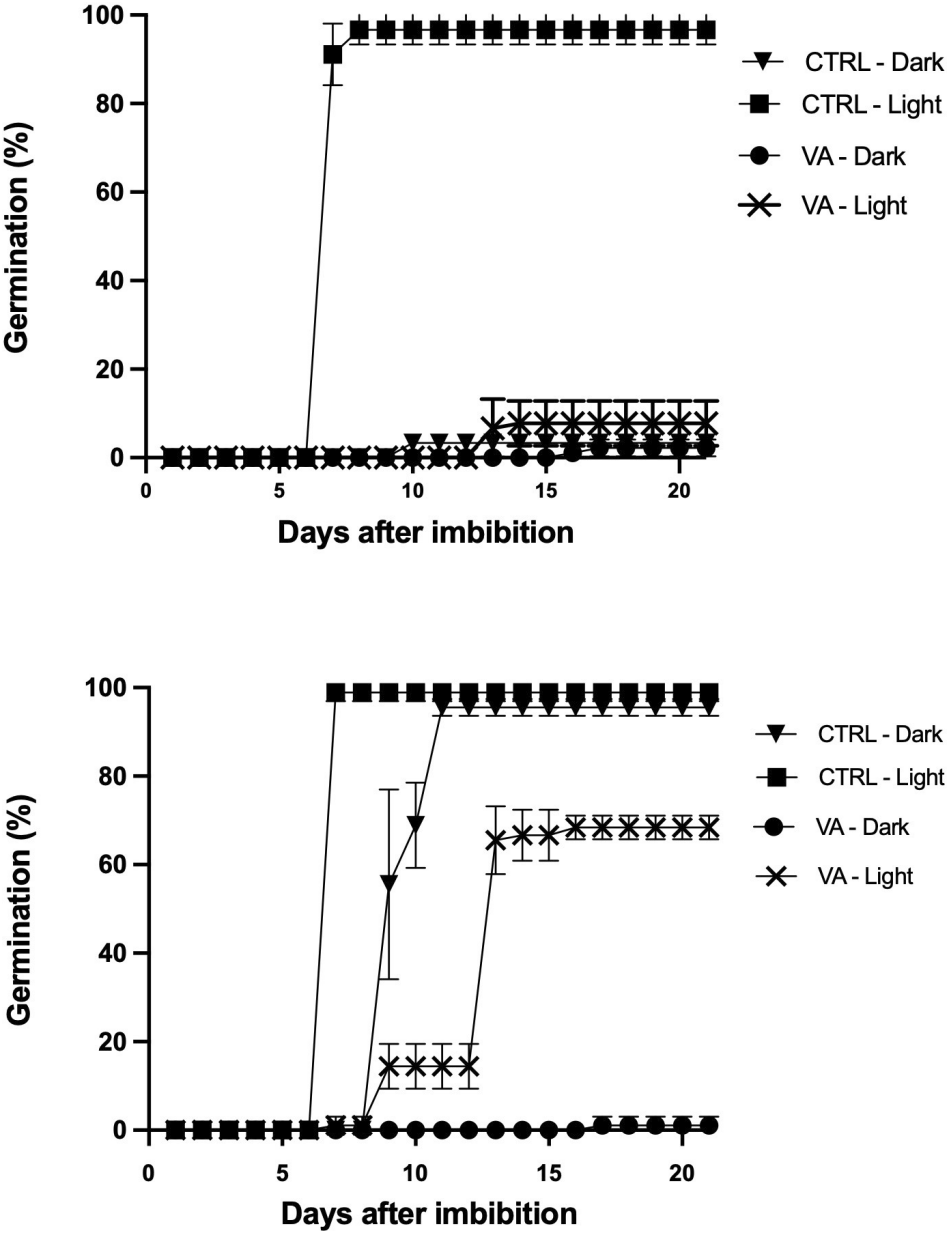
Time course germination of LOM seeds imbibed with 10 mM of valproic acid (VA) or water (CTRL) in the presence/absence of light at constant (upper panel) or alternating temperatures (lower panel). Error bars represent confidence intervals calculated over 150 seeds for each condition.

We exposed LOM seeds to valproic acid (Figure 6) to elucidate the possible role of HDAs in their dormancy release. At a constant temperature, the addition of this compound inhibited germination, even in the presence of light. In contrast, at alternating temperatures in the presence of light, 64% germination was achieved, but it was completely impaired for dark‐imbibed seeds even with alternating temperatures. Overall, this pattern suggests a link between massive HDAs inactivation and germination inhibition.

### 3.5 Darkness and Alternating Temperatures Affected Transcriptome Composition in LOM Seeds

**FIGURE 7:**
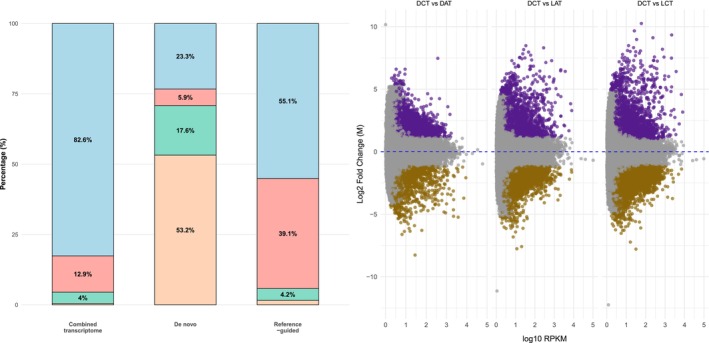
Analysis of transcript assemblies. The left panel displays the BUSCO completeness assessment for conserved orthologous genes in the combined, de novo, and genome reference‐guided transcriptomes. Light blue: Complete single‐copy; pink: Complete duplicated; light green: Fragmented; light orange: Missing genes. On the right, MA plots showing the DEGs identified in the three comparisons (DCT vs. DAT, DCT vs. LCT and DCT vs. LAT). Fold change > 2, FDR < 0.01.

**FIGURE 8:**
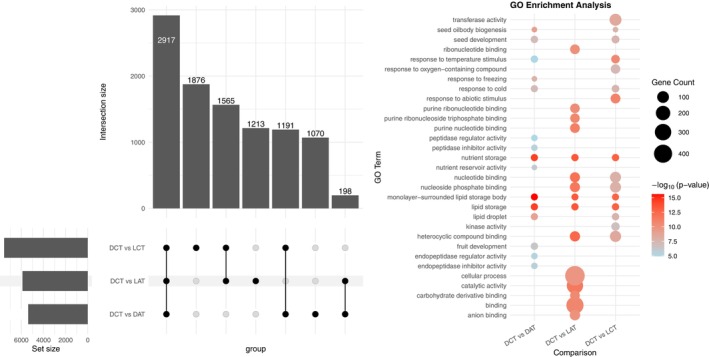
Analysis of identified DEGs. On the left panel, the upset plots of the overlapping DEGs across multiple comparisons. On the right, the bubble plot of top GO enrichment analysis showing the over‐represented terms in the three comparisons (DCT vs. DAT, DCT vs. LCT and DCT vs. LAT); the full list is provided as Set S2.

We carried out an ONT‐Nanopore RNA‐seq analysis of LOM seeds imbibed at constant or alternating temperatures in the dark or in the light to obtain a repertoire of expressed transcripts governing the germination response in relation to these environmental cues. A total of 28,331,375 reads were obtained with a mean *N50* value of 759.5 bp. With the genome reference‐guided approach, 23,942,234 reads were mapped to the *L. japonica* genome, forming 42,003 sequences with an *N50* value of 1452 bp. With the rest of the reads, we generated a de novo assembly including 100,746 sequences with an *N50* value of 985 bp. The combined transcriptome consisted of 81,338 sequences with an *N50* value of 1223 bp. From the BUSCO completeness assessment of the final transcriptome, the complete single‐copy sequences accounted for 82.59%, while the complete duplicated sequences accounted for 12.94%, the fragmented sequences for 4%, and the missing sequences were 0.47% of the total (Figure 7). The functional annotation provided GO annotations for 45,787 sequences, which were used as a reference for enrichment analyses. Differentially expressed genes (DEGs) were identified in the three comparisons (DCT vs. DAT, DCT vs. LCT and DCT vs. LAT) (Figure 7). A large number of DEGs were identified in DCT vs. LCT (a total of 4993 genes: 3135 upregulated and 1858 downregulated) and in the DCT versus LAT comparison (a total of 3814 genes: 2170 upregulated and 1644 downregulated); in contrast, in DCT versus DAT, the number of DEGs was less than the other two groups (a total of 3340 genes: 2390 upregulated and 950 downregulated). The analysis of overlapping DEGs showed that the largest intersection was attained when we considered the three comparisons (2717): an intermediate size was observed with the DCT versus LAT and DCT versus LCT comparison (1731) and with DCT versus DAT and DCT versus LCT (1256). In DCT versus DAT and DCT versus LAT, only 141 genes were commonly differentially expressed (Figure 8, Dataset S1).

The GO enrichment analysis conducted to identify overrepresented terms associated with DEGs with respect to the annotated transcriptome revealed significant enrichment for genes associated with stress response, oxidative homeostasis, and response to abscisic acid. Out of the total enriched GOs, the top terms are shown in Figure 8 (the full list is provided as Dataset S2). The mapping of the differentially expressed pathways to the Plant Reactome database identified 13,428 pathways (Dataset S3) with three enriched pathways: (1) flavin biosynthesis that catalyzes a broad spectrum of vital reactions, (2) ABA‐mediated signaling, and (3) the HSFA7/HSFA6B‐regulatory network induced by drought and ABA.

### 3.6 Gene Expression Analysis

**FIGURE 9:**
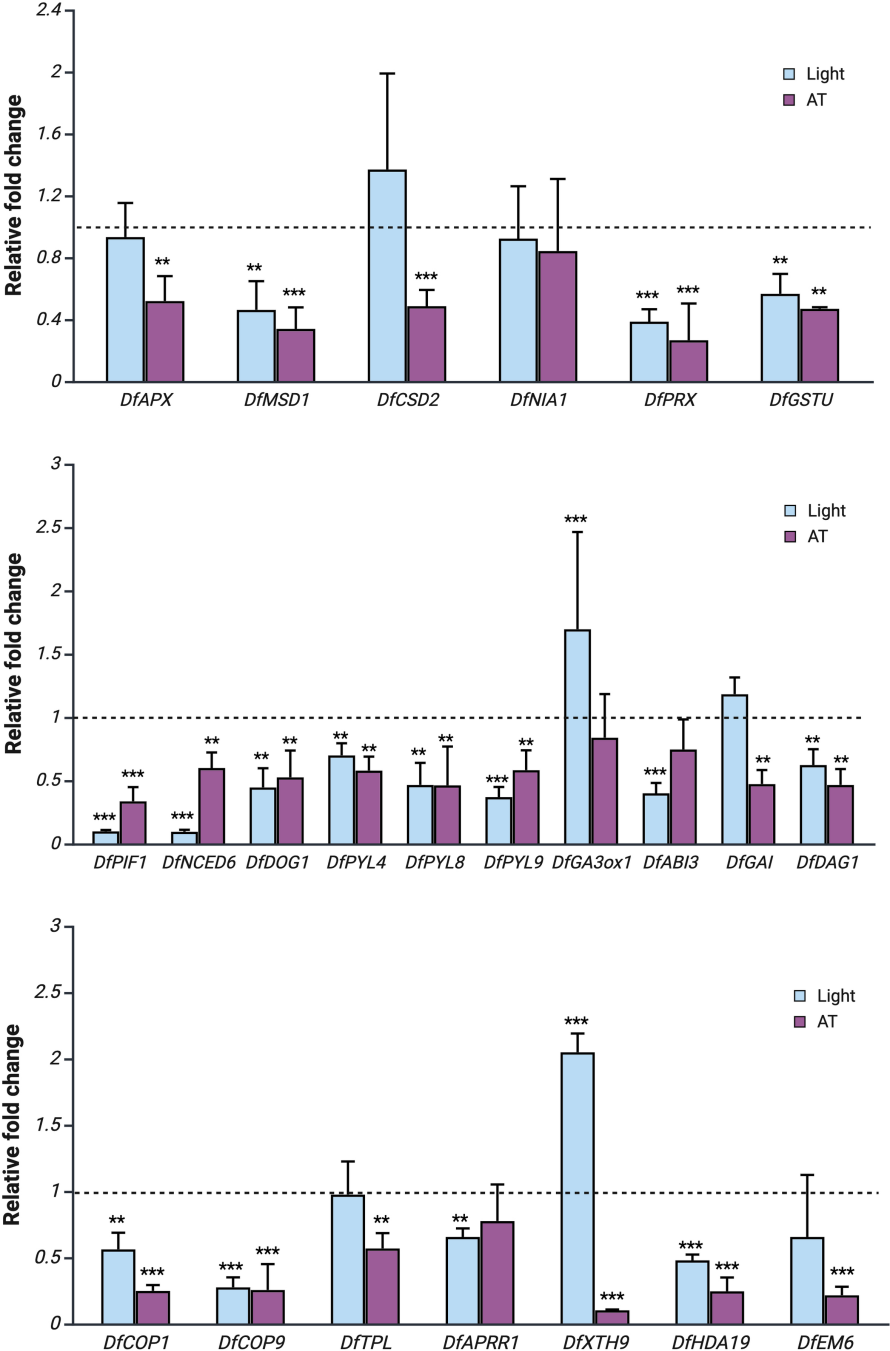
RT‐qPCR analyses of selected genes. LOM seeds imbibed at constant temperature in light (light blue), in the dark (dashed bar), or at alternating temperatures in the dark (purple). The expression levels are presented as fold change relative to the average expression level in the dark at constant temperature, which is represented by a dashed bar. Significance level: *p* < 0.05; *p* < 0.01 **; *p* < 0.001 ***.

Based on the GO enrichment composition, we selected 23 DEGs related to the seed's perception of environmental stimuli, ABA/GA regulation, and ROS homeostasis to be measured by RT‐qPCR. Their annotation was confirmed by phylogenetic analyses with closely related phylogenetic species and model organisms (File S1), and the obtained *D. fullonum* gene sequences were used for primer designing (Table S3). Regarding the ROS homeostasis‐related genes, they were generally upregulated in the germination‐inhibiting condition with respect to others, with *DfPRX1* and *DfMSD1* exhibiting the most marked differences, while *DfAPX, DfGSTU*, and *DfCSD2* were significantly downregulated only at alternating temperatures and had no significant difference in expression between seeds imbibed in light or dark (Figure 9). Among the genes associated with ABA biosynthesis and signaling, *DfNCED6*, *DfABI3*, *DfPIF1, DfPYL4*, *DfPYL8*, *DfPYL9*, and *DfDOG1* were upregulated in seeds exposed to darkness compared to the ones incubated at alternating temperatures or light. *DfPIF1* and *DfNCED6* showed the most marked up‐regulation (*p* < 0.001). As for the GA signaling‐related genes, *DfDAG1* was upregulated in the inhibiting condition (i.e., dark), while the highest expression of *DfGAI* was observed in the light condition, although with a slight difference (*p* < 0.05). Regarding genes related to the perception of light, *DfCOP1* and *DfCOP9* were highly expressed in seeds exposed to darkness, with decreasing values in light and to alternating temperatures, respectively. A similar trend was observed in *DfTPL*, while *DfAPRR1* was significantly downregulated only in seeds imbibed in light. We also measured the expression of *DfHDA19*, *DfEM6*, and *DfXTH9*, which are genes that do not fall in previous categories but were included in the DEGs. *DfHDA19* was significantly upregulated in seeds imbibed in the dark compared to other conditions, while for *DfEM6*, the difference with the light condition was less marked. Lastly, *DfXTH9* was significantly upregulated in seeds imbibed in light and significantly downregulated in the ones at alternating temperatures.

## 4 Discussion

Light and alternating temperatures both provide seeds with important information about their surroundings, such as their burial depth or the presence of competitors. Consequently, it is important for a seed to be able to recognize these signals so that it can germinate in the most appropriate conditions for the establishment of a new plant generation. In the present study, we found substantial differences in seed dormancy release with respect to light and alternating temperatures among accessions (interpopulation) of the species *D. fullonum* , a typical invasive weed of disturbed environments spread worldwide. Two types of germination behavior were found: one indifferent to the presence of light and alternating temperatures and another positively stimulated by their presence. In fact, seeds of ETN, PIE, and LAP accessions exhibited very high germination percentages even in the dark or at constant temperature. On the other hand, SIM, LOM, and BAH exhibited inhibition of germination in the dark but showed a full response in the presence of light or alternating temperatures. Interpopulation variation in seed traits is often disregarded when describing the seed germination behavior of a species, although it is important to report germination behavior heterogeneously, as it enhances the ability of species to persist against climatic variability (Cochrane et al. 2015). In *Polygonum lapathifolium* , the interpopulation variation in physiological traits included different germination responses to light, and it was proposed to be related to altitudinal gradients in population sites (Bhatt et al. 2023). Here, we do not have enough evidence to identify the basis for this interpopulation variability, but we speculate that, for *D. fullonum*, each population has experienced different natural selection pressures, and thus it may have led to local adaptation over time.

**FIGURE 10:**
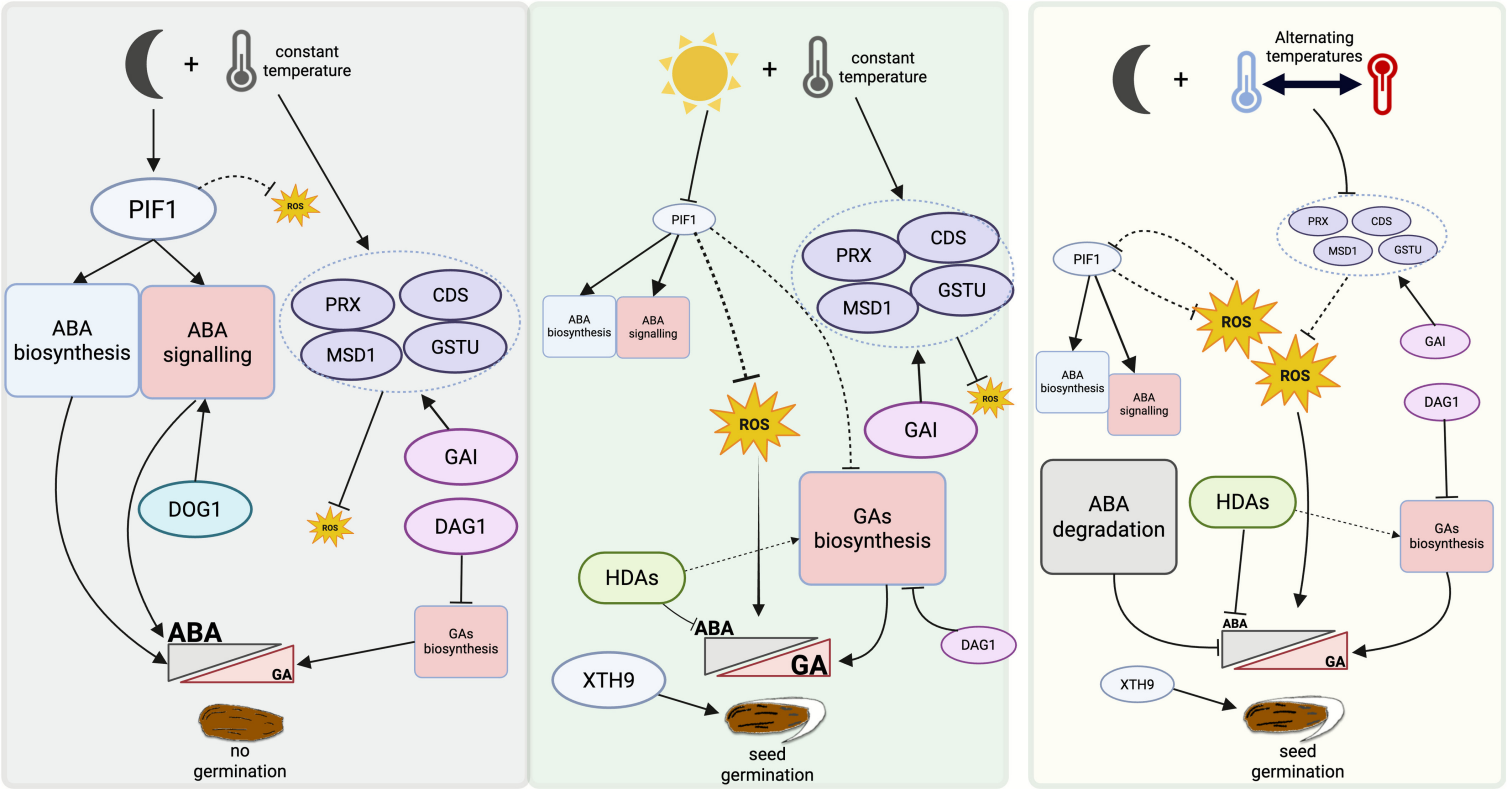
Proposed model for the regulation of seed dormancy release in *D. fullonum* . Left panel (dark, constant temperature): *DfPIF1* and genes related to ROS‐scavenging (*DfPRX, DfCSD, DfMSD1, DfGSTU*) are upregulated. PIF1 promotes ABA biosynthesis and signaling, while antioxidant activity maintains low ROS levels. Together, these mechanisms reinforce the dormant state. Central panel (light, constant temperature): *DfPIF1* is downregulated, whereas antioxidant enzymes and HDAs are upregulated. Reduced ABA biosynthesis/signaling and controlled ROS accumulation favor GA biosynthesis (*DfGA3OX*) and cell wall loosening (*DfXTH9*), leading to dormancy release. Right panel (dark, alternating temperatures): Both *DfPIF1* and antioxidant enzymes are downregulated, resulting in higher ROS accumulation compared to constant darkness. Increased ROS levels promote ABA catabolism, while HDA inhibition of ABA‐responsive genes further contributes to dormancy release. Arrows indicate positive regulation, bars indicate negative regulation; solid lines represent direct interactions, dashed lines represent indirect ones. Upregulated factors are shown with larger icons and labels, while downregulated factors are shown with smaller icons and labels.

With regard to the release of dormancy in the presence of light, we hypothesize that LOM germination at a constant temperature is mainly determined by the phytochrome activity, since germination was stimulated in the dark at a constant temperature only if red light pulses had been applied beforehand, even for a short period. On the other hand, irradiation with a far‐red light pulse, which induces the transition of Pfr to inactive Pr, inhibited germination, regardless of the specific constant temperatures tested. This finding confirms the importance, for this plant, of perceiving its surrounding environment, preferring an open portion of soil (higher proportion of red light) while avoiding the presence of other plants shading (higher proportion of far‐red) that could compete for resources, as already highlighted in other competitive weeds (e.g., Pons 2014). Therefore, in accordance with a large body of literature, we argue that in the dark, with inactive phytochrome, germination inhibition of *D. fullonum* seeds is mainly orchestrated by *DfPIF1* (Figure 10), a major factor that inhibits photomorphogenesis (Oh et al. 2004; Shen et al. 2005; Yang et al. 2020). Its central role in regulating dormancy release in *D. fullonum* seeds in the presence of light is demonstrated by the similar expression patterns of *DfNCED6*, involved in ABA biosynthesis, and *DfABI3, DfPYL4, 8*, and *9*, which interact positively with ABA and its signaling. On the other hand, *DfGA3OX, a gibberellin biosynthetic gene, was downregulated in the dark, while DfDAG1, a direct repressor of GA3OX* (Boccaccini et al. 2016; Lepri et al. 2025), *was upregulated*. These results highlight that the transcriptional control modulating GA and ABA signaling and biosynthesis involved in the light response described in Arabidopsis dormancy (Park et al. 2011; Lepiniec et al. 2018) can also be applied to *D. fullonum* seeds. In addition to this picture, there is also the action of *DfXTH9*, which is upregulated in the presence of light, suggesting its role in the process of weakening cell walls during the completion of germination, as previously described in Arabidopsis (Holdsworth et al. 2008), confirming its role in the phytochrome‐dependent germination process in this species (Wang et al. 2023).

In LOM seeds, the release of dormancy in the dark only in the presence of alternating temperatures allows this accession to be used as a model for the response to alternating temperatures. Under these conditions, the decrease in germination percentages is inversely proportional to irradiation with far‐red light pulses. Moreover, in the dark at alternating temperatures, we observed low levels of *DfPIF1* expression, similar to light conditions, indicating that the regulation of *DfPIF1* plays a role in the release of dormancy in this condition. However, higher levels of ROS compared to darkness at a constant temperature may indicate the presence of a regulatory link between ROS and PIF1 (Figure 9). The relationship between PIF1 and ROS has been previously described in Arabidopsis, where it was described that PIF1 forms a transcription module together with PIF3‐HY5/HYH that acts as a rheostat to precisely regulate the flow of the ROS signaling pathway (Chen et al. 2013). On the other hand, in the present study, the low expression of *DfPIF1* in the dark at alternating temperatures may indicate, for the first time, that this regulation could also occur in the opposite direction through transcriptional inhibition of *DfPIF1* caused by an increase in ROS levels. In line with this, we observed a substantial release of dormancy in the presence of a ROS donor in seeds imbibed in darkness at constant temperatures, showing that higher ROS levels promote dormancy release. This behavior in *D. fullonum* has already been observed with the use of H_2_O_2_ (Huarte et al. 2016). Furthermore, the addition of NAC, a ROS scavenger, even at low concentrations, significantly reduced germination in seeds soaked in the dark at alternating temperatures, while this effect is less marked in light conditions. In accordance with these results, the use of DPI, an inhibitor of Rboh oxidases that is involved in ROS production (Yang et al. 2019), caused a dramatic inhibition of germination at alternating temperatures, even at low concentrations, while it was necessary to add 5 times more DPI to observe an effective reduction in germination in the presence of light. This different behavior observed in the two conditions of light and temperature highlights the direct link between the level of ROS and the release of dormancy in the dark at alternating temperatures. In other words, under these conditions, it appears that dormancy release is mainly dependent on ROS homeostasis. Further evidence of this process is provided by the histochemical analyses, which revealed the presence of both superoxide anions (NBT) and H_2_O_2_ (DAB) in all embryos placed under light or dark conditions at alternating temperatures. The absence of either ROS species in embryos imbibed in darkness at constant temperatures demonstrates that ROS generation only occurs under conditions that stimulate germination. Unfortunately, in this study, it was not possible to identify any gene encoding Rboh oxidases, which play a key role in ROS production (Yang et al. 2019). However, transcriptomic and gene expression data show that ROS scavenger enzymes play an important role in regulating ROS homeostasis in this species. In fact, at alternating temperatures, we observed a downregulation of the expression of genes encoding antioxidant enzymes, such as *DfPRX1*, *DfGSTU*, *DfAPX*, *DfMSD1* and *DfCSD2*, that can lead to an increase in ROS levels (Figure 9). Whereas in darkness at constant temperature, the upregulation of the transcription of antioxidant enzyme genes can maintain ROS levels at low ranges. Superoxide dismutases, such as CSD2 and MSD1, are known to convert superoxide anion radicals to hydrogen peroxide, while APX and PRX1 reduce H_2_O_2_ to water. Their distinct regulation, reflecting circadian rhythm oscillation (Edgar et al. 2012; Jiménez et al. 2021), results in an alteration of ROS homeostasis. Indeed, in the dark at alternating temperatures, *DfPRX1, DfGSTU, DfAPX, DfMSD1*, and *DfCSD2* are transcriptionally repressed, which raises ROS levels and their signaling, promoting dormancy release. We hypothesize that the increase in ROS during this process may also contribute to the downregulation of *DfPIF1*. Nonetheless, we have not established direct evidence of *DfPIF1* regulation by ROS, necessitating future work to clarify this link.

Furthermore, the most significant decrease in ABA concentration occurs in the dark at alternating temperatures, indicating the activation of the ABA catabolic pathway. The future identification of genes directly linked to this process, such as *CYP707A‐like*, may further clarify the basis of this observation; but in this study, it is clear that the ABA/GA balance at alternating temperatures is mainly influenced by a decrease in ABA, which consequently promotes dormancy release. Interestingly, the treatment with MV determined a drastic decrease of ABA, revealing that its catabolism is further promoted by the ROS presence, which has been shown to promote CYP707A activity in barley (Ishibashi et al. 2017). This is in keeping with previous results in another weed, *Cynara cardunculus* , in which dormancy release was proven to be promoted by fluctuating temperatures that turn off ABA synthesis and reduce its signaling, but do not stimulate GA synthesis or signaling (Huarte et al. 2014; Huarte and Benech‐Arnold 2010).

In addition to transcriptional regulation, the inhibitory effect of valproic acid observed in this study indicates that histone deacetylases (HDAs) contribute to dormancy release in *D. fullonum* under certain environmental conditions. Germination was completely suppressed in the dark at alternating temperatures and strongly reduced under light, suggesting that HDAs play a direct role in the regulatory network controlling germination. This hypothesis is consistent with previous findings in *Arabidopsis*, where HDA19 represses seed maturation genes (Tai et al. 2005; Wang et al. 2025) and HDA15 regulates thermoresponsive genes (Shen et al. 2019). Interestingly, *DfHDA19* showed high transcript levels in dormant seeds and low levels during dormancy release, apparently contrasting with its reported role in model species. As this is the first analysis of *HDA19* expression under varying environmental conditions, further work is required to clarify the mechanistic contribution of HDAs to dormancy regulation in *D. fullonum* .

In conclusion, our findings demonstrate that *D. fullonum* is a suitable model plant to explore seed dormancy release in response to environmental signals in invasive and widely distributed species. In the LOM accession, we identified two complementary regulatory routes: (i) a light–PIF1–ABA pathway, in which active phytochrome downregulates ABA signaling and biosynthesis, and (ii) a temperature–ROS pathway, where reduced antioxidant activity increases ROS levels, triggering ABA catabolism and dormancy release even in the dark. Together, these mechanisms expand the ecological window for germination, allowing *D. fullonum* to respond flexibly to heterogeneous environments. Such plasticity, coupled with interpopulation variability, likely contributes to its invasiveness and persistence across diverse disturbed habitats. Future research will further elucidate the basis of this process, which remains largely unexplored in non‐model species but which assumes a strategic importance in understanding the dormancy release in natural populations of invasive weeds.

## Author Contributions

P.F. made germination tests, ROS in situ localization, molecular analyses and revised the manuscript; H.R.H. carried out the hormones treatments, ROS in situ localization, and revised the manuscript; A.P. carried out the transcriptome assemblies and the differential expression analysis; S.S.S. carried out the functional annotation analysis and contributed to the differential expression analysis; V.G. and S.D. made the ABA mass spectrometry analyses and revised the MS; G.D.P. planned and designed the research, drafted and revised the MS; A.C. planned the research and revised the MS. All authors approved the submitted version.

## Disclosure

In this manuscript no generative AI has been employed.

## Conflicts of Interest

The authors declare no conflicts of interest.

## Supporting information


**Figure S1:** Linearity of ABA standard (std) solution in range 2–500 ng/mL.
**Figure S2:** Germination of LOM and PIE seeds collected over three different years.

**Table S1:** Collection sites of *D. fullonum* accessions.

**Table S2:** GLM analysis of germination tests.

**Table S3:** List of primers used for RT‐qPCR.

**Dataset: S1.** List of DEGs identified in this study, including their presence (1) or absence (0) in the pairwise comparison as overlapping genes.

**Dataset: S2.** Full list of enrichment analysis of GO terms with Fisher's test.

**Dataset: S3.** Over‐represented pathways identified with the Plant Reactome Pathways DB and analyzed with Fisher's test.

**File S1:** Alignments of amino acid sequence of the genes analyzed.

## Data Availability

The RNA‐seq data underlying this article are available in the National Center for Biotechnology Information (NCBI) data libraries and can be accessed at https://www.ncbi.nlm.nih.gov/sra/PRJNA1228498 with the BioProject ID number PRJNA1228498.
