# Supplementary material for: Light and Alternating Temperatures Release Seed Dormancy in the Invasive Dipsacus fullonum L. Through ROS Homeostasis and ABA Regulation
Source: Physiol Plant. 2025 Nov 19;177(6):e70642. doi: 10.1111/ppl.70642 (PMC12628119; doi:10.1111/ppl.70642)
Supplement: Supplementary file 1 — Figure S1: Linearity of ABA standard (std) solution in range 2–500 ng/mL. Figure S2: Germination of LOM and PIE seeds collected over three different years. [file PPL-177-e70642-s006.docx]

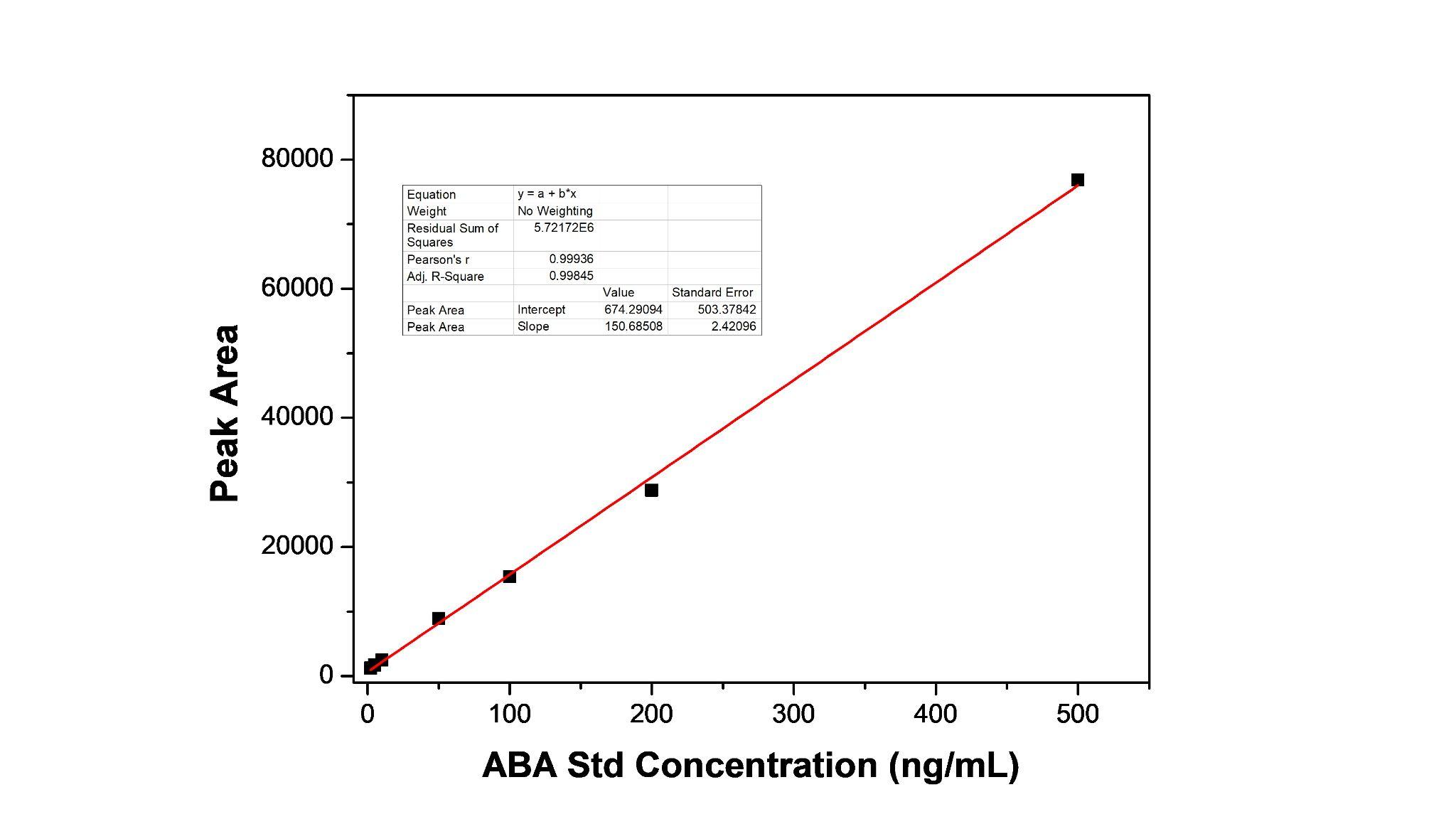


**Figure S1.** Linearity of ABA standard (std) solution in range 2-500 ng/mL.



**Figure S2.** Germination of LOM and PIE seeds collected in three different years. Seeds were imbibed in light (yellow bars), dark (orange bars), alternate temperatures (dark blue), or alternate temperatures plus light (light blue bars).
