# Supplementary material for: Light and Alternating Temperatures Release Seed Dormancy in the Invasive Dipsacus fullonum L. Through ROS Homeostasis and ABA Regulation
Source: Physiol Plant. 2025 Nov 19;177(6):e70642. doi: 10.1111/ppl.70642 (PMC12628119; doi:10.1111/ppl.70642)
Supplement: Supplementary file 2 — Table S1: Collection sites of D. fullonum accessions. [file PPL-177-e70642-s005.docx]

**Table S1.** Collection sites of *D. fullonum* accessions.

| **Population** | **ID** | **Location** | **GPS coordinates** | |
| --- | --- | --- | --- | --- |
|  |  |  | **Latitude** | **Altitude** |
| Simeto | SIM | Passo Cavaliere, Catania | 37° 26’ 01.4” | 15° 00' 03.7” |
| Etna | ETN | Pantano, Castiglione di Sicilia | 37° 51’ 36” | 15° 06’ 39” |
| Pietraperzia | PIE | Piazza Armerina | 37° 26’ 30.51” | 14° 11’ 30.41” |
| Lomas | LOM | Lomas de Zamora | -34° 46’ 27” | -58° 27’ 19” |
| La Plata | LAP | Reserva Ecológica del Parque Pereyra | -34° 50’ 42” | -58° 07’ 00” |
| Bahía | BAH | Bahía Blanca | -38° 41’ 45” | -62° 12’ 56” |
