# Supplementary material for: Light and Alternating Temperatures Release Seed Dormancy in the Invasive Dipsacus fullonum L. Through ROS Homeostasis and ABA Regulation
Source: Physiol Plant. 2025 Nov 19;177(6):e70642. doi: 10.1111/ppl.70642 (PMC12628119; doi:10.1111/ppl.70642)
Supplement: Supplementary file 3 — Table S2: GLM analysis of germination tests. [file PPL-177-e70642-s008.docx]

**Supplemental Table 2.** GLM analysis of germination tests.

| **PIE** | **Temperature (°C)** | **Estimate** | **SE** | ***z*-ratio** | ***p*-value** |
| --- | --- | --- | --- | --- | --- |
| Dark - light | 5 | -0.108 | 0.051 | -2.100 | 0.035 |
|  | 10 | -0.683 | 0.045 | -15.183 | 4.55E-52 |
|  | 15 | -0.116 | 0.031 | -3.726 | 0.00019 |
|  | 15/5 | -0.225 | 0.043 | -5.209 | 1.89E-07 |
|  | 20 | -0.025 | 0.0183 | -1.361 | 0.173 |
|  | 20/10 | -0.008 | 0.0143 | -0.581 | 0.560 |
|  | 25 | -0.041 | 0.024 | -1.709 | 0.087 |
|  | 25/15 | -0.008 | 0.026 | -0.308 | 0.757 |
|  | 30 | -0.066 | 0.025 | -2.620 | 0.008 |
|  | 30/20 | 0.008 | 0.021 | 0.383 | 0.701 |
|  | 35 | -1.4E-17 | 0.011 | -1.2E-15 | 1 |
| Alternating - constant  (dark) | 10 | 0.475 | 0.056 | 8.362 | 6.133E-17 |
|  | 15 | 0.108 | 0.041 | 3.346 | 0.000818 |
|  | 20 | -0.016 | 0.036 | -0.646 | 0.517 |
|  | 25 | 0.033 | 0.046 | 1.296 | 0.194 |
| Alternating - constant  (light) | 10 | 0.016 | 0.025 | 0.646 | 0.517 |
|  | 15 | 1.11E-16 | 0.011 | 9.46E-15 | 1 |
|  | 20 | -0.033 | 0.020 | -1.663 | 0.096 |
|  | 25 | -0.016 | 0.0201 | -0.828 | 0.407 |

| **LAP** | **Temperature (°C)** | **Estimate** | **SE** | ***z*-ratio** | ***p*-value** |
| --- | --- | --- | --- | --- | --- |
| Dark - light | 5 | 0.008 | 0.021 | 0.383 | 0.701 |
|  | 10 | -0.075 | 0.026 | -2.823 | 0.004 |
|  | 15 | -0.216 | 0.039 | -5.553 | 2.7961E-08 |
|  | 15/5 | 1.1102E-16 | 0.011 | 9.4601E-15 | 1 |
|  | 20 | -0.316 | 0.043 | -7.270 | 3.5792E-13 |
|  | 20/10 | 1.1102E-16 | 0.011 | 9.4601E-15 | 1 |
|  | 25 | -0.275 | 0.041 | -6.553 | 5.6316E-11 |
|  | 25/15 | 1.1102E-16 | 0.011 | 9.4601E-15 | 1 |
|  | 30 | -0.35 | 0.044 | -7.855 | 3.9709E-15 |
|  | 30/20 | 0 | 0.011 | 0 | 1 |
|  | 35 | 0.016 | 0.016 | 1.010 | 0.312 |
| Alternating - constant  (dark) | 10 | 0.075 | 0.026 | 2.823 | 0.004 |
|  | 15 | 0.216 | 0.039 | 5.55 | 2.7961E-08 |
|  | 20 | 0.316 | 0.043 | 7.270 | 3.5792E-13 |
|  | 25 | 0.275 | 0.041 | 6.553 | 5.6316E-11 |
| Alternating - constant  (light) | 10 | -1.1102E-16 | 0.011 | -9.4601E-15 | 1 |
|  | 15 | -2.2204E-16 | 0.011 | -1.892E-14 | 1 |
|  | 20 | 1.1102E-16 | 0.011 | 9.4601E-15 | 1 |
|  | 25 | -2.2204E-16 | 0.011 | -1.892E-14 | 1 |

| **ETN** | **Temperature (°C)** | **Estimate** | **SE** | ***z*-ratio** | ***p*-value** |
| --- | --- | --- | --- | --- | --- |
| Dark - light | 5 | -0.166 | 0.054 | -3.074 | 0.002 |
|  | 10 | -0.316 | 0.058 | -5.389 | 7.0731E-08 |
|  | 15 | -0.258 | 0.049 | -5.240 | 1.6007E-07 |
|  | 15/5 | -0.083 | 0.044 | -1.864 | 0.062 |
|  | 20 | -0.191 | 0.044 | -4.264 | 2.0057E-05 |
|  | 20/10 | -0.125 | 0.041 | -3.026 | 0.002 |
|  | 25 | -0.058 | 0.044 | -1.316 | 0.187 |
|  | 25/15 | -1.1102E-16 | 0.028 | -3.9458E-15 | 1 |
|  | 30 | -0.466 | 0.048 | -9.622 | 6.4058E-22 |
|  | 30/20 | -0.775 | 0.039 | -19.624 | 9.5888E-86 |
|  | 35 | -2.949E-17 | 0.011 | -2.5128E-15 | 1 |
| Alternating - constant  (dark) | 10 | 0.341 | 0.057 | 5.924 | 3.1319E-09 |
|  | 15 | 0.15 | 0.055 | 2.694 | 0.007 |
|  | 20 | 0.2 | 0.044 | 4.519 | 6.1997E-06 |
|  | 25 | -0.641 | 0.049 | -12.967 | 1.8677E-38 |
| Alternating - constant  (light) | 10 | 0.108 | 0.046 | 2.350 | 0.018 |
|  | 15 | 0.016 | 0.032 | 0.517 | 0.604 |
|  | 20 | 0.008 | 0.029 | 0.285 | 0.775 |
|  | 25 | 0.075 | 0.032 | 2.289 | 0.022 |

| **BAH** | **Temperature (°C)** | **Estimate** | **SE** | ***z*-ratio** | ***p*-value** |
| --- | --- | --- | --- | --- | --- |
| Dark - light | 5 | -0.675 | 0.045 | -14.881 | 4.3262E-50 |
|  | 10 | -0.325 | 0.043 | -7.415 | 1.2099E-13 |
|  | 15 | -0.733 | 0.040 | -17.969 | 3.3978E-72 |
|  | 15/5 | -0.183 | 0.036 | -4.971 | 6.6468E-07 |
|  | 20 | -0.983 | 0.011 | -83.788 | 0 |
|  | 20/10 | -0.1 | 0.029 | -3.382 | 0.0007 |
|  | 25 | -0.983 | 0.011 | -83.788 | 0 |
|  | 25/15 | 0 | 0.011 | 0 | 1 |
|  | 30 | -0.966 | 0.016 | -58.613 | 0 |
|  | 30/20 | -0.166 | 0.035 | -4.673 | 2.9666E-06 |
|  | 35 | -6.4185E-17 | 0.011 | -5.4691E-15 | 1 |
| Alternating - constant  (dark) | 10 | 0.141 | 0.056 | 2.526 | 0.011 |
|  | 15 | 0.633 | 0.049 | 12.923 | 3.317E-38 |
|  | 20 | 0.983 | 0.011 | 83.788 | 0 |
|  | 25 | 0.816 | 0.035 | 22.898 | 4.833E-116 |
| Alternating - constant  (light) | 10 | 1.1102E-16 | 0.011 | 9.4601E-15 | 1 |
|  | 15 | 0 | 0.011 | 0 | 1 |
|  | 20 | -1.1102E-16 | 0.011 | -9.4601E-15 | 1 |
|  | 25 | -1.1102E-16 | 0.011 | -9.4601E-15 | 1 |

| **SIM** | **Temperature (°C)** | **Estimate** | **SE** | ***z*-ratio** | ***p*-value** |
| --- | --- | --- | --- | --- | --- |
| Dark - light | 5 | -0.033 | 0.020 | -1.663 | 0.096 |
|  | 10 | -0.891 | 0.028 | -30.770 | 6.418E-208 |
|  | 15 | -0.883 | 0.030 | -29.212 | 1.348E-187 |
|  | 15/5 | -0.65 | 0.044 | -14.576 | 3.9527E-48 |
|  | 20 | -0.425 | 0.050 | -8.434 | 3.3165E-17 |
|  | 20/10 | -0.558 | 0.048 | -11.560 | 6.5685E-31 |
|  | 25 | -0.591 | 0.049 | -11.917 | 9.5629E-33 |
|  | 25/15 | -0.408 | 0.047 | -8.609 | 7.3361E-18 |
|  | 30 | -0.708 | 0.044 | -15.868 | 1.044E-56 |
|  | 30/20 | -0.45 | 0.046 | -9.732 | 2.1858E-22 |
|  | 35 | -5.8981E-17 | 0.011 | -5.0257E-15 | 1 |
| Alternating - constant  (dark) | 10 | 0.308 | 0.045 | 6.801 | 1.034E-11 |
|  | 15 | 0.333 | 0.050 | 6.642 | 3.0922E-11 |
|  | 20 | 0.05 | 0.064 | 0.778 | 0.436 |
|  | 25 | 0.216 | 0.062 | 3.470 | 0.0005 |
| Alternating - constant  (light) | 10 | 0.066 | 0.027 | 2.397 | 0.016 |
|  | 15 | 0.008 | 0.026 | 0.308 | 0.757 |
|  | 20 | 0.033 | 0.025 | 1.296 | 0.194 |
|  | 25 | 0.075 | 0.026 | 2.823 | 0.004 |

| **LOM** | **Temperature (°C)** | **Estimate** | **SE** | ***z*-ratio** | ***p*-value** |
| --- | --- | --- | --- | --- | --- |
| Dark - light | 5 | -0.31667 | 0.055 | -5.757 | 8.56E-09 |
|  | 10 | -0.60833 | 0.047 | -12.886 | 5.38E-38 |
|  | 15 | -0.875 | 0.030 | -28.515 | 7.5E-179 |
|  | 15/5 | -0.175 | 0.036 | -4.823 | 1.41E-06 |
|  | 20 | -0.975 | 0.014 | -68.024 | 0 |
|  | 20/10 | -0.116 | 0.031 | -3.726 | 0.00019 |
|  | 25 | -0.925 | 0.024 | -38.166 | 0 |
|  | 25/15 | -0.175 | 0.036 | -4.823 | 1.41E-06 |
|  | 30 | -0.883 | 0.029 | -29.881 | 3.4E-196 |
|  | 30/20 | -0.55 | 0.046 | -11.934 | 7.83E-33 |
|  | 35 | -1.4E-17 | 0.011 | -1.2E-15 | 1 |
| Alternating - constant  (dark) | 10 | 0.466 | 0.056 | 8.323 | 8.55E-17 |
|  | 15 | 0.766 | 0.041 | 18.505 | 1.88E-76 |
|  | 20 | 0.808 | 0.036 | 22.277 | 6.1E-110 |
|  | 25 | 0.433 | 0.046 | 9.402 | 5.31E-21 |
| Alternating - constant  (light) | 10 | 0.033 | 0.020 | 1.663 | 0.096 |
|  | 15 | 0.008 | 0.014 | 0.581 | 0.560 |
|  | 20 | 0.008 | 0.014 | 0.581 | 0.560 |
|  | 25 | 0.058 | 0.024 | 2.406 | 0.016 |
