# Supplementary material for: Light and Alternating Temperatures Release Seed Dormancy in the Invasive Dipsacus fullonum L. Through ROS Homeostasis and ABA Regulation
Source: Physiol Plant. 2025 Nov 19;177(6):e70642. doi: 10.1111/ppl.70642 (PMC12628119; doi:10.1111/ppl.70642)
Supplement: Supplementary file 4 — Table S3: List of primers used for RT‐qPCR. [file PPL-177-e70642-s004.docx]

**Table S3.** List of primers used for RT-qPCR

| Gene | Forward primer (5’-3’) | Reverse primer (5’-3’) | T_a_ (°C) |
| --- | --- | --- | --- |
| *ABI3* | GCAGAAACCCATCTTCCACC | TTCGGCCAAAACCTGAGACT | 55 |
| *ACT4* | GAGGACATTCAGCCACTCGT | TGCCAACCATCACACCAGTA | 53 |
| *APPR1* | ACGAGGTCTCTGTTGTTGTCA | CGCCTCCTCCTCCACATG | 58 |
| *APX* | CAGATCTTGACCGGAAAGCG | AAGGAATCGTTCTTGCCTGTC | 58 |
| *COP1* | AGCTTTTATGTTGCGGCTGG | TTTTCTTGTGCCCGCTGAAA | 57 |
| *COP9* | ACGGCATCCTCAGAACAGTA | GTTGGAGTGTATAGTTTGTAGCA | 59 |
| *CSD2* | ACAAGAACGAGGCTGTGTCA | GCCTCACGTTCTTGTACTGC | 61 |
| *DAG1* | AGAACAAGGATTATGGAGGTGAT | GCCATCTCTCTGTCGCACAT | 57 |
| *DOG1* | TTGAATGGGGTACGTACGGG | TGAAATTCTCCTCATCAACAGTT | 56 |
| *EF1* | TGTGCTGTGCTCATTATCGA | TGGCATCCATCTTGTTACAAC | 56 |
| *EM6* | CTCCCTTGCTCCGACCTTC | TCTCAGCAGCAAAGACCGTA | 54 |
| *GA3ox1* | GAACATGCACGCCAACTTTG | CCCATTTAACATCTTCAGTGGCT | 55 |
| *GAI* | AGTTCTTCAATGGCAGCTCC | AGCGACATCAGACATCTCCG | 57 |
| *GSTU* | TAAGAAGATCTATGATGCTGGGA | CAAGCACTCCTTCCAACACC | 54 |
| *HDA19* | TGGATCTTGTGACATTGCGA | GTACAAAACACGCTCGT | 56 |
| *MSD1* | CATGTCAACTGGGCCACATT | TTTCCAAGATCGCCAGCATG | 59 |
| *NCED6* | AATAGGAAAATGATCAGGACTG | GAGGAAGATGAAGGTTATATC | 56 |
| *NIA1* | CGCCGTGTCAAGAGGAGATA | TCCACCGCCTGAATAAGCAT | 61 |
| *PER1* | TCCTCTTCTCTCACCCAGGT | AACCTTGTGCCCCGAGTTAT | 58 |
| *PIF1* | GATCTCGACGCCTCCTCTC | CAGAGTGTCACAGTGAGGGT | 58 |
| *PYL4* | TCGAGATTCTAGACGACGAGC | AACTGTCCCATTCCCCGTAG | 58 |
| *PYL8* | TGGATGTTAAGTCGGGCCTT | AGAGTAGTTCCTAAGCCTGTGA | 56 |
| *PYL9* | GTGATCACCGTCTAAAGAATTAT | TCGACTTTGGTTCATTGCCA | 55 |
| *TPL* | TGTAAAGCAGTGGGTCCCA | TCTAAGGCGGAGGTGTGAAG | 58 |
| *XTH9* | GAACCAGCGTCAAGTTGTATTTT | CCCAGTCATCCGCATTCCAT | 58 |
