# Supplementary material for: Light and Alternating Temperatures Release Seed Dormancy in the Invasive Dipsacus fullonum L. Through ROS Homeostasis and ABA Regulation
Source: Physiol Plant. 2025 Nov 19;177(6):e70642. doi: 10.1111/ppl.70642 (PMC12628119; doi:10.1111/ppl.70642)

**Supplemental File 1. Alignments of amino acid sequence of the genes analysed.** For each sequence the putative name and accession number of the characteristic domain is reported. The sequence corresponding to the domain of *D. fullonum* is highlighted in yellow or blue.

# DfABI3

Domain: pfam02362

D.fullonum_ABI3 MGTYQVQDEDLHAAAQV-EQENDLVADMLKGYDNDEIPTIDPGEFLEPKAAIETEIWLGE

A.thaliana_ABI3 M-------KSLHVAANAGDLAEDCGIL---GGDADDTVLMDGIDEV------GREIWLDD

C.florida_ABI3 MDKESFG-EGVHASVEN-KLEDKVGVD-----DLANTPLFDAMEEEREMGVGGTEIWSLD

J.regia_ABI3 MKGEELQSEDLQA-----------GVR-----NYGNPTGFDAMKEEEALSVDERQIWLSN

C.illinoinensis_ABI3 MEGEELQSEDLQA-----------GVR-----KYGKPTGSDAMKEEEALSVDERQIWLSN

* :.::. . . * . :** :

D.fullonum_ABI3 G--------TQGDNILDDVDVNGAASSIFYNEDFPVLPDFPCMSSSSSSSSIPAPCKA--

A.thaliana_ABI3 HGGDNNHVHGHQDDDLIVH----HDPSIFYG-DLPTLPDFPCMSSSSSSSTSPAPVNAIV

C.florida_ABI3 ----------RQPEDLLDV----NERSIFYN-DFPPLPDFPCMSSSSSSSSSPAKANTM-

J.regia_ABI3 ----------EQDHLLADV----HDISLFYT-DFPPLPDFPCMSSSSSSSSIPAPVKAIT

C.illinoinensis_ABI3 ----------EQDHLLADV----HDISLFYT-DFPPLPDFPCMSSSSSSSSIPAPVKAIT

. . * *:** *:* **************: ** ::

D.fullonum_ABI3 -PVSPTA--SCSSSSAASRALMRSDQSQDGP-----------------------------

A.thaliana_ABI3 SSASSSSAASSSTSSAASWAILRSDGEDPTP-NQNQYASG---NCDDSSGALQSTASMEI

C.florida_ABI3 -TSSASS--SASSSSVASWAVLRSETERQGE-RKNHH-----DQVGATPTALSSTASMEV

J.regia_ABI3 CSSSSSS--ASSSSSAASWALLKSDAEEDVEIKKNHHHNGLHDPVDAPPGALSSTASMEI

C.illinoinensis_ABI3 CSSSSSS--ASSSSSAASWALLKSDAEEDVE-KKNHHHNGLHDPVDALPGALSSTASMEI

. *.:: :.*:**.** *:::*: .

D.fullonum_ABI3 ----------AGGTVECMDGVENLEYMDLIDVSDLWDPSSIFEEEENM-SQVQNRQVVQE

A.thaliana_ABI3 PLDSSQGFGCGEGGGDCIDMMETFGYMDLLDSNEFFDTSAIFSQDDDT-QNPNLMDQTL-

C.florida_ABI3 -IPPTDVI----GNVDCMGVVDNFEYMDLIDGDDAWDPSTFLQTD-NP-QEFTQEESTQ-

J.regia_ABI3 PQPPDHGL----EDVDCMDVMETLGYMDLIESNDFFDPSCIFQSE-NPLEDFQQEQMSQV

C.illinoinensis_ABI3 PQPPDHGL----EDVDCMDVMETLGYMDLIESNDFFDPSCIFQSENNPLEDFQQEQMSLE

:*:. ::.: ****:: .: :*.* ::. : : .: :

D.fullonum_ABI3 EHR----------------------------GAGKHCDELGMMFFEWLKNNRNHITAEEM

A.thaliana_ABI3 -----ERQEDQVVVPMMENNSG--GDMQMMNSSLEQDDDLAAVFLEWLKNNKETVSAEDL

C.florida_ABI3 -----QYQTENNEQFVIQSNNGLNGGVAEKDGGGRPSEELAVVFLEWLKSNKEYISAEDM

J.regia_ABI3 EHAL-QHAHREHDQLMLQSNNDKETGHEEKD-----PDDMAAVFLEWLRTNKETFSAEDL

C.illinoinensis_ABI3 EHAMQQHAHREHDQLMLQGNNDKETGHEEKD-----PDDMATVFLEWLRTNKETVSAEDL

:::. :*:***:.*:: .:**::

D.fullonum_ABI3 RSIKLKRSTVESALKRLGTTMEGKKQLLKLILQWVEQYQLQKKRNPNPVSQFPSQ---YV

A.thaliana_ABI3 RKVKIKKATIESAARRLGGGKEAMKQLLKLILEWVQTNHLQRRRTTTTTTNLSYQQSFQQ

C.florida_ABI3 RSIKIRRSTIECASKRLGTSKEGQKQLLKLILEWVEQYQLNKKKNGEPTTPFPYQ---YE

J.regia_ABI3 RSVRIKKATIESAARRLGGGKEAMKQLLKLVLEWVQMNHLQKRRNKEAPTHFPEQ---YQ

C.illinoinensis_ABI3 RSVRIKKATIESAARRLGGGKEAMKQLLKLVLEWVQTNHLQKRRNKESPTHFPEQ---YQ

*.::::::*:*.* :*** *. ******:*:**: :*::::. . : :. *

D.fullonum_ABI3 DPLQNPN----PNPNLAANSILINPNTPFH-PPT-WIPTP--VQPYTADPGALLAPGSVP

A.thaliana_ABI3 DPFQNPN----PN---NNNLIPPSDQTCFS-PST-WVPPPPQQQAFVSDPG---------

C.florida_ABI3 EPFPNPSLSPTSNPNLTCNSVPPDPNACLS-PSP-WMPPP--QTPYIADPATVVTT----

J.regia_ABI3 DPFQNPN----PNVNLNSESVTPESNPCFATQSSTWIPQP----PCVAEPTAVLAP----

C.illinoinensis_ABI3 DPFQNSN----PNADLNSESVTPEPNPCFATQSS-WIPQT----PYVAEPTAVLGP----

:*: *.. .* : : . :. : .. *:* . . ::*

D.fullonum_ABI3 TPVQVPPGF-YVTNSYPDVAN-FKPTPTCQYPPLPQRVEY-QMID-GQTWQNSKYVIGSQ

A.thaliana_ABI3 --------FGYMPAP-----------------NYPPQPEFLPLLESPPSWPPPPQSGPMP

C.florida_ABI3 -PQVF-PMVGYMGDPYSNNGTTSQLNQTANVHPFPASTEY-QIMDSAQPWPPTQIAMASP

J.regia_ABI3 -PAAYPPMVGYMGDPYTHGA--SNLNSHS---PYPAPTEY-HMLDSSHSWSASQFSLASH

C.illinoinensis_ABI3 -PAAYPPMVGYMGDPYTHGA--SNLNSHS---PYPAPTEY-HMLDSSHSWSPSQFSLASH

. *: . * *: ::: .* .

D.fullonum_ABI3 YTFAENINMNVVPDTM-NPQSAGFYGDQYRYPCHQVVGENG----ERSVRLGSLSATKEA

A.thaliana_ABI3 HQQFPMPP-TSQYNQFGDPTGFNGY-NMNPYQ--YPYVPAGQMRDQRLLRLCS-SATKEA

C.florida_ABI3 YNPYQEID-NLSPAQL-HPQAVTGYGNQYPYP--YNYQGSG----EKLVKLGP-SATKEA

J.regia_ABI3 YNAFPE-N-NLHQ----APQPFAGYGNQHPYQ--QYFNGHG----ERLLRLGS-SATKEA

C.illinoinensis_ABI3 YNAFPE-N-NLHQ----APQPFAGYGNQYPYQ--QYFNGHG----ERLLRLGS-SATKEA

: . * * : * * :: ::* . ******

D.fullonum_ABI3 RKKRMARQRKSFFHH-HHHSRHQHHTSNNHQNQV-VADQNLRLGA--EHC---AINGQSN

A.thaliana_ABI3 RKKRMARQRRFLSHH-HRHNNNNNNNNNNQQNQTQIGETCAAVAPQLN-----PVATTAT

C.florida_ABI3 RKKRMARQKRIFAHH-RHHNHQNNPNPPQTQNQS--ADQHARVGSTHDNC-T-NTTVQAN

J.regia_ABI3 RKKRMARQRRFLSHHRHNHNHHNQQN--QHQSQN--ADQHARLGG--DNCSTTAVAAQAN

C.illinoinensis_ABI3 RKKRMARQRRFLSHHRHHHNHHNQQN--QQQSQN--VDQHARLGG--DNC-TAAAATQAN

********:: : ** :.*..::: . : *.* : :. : :.

D.fullonum_ABI3 AGAWVYVPCGSGAAPGVSTVRMIPPAIAPPAAPPPEAQKRTNVQNYQ-RQAAADRRQGWK

A.thaliana_ABI3 GGTWMYWPNVPAVP-------PQLPP--VMETQLPTMDRAGSASAMPRQQVVPDRRQGWK

C.florida_ABI3 QGNWVYWPQVADTS--ASTGGAMMPPVDVPSQPYPVDQPPMQVQNYQ-RQAMVDRRQGWK

J.regia_ABI3 PGNWVYWPSTTGGV--AS-GSPVMPA--EMALGIPADRMAMHGQNFP-VRVASDRRQGWK

C.illinoinensis_ABI3 PGNWVYWPSTTGGV--AS-GSPVMPA--EMALGIPEDPTAMHEQNFP-GRVASDRRQGWK

* *:* * . *. * . :. *******

D.fullonum_ABI3 QEKNLKFLLQKVLKQSDVGNLGRIVLPKKEAETHLPPLETRDGISITIEDIGTSRLWNMR

A.thaliana_ABI3 PEKNLRFLLQKVLKQSDVGNLGRIVLPKKEAETHLPELEARDGISLAMEDIGTSRVWNMR

C.florida_ABI3 PEKNLKFLLQKVLKQSDVGNLGRIVLPKKEAETHLPELEARDGITIPMEDIGTSCVWNMR

J.regia_ABI3 PEKNLRFLLQKVLKQSDVGNLGRIVLPKKEAETHLPELEARDGISIAMEDIGTSRVWNMR

C.illinoinensis_ABI3 PEKNLRFLLQKVLKQSDVGNLGRIVLPKKEAETHLPELEARDGISIAMEDIGTSRVWNMR

****:****************************** **:****::.:****** :****

D.fullonum_ABI3 YSLRFWPNNKSR--------------GMK---FKVV------------------------

A.thaliana_ABI3 Y--RFWPNNKSRMYLLENTGDFVKTNGLQEGDFIVIYSDVKCGKYLIRGVKVRQPSGQKP

C.florida_ABI3 Y--RFWPNNKSRMYLLENTGDFVRANGLQEGDFIVIYSDVKCGKYLIRGVKVRQP-GQKS

J.regia_ABI3 Y--RYWPNNKSRMYLLENTGDFVRANGLQEGDFIVIYSDVKCGKYMIRGVKVRQP-GPKS

C.illinoinensis_ABI3 Y--RYWPNNKSRMYLLENTGDFVRANGLQEGDFIVIYSDVKCGKYMIRGVKVRQP-GPKS

* *:******* *:: * *:

D.fullonum_ABI3 ------------------------------------------------------------

A.thaliana_ABI3 EAPPSSAATKRQNK-SQRNINNNSPSANVVV------------------------ASPTS

C.florida_ABI3 EGRKSGKGHK--------NLRTANPALGTVSIHELAWFQISAIIGTYAEFGNDKNLSPSL

J.regia_ABI3 ETKRPGKSQKNQHASTSTTTTTPAGAEDAIS------------------------SSPTA

C.illinoinensis_ABI3 ESKRPGKSQKLQHA-TSTTTTAPAGAEGAIS------------------------SSPTA

D.fullonum_ABI3 ------

A.thaliana_ABI3 QTV--K

C.florida_ABI3 SQYAFV

J.regia_ABI3 RKK---

C.illinoinensis_ABI3 RKK--Q


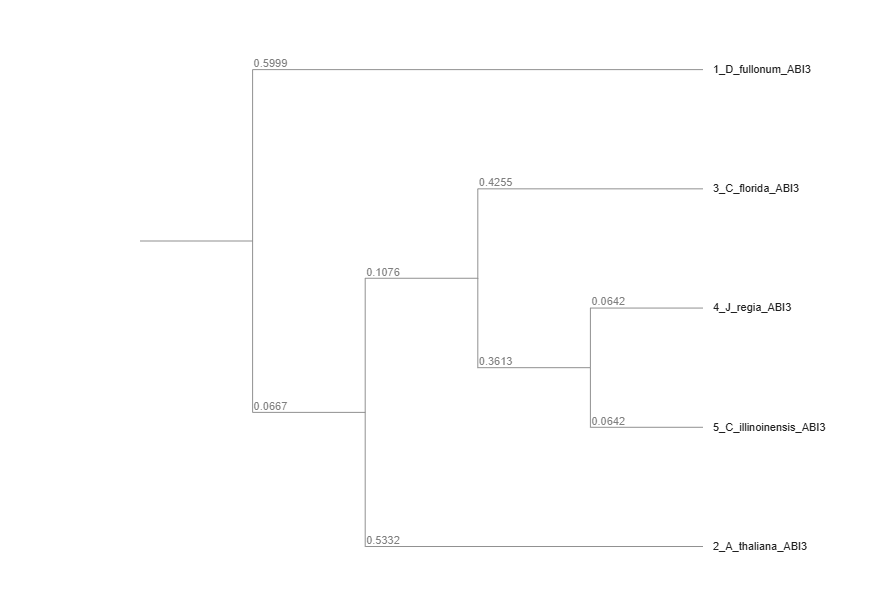


# DfAPRR1

Domain: cd17582

A.paniculata_APRR1 -------------------MEKSGDGFVDRSKVRILLCDNDEKSSAEVFTLLCKCSYQVT

O.europaea.europaea_APRR1 -MEK-------------SEIGKSGDGFIDRSKVRILVCDNDAKTSEEVLSLLCKCSYQVT

D.fullonum_APRR1 -MDRRSEIVLGKESGGVRNISKSCDGFIDRSKVRILLCDNDSKSSEEVFKLLCGCSYQVT

I.tiloba_APRR1 MMEK-------------NEIVKTGDGFIDRSKVRILLCDNDSKSSEEVFTLLCKCSYQVT

I.batatas_APRR1 MMEK-------------NEIVKTGDGFIDRSKVRILLCDNDSKSSEEVFTLLCKCSYQVT

: *: ***:********:**** *:* **:.*** ******

A.paniculata_APRR1 SVKSPRQVIDALNAEGPDIDIILSEVDLPMSKGLKMLKYIMRDKELRRIPVIMMSAQDEV

O.europaea.europaea_APRR1 SARSPRQVIDALNAEGPDIDIILSEVDLPMSKGLKMLKYITRDKELRRIPVIMMSAQDEV

D.fullonum_APRR1 SAKSPRQVIDALNAEGPDIDIILSEVDLPMTKGLKMLKYIMRDKGLRRIPVIMMSAQDEV

I.tiloba_APRR1 SVRSPRQVIDALNAEGPDIDIILSEVDLPMSKGLKLLKYIMRDKELRRIPVIMMSSQDEV

I.batatas_APRR1 SVRSPRQVIDALNAEGPDIDIILSEVDLPMSKGLKLLKYIMRDKELRRIPVIMMSSQDEV

*.:***************************:****:**** *** **********:****

A.paniculata_APRR1 SIVVKCLKFGAADYLVKPLRTNELLNLWTHMWRRRRMLGLAEKNIVNYDFDPLVSDPSDA

O.europaea.europaea_APRR1 SIVVKCLKFGAADYLVKPLRTNELLNLWTHMWRRRRMLGLEEKNIVNYDFDMVVSDPSDA

D.fullonum_APRR1 SVVVKCLRLGAADYLVKPLRTNELLNLWTHMWRRRRMLGLEEKKMLNYDFF---------

I.tiloba_APRR1 SVVVKCLKLGAADYLVKPLRTNELLNLWTHMWRRRRMLGLAEKNILNYDFDLVVSDPSDA

I.batatas_APRR1 SVVVKCLKLGAADYLVKPLRTNELLNLWTHMWRRRRMLGLAEKNILNYDFDLVVSDPSDA

*:*****::******************************* **:::****

A.paniculata_APRR1 NTNSTTLFSDDTDDKSRKSTNPDACASTHPENEHLACQNNVATAAVSVETASETPSECQP

O.europaea.europaea_APRR1 NTNSTTLFSDDTDDKSRKSINPVTCVLTHEEDE-----TNAAATPSPIETPTGALSERQP

D.fullonum_APRR1 ------------------------------------------------------------

I.tiloba_APRR1 NTNSTTLFSDDTDEKSRKSINLETGPSTQQEDE-----TNAITNAASPETLVIGSFECLP

I.batatas_APRR1 NTNSTTLFSDDTDEKSRKSINLETGPSTQQEDE-----TNAITNAASPETLVIGSFECLP

A.paniculata_APRR1 DL-----RIRDHGSPFPKKSGLRIGESSAFFTYIKSSMHENAPRLEKTVEE---------

O.europaea.europaea_APRR1 DVPVISDRQTGKISPFPKKSGLKIGESSAFFTYIKSSTPQCNNQGDASVRETLPRNLRIK

D.fullonum_APRR1 ------------------------------------------------------------

I.tiloba_APRR1 DVPGSSDRKTGKICSFPKKSELKIGESSAFFTYVKSSMPKSNDQ--VTVRENVTYHSRIN

I.batatas_APRR1 DVPGSSDRKTGKICSFPKKSELKIGESSAFFTYVKSSMPKSNDQ--VTVRENVTYHSRIN

A.paniculata_APRR1 --------HMDIRGKK--DQDFRESQSQPDEHPSNNTVPDSSFSMERSSTPQISNPAEIS

O.europaea.europaea_APRR1 EKLTTMKGRVGADFLGHDNRDSLENHFQEDEYPSSNSIPD-SFSVERSCTPPIS--LETS

D.fullonum_APRR1 ------------------------------------------------------------

I.tiloba_APRR1 E-----GGNVDIESKERANGDAIENHSQGDGYPSSNSIPD-SLSMERSCTPPLS--MEFP

I.batatas_APRR1 E-----GGNVDIESKERANGDAIENHSQGDGYPSSNSIPD-SLSMERSCTPPLS--MEFP

A.paniculata_APRR1 QQHLSGEQFSHPHLHESPSNAPNH--------------YPYPFYMPSVMNQVTMPPASMY

O.europaea.europaea_APRR1 QQRNSNEEFSQVHMH--PRNESPHYGVGFLANPAQ------PYYMSGVMNQSMMSSAAVY

D.fullonum_APRR1 ------------------------------------------------------------

I.tiloba_APRR1 QQR--MEEFSKVHMH--PTNESHHDISGY--HAHAHAAYP-PYYIPRIMNQVMMPSSQMY

I.batatas_APRR1 QQR--MEEFSKVHMH--PTNESHHDISGYHAHAHAHAAYP-PYYIPRIMNQVMMPSSQMY

A.paniculata_APRR1 ---------HNH----------HIPQCPPHIPAMASYPYYPYGICLQPGQMPVPPPHPWP

O.europaea.europaea_APRR1 RKHTQPQDLHYHANSSLLPQYNHIPQGGRHVPGMTSYPYYPFGICLQHGEMPT-TTHAWP

D.fullonum_APRR1 ------------------------------------------------------------

I.tiloba_APRR1 QKNL--PDLHNHANSAMLPTYSHVPHCPPHMPGMGSFPYYPMNMCLQPGQMP--PQHPWP

I.batatas_APRR1 QKNL--PDLHNHANSAMLPTYSHVPHCPPHMPGMGSFPYYPMNMCLQPGQMP--PQHPWP

A.paniculata_APRR1 SSLGNSSNEGNCPSKVDRREAALIKFRQKRKERCFDKKIRYVNRKKLAERRPRLRGQFMR

O.europaea.europaea_APRR1 SQRNSSSNEGEL-SNVDRREAALMKFKQKRKERCFDKKIRYANRKQLAERRPRVKGQFVW

D.fullonum_APRR1 ------------------------------------------------------------

I.tiloba_APRR1 SYGSSSSADGKM-GKIDHREAALMKFRQKRKARCFDKKIRYVNRKRLADRRPRVRGQFVR

I.batatas_APRR1 SYGSSSSADGKM-GKIDHREAALMKFRQKRKARCFDKKIRYVNRKRLADRRPRVRGQFVR

A.paniculata_APRR1 KVNGVNVDLNGQPASAEEDE-EEEDYEEEDQSNI---SSMDDASTCL-

O.europaea.europaea_APRR1 KVNGVNVDLNGLPASA-----EDEVGEEEDQRANTDYLPEDNSSICH-

D.fullonum_APRR1 ------------------------------------------------

I.tiloba_APRR1 KPNGVLVDLNGHPASADDDEEDDEDEDDEDQTTTLDSSPEDDTSISLL

I.batatas_APRR1 KPNGVLVDLNGHPASADDDEEDDEDEDDEDQTTTLDSSPEDDTSISLL


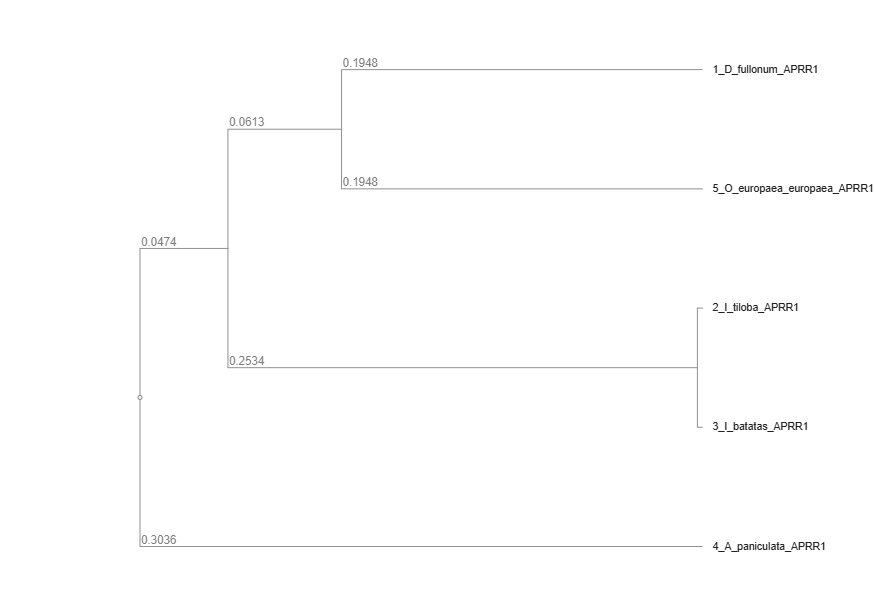


# DfAPX1

Domain: PLN02364

I.triloba_APX1 MGKCYPTVSEEYKKAIEKLRRKLRGFIADKNCAPLMLRLAWHSAGTYDVSSKTGGPFGTM

I.nil_APX1 MGKCYPTVSEEYQKAIEKLRRKLRGFIADKNCAPLMLRLAWHSAGTFDVSTKTGGPFGTM

D.fullonum_APX1 MVKCYPTVSEEYKKAIDKARRKLRGFIAEKNCAPLMLRLAWHSAGTYDVKTKTGGPFGTM

C.sinensis_APX1 MEKCYPTVSEEYKKAIDKAKRKLRGLIAEKNCAPIMLRLAWHSAGTYDVTTKTGGPFGTM

A.thaliana_APX1 MTKNYPTVSEDYKKAVEKCRRKLRGLIAEKNCAPIMVRLAWHSAGTFDCQSRTGGPFGTM

* * ******:* **::* :*****:**:*****:*:*********:* ::********

I.triloba_APX1 RLKAEQAHGANNGLDIAVRLLEPFKEQFPIVSYADFYQLAGVVAVEITGGPDVPFHPGRE

I.nil_APX1 RLKAEQAHGANNGLDIAVRLLEPFKEQFPIVSYADFYQLAGVVAVEITGGPDVPFHPGRE

D.fullonum_APX1 KLAAEQAHGANNGLDIAVRLLEPLKEQFPIISYADFYQLAGVVAVEVTGGPDVPFHPGRQ

R.vialii_APX1 RHKAEQGHGANNGLEIAVRLLEPIKEQFPILSYADFYQLAGVVAVEVTGGPDVPFHPGRP

C.sinensis_APX1 RHKLEQGHAANNGLEIAVRLLEPIKEQFPIISYADFYQLAGVVAVEITGGPDVPFHPGRE

A.thaliana_APX1 RFDAEQAHGANSGIHIALRLLDPIREQFPTISFADFHQLAGVVAVEVTGGPDIPFHPGRE

: * .*.**.*:.**:***:*::**** :*:***:*********:*****:******

I.triloba_APX1 DKTEPPVEGRLPDATQGNDHLRDVFVKQMGLSDKDIVALSGGHTLGRCHKERSGFEGPWT

I.nil_APX1 DKTEPPVEGRLPDATQGNDHLRDVFVKQMGLSDKDIVALSGGHTLGRCHKERSGFEGPWT

D.fullonum_APX1 DKAEPPVEGRLPDATKGNDHLRDVFVKNMGLTDKEIVALSWWTHPG--------------

R.vialii_APX1 DKTEPPVEGRLPDATKGTDHLRDVFVKQMGLSDQDIVALSGGHTLGRCHKERSGFEGPWT

C.sinensis_APX1 DKPEPPVEGRLPDATKGTDHLRDVFVKHMGLTDKDIVALSGGHTLGRCHKERSGFEGPWT

A.thaliana_APX1 DKPQPPPEGRLPDATKGCDHLRDVFAKQMGLSDKDIVALSGAHTLGRCHKDRSGFEGAWT

**.:** ********:* *******.*:***:*::***** *

I.triloba_APX1 TNPLIFDNSYFTELLSGEKEGLLQLPTDKALLNDPVFRPLVEKYAADEDAFFADYAEAHL

I.nil_APX1 TNPLIFDNSYFTELLSGEKEGLLQLPTDKALLNDPVFRPLVEKYAADEDAFFADYAEAHL

D.fullonum_APX1 ------------------------------------------------------------

R.vialii_APX1 ANPLIFDNSYFTELLTGEKEGLLQLPTDKALLSDPSFRPLVDKYAADEDAFFVDYAEAHM

C.sinensis_APX1 ANPLIFDNSYFTELLTGEKEGLLQLPSDKALLNDPVFRPLVEKYAADEDAFFADYAEAHM

A.thaliana_APX1 SNPLIFDNSYFKELLSGEKEGLLQLVSDKALLDDPVFRPLVEKYAADEDAFFADYAEAHM

I.triloba_APX1 KLSELGYAEA

I.nil_APX1 KLSELGYAEA

D.fullonum_APX1 ----------

R.vialii_APX1 KLSELGFAEA

C.sinensis_APX1 KLSELGFAEA

A.thaliana_APX1 KLSELGFADA


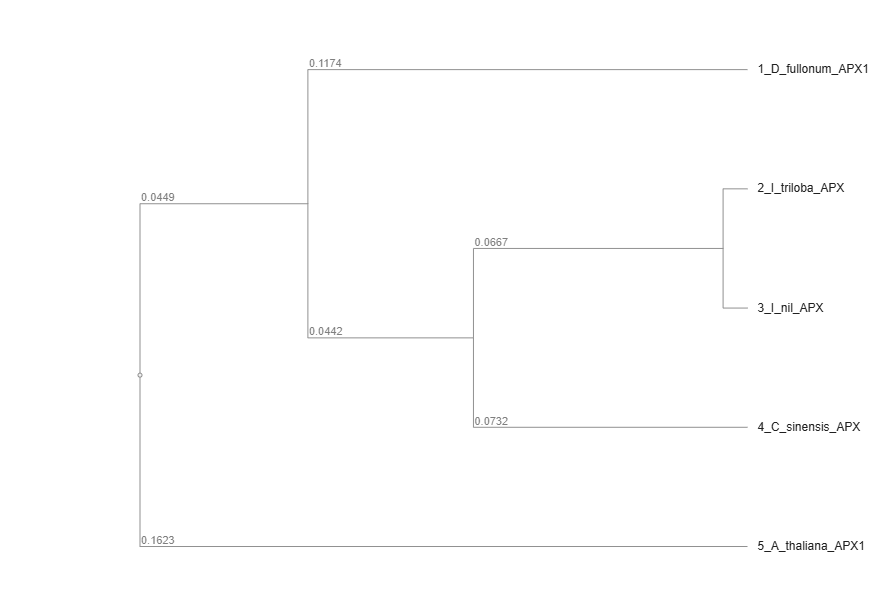


# DfCSD2

Domain: PLN02642

V.vinifera_CSD2 --------------------------------------------------

V.riparia_CSD2 --------------------------------------------------

D.fullonum_CSD2 --------------------------------------------------

P.trichocarpa_CSD2 --------------------------------------------------

P.granatum_CSD2 --------------------------------MEMRIPPENIIQNTAHET

E.grandis_CSD2 --------------------------------------------------

A.thaliana_CSD2 MAATNTILAFSSPSRLLIPPSSNPSTLRSSFRGVSLNNNNLHRLQSVSFA

V.vinifera_CSD2 -----------MGSVKAVAVIAGNANVRGSLHFIQDPAGSTHVKGRITGL

V.riparia_CSD2 -----------MGSVKAVAVIAGNANVRGSLHFIQDPAGSTHVKGRITGL

D.fullonum_CSD2 -----------MVKVKGVAIITGDAIVKGSLQFSQDEDGATHVKGRITGL

P.trichocarpa_CSD2 ---------MATGSVKAVALITGDSIVRGSLHFIQEPNGATHVTGRITGL

P.granatum_CSD2 EEDEEGTAAAAMGTIKAVALITGDVNVRGSLQFVQQPNGATQVKGKITGL

E.grandis_CSD2 -----------MSGVKAVALIIGDASVRGSLQFLQQPNGATHVKGRISGL

A.thaliana_CSD2 VKAPSKALTVVSAAKKAVAVLKGTSDVEGVVTLTQDDSGPTTVNVRITGL

*.**:: * *.* : : *: *.* *. :*:**

V.vinifera_CSD2 TPGLHGFHIHALGDTTNGCMSTGPHFNPLKKDHGAPTDKERHAGDLGNIV

V.riparia_CSD2 TPGLHGFHIHALGDTTNGCMSTGPHFNPLKKDHGSPTDKERHAGDLGNIV

D.fullonum_CSD2 NPGLHGFHIHALGDTTNGCNSTGPHYNPLKKNHGAPTDIERHAGDLGNIV

P.trichocarpa_CSD2 SPGLHGFHIHALGDTTNGCNSTGPHFNPLKKDHGAPSDKERHAGDLGNII

P.granatum_CSD2 SPGLHGFHIHALGDTTNGCNSTGPHFNPLKKDHGAPSDSERHAGDLGNIF

E.grandis_CSD2 SPGLHGFHIHALGDTTNGCNSTGPHFNPLKKNHGAPADSERHAGDLGNIV

A.thaliana_CSD2 TPGPHGFHLHEFGDTTNGCISTGPHFNPNNMTHGAPEDECRHAGDLGNIN

.** ****:* :******* *****:** : **:* * *********

V.vinifera_CSD2 AGPDGVAEVSIKDMQISLSGQHSILGRAVVVHADPDDLGRGGHELSKTTG

V.riparia_CSD2 AGPDGVAEVSIKDMQISLSGQHSILGRAVVVHADPDDLGRGGHELSKTTG

D.fullonum_CSD2 AGPDGVAEVSIRDMKIPLSGQHSILGRAVVVHADPDDLGRGGHELSKTTG

P.trichocarpa_CSD2 AGSDGVAEVSIKDLQIPLSGMHSILGRAVVVHADPDDLGKGGHELSKTTG

P.granatum_CSD2 AGHDGVAEVSITDLQIPLSGQHSILGRAVVVHADPDDLGRGGHELSKTTG

E.grandis_CSD2 AGPDGVAEVSIVDVQIPLTGQHSILGRAVVVHADPDDLGKGGHELSKTTG

A.thaliana_CSD2 ANADGVAETTIVDNQIPLTGPNSVVGRAFVVHELKDDLGKGGHELSLTTG

*. *****.:* * :*.*:* :*::***.*** ****:****** ***

V.vinifera_CSD2 NAGGRVGCGIIGLQSSA---------------------------------

V.riparia_CSD2 NAGGRVGCGIIGLQSSA---------------------------------

D.fullonum_CSD2 NAGARVGCGIILDFNQLFKNNCGNGPPPDLLFKFQLYPKLVMDALHKKSL

P.trichocarpa_CSD2 NAGARVGCGIVGLKSSV---------------------------------

P.granatum_CSD2 NAGARVGCGIIGLQSSV---------------------------------

E.grandis_CSD2 NAGARVGCGIIGLQSSV---------------------------------

A.thaliana_CSD2 NAGGRLACGVIGLTPL----------------------------------

***.*:.**::

V.vinifera_CSD2 -----

V.riparia_CSD2 -----

D.fullonum_CSD2 VQYKE

P.trichocarpa_CSD2 -----

P.granatum_CSD2 -----

E.grandis_CSD2 -----

A.thaliana_CSD2 -----


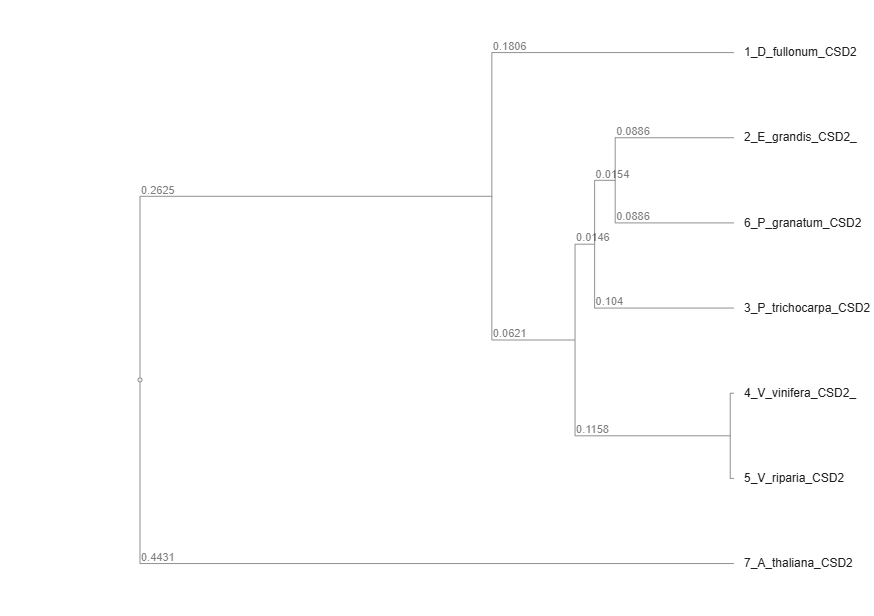


# DfCOP1

Domain: PLN00181

D.fullonum_COP1 ------------------------------------------------------------

S.tuberosum_COP1 MVESSIGGVVPAVKGEGMRRMGDKEEGGSV--RDEEVGTVTEWELDRELLCPICMQIIKD

S.pennellii_COP1 ------------------------------------------------------------

S.lycopersicum_COP1 MVESSVGGVVPAVKGEVMRRMGDKEEGGSVTLRDEEVGTVTEWELDRELLCPICMQIIKD

L.ferocissimum_COP1 MVESSIGGVVPLVKGEGM-------SSGVVRDEEEGVGTRIEWELDRELLCPICMQIIKD

D.fullonum_COP1 ------------------------------------------------------------

S.tuberosum_COP1 AFLTACGHSFCYMCIVTHLHNKSDCPCCSHYLTTSQLYPNFLLDKLMKKTSARQISKTAS

S.pennellii_COP1 ----------------------------------------------MKKTSARQISKTAS

S.lycopersicum_COP1 AFLTACGHSFCYMCIVTHLHNKSDCPCCSHYLTTSQLYPNFLLDKLLKKTSARQISKTAS

L.ferocissimum_COP1 AFLTACGHSFCYMCIVTHLHNKSDCPCCSHYLTTSQLYPNFLLDKLLKKTSARQISKTAS

D.fullonum_COP1 ------------------------------------------------------------

S.tuberosum_COP1 PVEQFRHSLEQGCEVSIKELDALLLMLSEKKRKLEQEEAERNMQILLDFLQMLRKQKVDE

S.pennellii_COP1 PVEQFRHSLEQGCEVSIKELDALLLMLSEKKRKLEQEEAERNMQILLDFLQMLRKQKVDE

S.lycopersicum_COP1 PVEQFRHSLEQGSEVSIKELDALLLMLSEKKRKLEQEEAERNMQILLDFLQMLRKQKVDE

L.ferocissimum_COP1 PVEQFRHSLEQGCEVSVKELDTLLSLLSEKKRKLEQEEAERNMQILLDFLQMLRKQKVDE

D.fullonum_COP1 -------------------------------------------MSTKAWSSSIDKNSSGL

S.tuberosum_COP1 LSEVQHDLQYIKEDLNSVERHRIDLYRARDRYSMKLRMLADDPIGKKPWSSSTDRNFGGL

S.pennellii_COP1 LSEVQHDLQYIKEDLNSVERHRIDLYRARDRYSMKLRMLADDPIGKKPWSSSTDRNFGGL

S.lycopersicum_COP1 LNEVQHDLQYIKEDLNSVERHRIDLYRARDRYSMKLRMLADDPIGKKPWSSSTDRNFGGL

L.ferocissimum_COP1 LNEVQNDLQYIKEDLNSVERHRIDLYRARDRYSMKLRMLADDPIGKKPWSSSTDRNFGGL

:..*.**** *:* .**

D.fullonum_COP1 ISTSQNTHGGMRTANFLYKKTEGKAQVSSLGGPQRKD-AFSGLSSQNMSQSGLAVARKKR

S.tuberosum_COP1 FSTSRNAPGGLPTGNLTYKKVDSKAQISS-PGPQRKDTSISELNSQHMSQSGLAVVRKKR

S.pennellii_COP1 FSTSRNAPGGLPTGNLTYKKVDSKAQISS-PGPQRKDTSISELNSQHMSQSGLAVVRKKR

S.lycopersicum_COP1 FSTSRNAPGGLPTGNLTFKKVDSKAQISS-PGPQRKDTSISELNSQHMSQSGLAVVRKKR

L.ferocissimum_COP1 FSASRNAPGGLPTGNLTYKKLDGRAQISS-PGPPRKDTSISEMNSQHMSQSGLAVVRKKR

:*:*:*: **: *.*: :** :.:**:** ** *** ::* :.**:********.****

D.fullonum_COP1 IMLQFNDLQECYLQKRRQLPNKSHNQEEGDTNVMQREGYTAGLSDFQSVLTTFTRYSRLR

S.tuberosum_COP1 VNAQFNDLQECYLQKRRQLANKSRVKEEKDADVVQREGYSEGLADFQSVLSTFTRYSRLR

S.pennellii_COP1 VNAQFNDLQECYLQKRRQLANKSRVKEEKDADVVQREGYSEGLADFQSVLSTFTRYSRLR

S.lycopersicum_COP1 VNAQFNDLQECYLQKRRQLANKSRVKEEKDADVVQREGYSEGLADFQSVLSTFTRYSRLR

L.ferocissimum_COP1 VNAQFNDLQECYLQKRRQLANKSRVKEEKDADVVQREGYSAGLADFQSVLSTFTRYSRLR

: ****************.***: :** *::*:*****: **:******:*********

D.fullonum_COP1 VIAELRHGDLFHSGNIVSSIEFDRDDGYFATAGVSRRIKVFDFSSVVNEPADVHCPVVEM

S.tuberosum_COP1 VIAELRHGDLFHSANIVSSIEFDRDDELFATAGVSRRIKVFDFSSVVNEPADAHCPVVEM

S.pennellii_COP1 VIAELRHGDLFHSANIVSSIEFDRDDELFATAGVSRRIKVFDFSSVVNEPADAHCPVVEM

S.lycopersicum_COP1 VIAELRHGDLFHSANIVSSIEFDRDDELFATAGVSRRIKVFDFSSVVNEPADAHCPVVEM

L.ferocissimum_COP1 VIAELRHGDLFHSANIVSSIEFDRDDELFATAGVSRRIKVFDFSSVVNEPADVHCPVVEM

*************.************ ************************.*******

D.fullonum_COP1 STRSKLSCLSWNKFTKNHIASSDYEGIVTVWDVNTRQSVMEYEEHEKRAWSVDFSRMEPS

S.tuberosum_COP1 STRSKLSCLSWNKYTKNHIASSDYDGIVTVWDVTTRQSVMEYEEHEKRAWSVDFSRTEPS

S.pennellii_COP1 STRSKLSCLSWNKYTKNHIASSDYDGIVTVWDVTTRQSVMEYEEHEKRAWSVDFSRTEPS

S.lycopersicum_COP1 STRSKLSCLSWNKYTKNHIASSDYDGIVTVWDVTTRQSVMEYEEHEKRAWSVDFSRTEPS

L.ferocissimum_COP1 STRSKLSCLSWNKYTKNHISSSDYDGIVTVWDVTTRQSVMEYEEHEKRAWSVDFSRTEPS

*************:*****:****:********.********************** ***

D.fullonum_COP1 MLVSGSDDCKVKIWCTNQESSVLNIDMKANICCVKYNPGSSFYVAAGSADHHIHYYDLRN

S.tuberosum_COP1 MLVSGSDDCKVKVWCTKQEASVLNIDMKANICCVKYNPGSSVHIAVGSADHHIHYYDLRN

S.pennellii_COP1 MLVSGSDDCKVKVWCTKQEASVLNIDMKANICCVKYNPGSSVHIAVGSADHHIHYYDLRN

S.lycopersicum_COP1 MLVSGSDDCKVKVWCTKQEASVLNIDMKANICCVKYNPGSSVHIAVGSADHHIHYYDLRN

L.ferocissimum_COP1 MLVSGSDDCKVKVWCTNQEASVLNIDMKANICCVKYNPGSSVHIAVGSADHHIHYYDLRN

************:***:**:*********************.::*.**************

D.fullonum_COP1 PSRPLHVFSGHKKTVSYVKFLSNNELASASTDSTLRLWDVKDNLPVRTFRGHTNEKNFVG

S.tuberosum_COP1 TSQPVHIFSGHRKAVSYVKFLSNNELASASTDSTLRLWDVKDNLPVRTLRGHTNEKNFVG

S.pennellii_COP1 TSQPVHIFSGHRKAVSYVKFLSNNELASASTDSTLRLWDVKDNLPVRTLRGHTNEKNFVG

S.lycopersicum_COP1 TSQPVHIFSGHRKAVSYVKFLSNNELASASTDSTLRLWDVKDNLPVRTLRGHTNEKNFVG

L.ferocissimum_COP1 TSQPVHIFSGHRKTVSYVKFLSNNELASASTDSTLRLWDVNENLPVRTLRGHMNEKNFVG

.*:*:*:****:*:**************************::******:*** *******

D.fullonum_COP1 LSVSSEFIACGSETNEVFVYHKAISKPVNWHRFGSPDVDESDEDAGSYFISAVCWKSDSP

S.tuberosum_COP1 LSVNNEFLSCGSETNEVFVYHKAISKPVTWHRFGSPDIDEADEDAGSYFISAVCWKSDSP

S.pennellii_COP1 LSVNNEFLSCGSETNEVFVYHKAISKPVTWHRFGSPDIDEADEDAGSYFISAVCWKSDSP

S.lycopersicum_COP1 LSVNNEFLSCGSETNEVFVYHKAISKPVTWHRFGSPDIDEADEDAGSYFISAVCWKSDSP

L.ferocissimum_COP1 LSVNNEFLSCGSETNEVFVYHKAISKPVTWHRFGSPDVDEADEDAGSYFISAACWKSDSP

***..**::*******************.********:**:***********.*******

D.fullonum_COP1 TMLAANSQGTIKVLVLAA

S.tuberosum_COP1 TMLAANSQGTIKVLVLAA

S.pennellii_COP1 TMLAANSQGTIKVLVLAA

S.lycopersicum_COP1 TMLAANSQGTIKVLVLAA

L.ferocissimum_COP1 TMLAANSQGTIKVLVLAA

******************


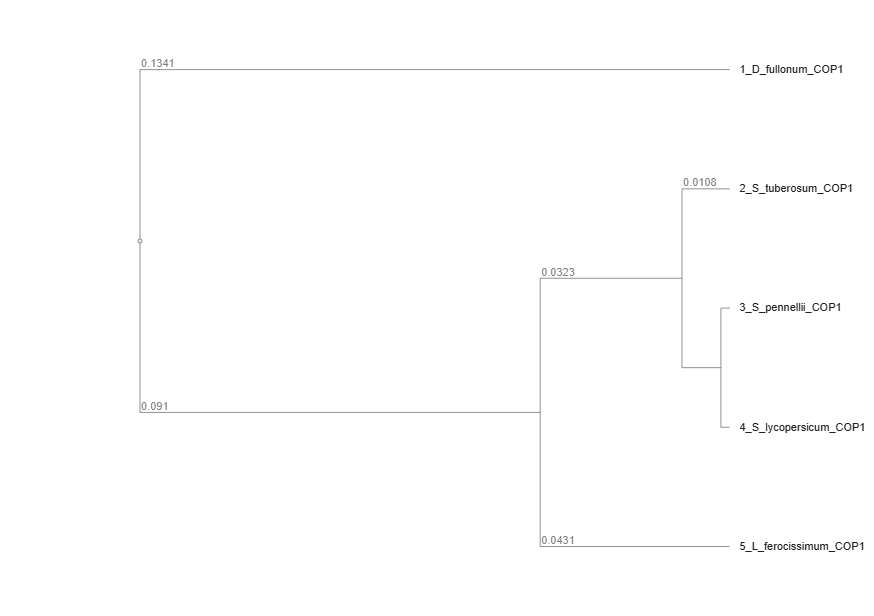


# DfCOP9

Domain: cd08069

D.fullonum_COP9 MGTALKTWELENSVKLVDPQKDALYNYSSSEQKAINNAHPWRTDPNYFTSVRISAIALLK

A.arborescens_COP9 MGTALKTWELENSVKLVDPQKDALYNYSSSEQKAINNAHPWRTDPNYFTSVRISAIALLK

A.gaisen_COP9 MGTALKTWELENSVRLVDPQKDALYNYSSSEQKAINNAHPWRTDPNYFTSVRISAIALLK

A.panax_COP9 MGTALKTWELENSVKLVDAQKDALYNYSSDEQKAINNAHPWRSDPNYFTSVRISAIALLK

**************:***.**********.************:*****************

D.fullonum_COP9 MVMHARSGGSIEVMGLMLGKIEAHTFVVTDAFRLPVEGTETRVNAQDEANEYMVEFLQRA

A.arborescens_COP9 MVMHARSGGSIEVMGLMLGKIEAHTFVVTDAFRLPVEGTETRVNAQDEANEYMVEFLQRA

A.gaisen_COP9 MVMHARSGGSIEVMGLMLGKIEAHTFVVTDAFRLPVEGTETRVNAQDEANEYMVEFLQRA

A.panax_COP9 MVMHARSGGSIEVMGLMLGKIEAHTFVVTDAFRLPVEGTETRVNAQDEANEYMVEFLQRA

************************************************************

D.fullonum_COP9 REQGQMENAVGWYHSHPGYGCWLSGIDVNTHRRLNNSSKTPSAPSSSIPTELSLRAKSRS

A.arborescens_COP9 REQGQMENAVGWYHSHPGYGCWLSGIDVNTQK----TQQQFQDPFCAIVIDPDRTVSAGK

A.gaisen_COP9 REQGQMENAVGWYHSHPGYGCWLSGIDVNTQK----TQQQFQDPFCAIVIDPDRTVSAGK

A.panax_COP9 REQGQMENAVGWYHSHPGYGCWLSGIDVNTQK----TQQQFQDPFCAIVIDPDRTVSAGK

******************************:: :.: . * .:* : . ..: .

D.fullonum_COP9 VPSVPIQQSTSRIRPRQVAAPKTQVALRATASKPFRSARLRTLA--------HTPTTTTP

A.arborescens_COP9 VEIGAFRTYSTEYVENQAKAGGGSKNTSGAESDGFETIPLGKIEDFGAHANHYYPLEVSH

A.gaisen_COP9 VEIGAFRTYSTEYVENQANAGGGSKNTSGAESDGFETIPLGKIEDFGAHANHYYPLEVSH

A.panax_COP9 VEIGAFRTYSTEYVENQANSGGSSKNTGGADSDGFETIPLGKIEDFGAHANHYYPLEVSH

* .:: ::. .*. : . .: *. *.: * .: : * .:

D.fullonum_COP9 SKSRT-----------------------------TSRHSTLSYK----------------

A.arborescens_COP9 YKSSLDAKLLEALWNKYWVQTLSSSPLISNRDYGTKQISDLARKMQQENNSSKRFKGGAG

A.gaisen_COP9 YKSSLDAKLLEALWNKYWVQTLSSSPLISNRDYGTKQISDLARKMQQENNSSKRFKGGAG

A.panax_COP9 YKSSLDAKLLEALWNKYWVQTLSSSPLISNRDYGTKQISDLARKMQQENASGKRFKGGAG

** *.: * *: *

D.fullonum_COP9 ---------------------------------------------------------

A.arborescens_COP9 YATNSESKNQLTKLGAAGSKIAREEDMGLLAANVKDKVFNLTNGQEVKSQEVEMETS

A.gaisen_COP9 YATNSESKNQLTKLGAAGSKIAREEDMGLLAANVKDKVFNLANGQEVKSQEVEMETS

A.panax_COP9 YATNNESKNQLTKLGAAGSKIAREEDMGLLAAKVKDKVFNLANGEEIKSQEVEMETS


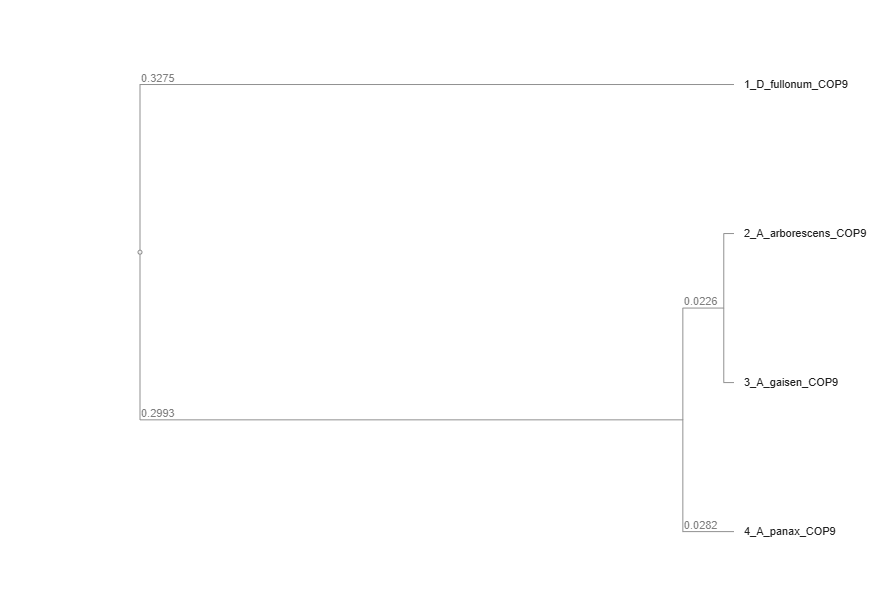


# DfDAG1

D.fullonum_DAG1 MATINLSLFSKTLAL-ISPQKPPTPLLTVPSRLNLSSFNTFHSLFFPRRSLNPT--PCRT

D.carotasativus_DAG1 MATLNLSPLSKTLIN-PTPLKPSTPLSCLSLSYPLQSLS-----FSLTQNLKPTRNSSRT

S.hispanica_DAG1 MATLNLSSFSKTLNP-IS--KTLTPKLSHPSTLSVS----FLPCARPYLTKSRS--AALP

C.sinensis_DAG1 MATLNLSLFSKTLKTQYSTYLNPTTTLLLTHSGPFRSWA--------GPHSNPT--LSRT

S.splendens_DAG1 MAALNLTSFSKTLNP-IS--KTLTPKISLPSTLSVS----FLPCARPYLTKSRN--AALP

**::**: :**** : *. . . . . . .

D.fullonum_DAG1 PVRAAVDGDYSAKRSSSNEQRETIMLPGCDYNHWLIVMEFPKDPAPTREQMIDTYLDTLA

D.carotasativus_DAG1 LIRAAVDGDYSAKRSNSSEPRETIMLPGCDYNHWLIVMEFPKDPAPTREQMIDTYLETLA

S.hispanica_DAG1 AVRALTDGEYSAKRNSGGEERETILLPGCDYNHWLIVMEFPKDPAPTRDQMIDTYLNTLA

C.sinensis_DAG1 RTRAALDGDYSSKRSSSNDPRETLMLPGCDYNHWLIVMEFPKDPAPTREQMIETYLTTLA

S.splendens_DAG1 AVRALTDGEYSAKRNSGGEERETILLPGCDYNHWLIVMEFPKDPAPTRDQMIDTYLNTLA

** **:**:**....: ***::***********************:***:*** ***

D.fullonum_DAG1 TVLGSMEEAKKNMYAFSTTTYTGFQCTVSEETSEKFKGLPGVLWVLPDSYIDVKNKDYGG

D.carotasativus_DAG1 TVLGSMEEAKKNMYAFSTTTYTGFQCTVSEEVSEKFKGLPGVLWVLPDSYIDVKNKDYGG

S.hispanica_DAG1 TVLGSMEEAKKNMYAFSTTTYTGFQCTVSEETSEKFKGLPGVLWVLPDSYIDVKNKDYGG

C.sinensis_DAG1 TVLGSMEEAKKSMYAFSTTTYTGFQCTVSEETSEKFKGLPGVLWVLPDSYIDVKNKDYGG

S.splendens_DAG1 TVLGSMEEAKKNMYAFSTTTYTGFQCTVSEETSEKFKGLPGVLWVLPDSYIDVKNKDYGG

***********.*******************.****************************

D.fullonum_DAG1 DKYVNGEIIPCTYPTYQPKQNKRTSKYESKRYVRQRDGPPAVKRTPKQEATPSESSSSVT

D.carotasativus_DAG1 DKYVNGEIIPCTYPTYQPKQQRR-SKYESKRYVRQRDGPPPERRKPKQ-ATESPSG----

S.hispanica_DAG1 DKYVNGEIIPCQYPTYQPKQARS-SKYKSKAYVRKRDGPPSEQRKPRQEATPESAS----

C.sinensis_DAG1 DKYINGEIIPCKYPTYQPKQSNR-SKYESKRYVRQRDGRPAERRRPTQEATSSESTS---

S.splendens_DAG1 DKYVNGEIIPCQYPTYQPKQARS-SKYKSKAYVRKRDGPPSEQRKPRQEATPESAS----

***:******* ******** . **::** ***:*** *. :* * * *: . :

D.fullonum_DAG1 IPKNIFSCFGPNPFMQILQMNYLMEDARIFLIGHVLKEELYKIHIKVVYEK

D.carotasativus_DAG1 ---------------------------------------------------

S.hispanica_DAG1 ---------------------------------------------------

C.sinensis_DAG1 ---------------------------------------------------

S.splendens_DAG1 ---------------------------------------------------


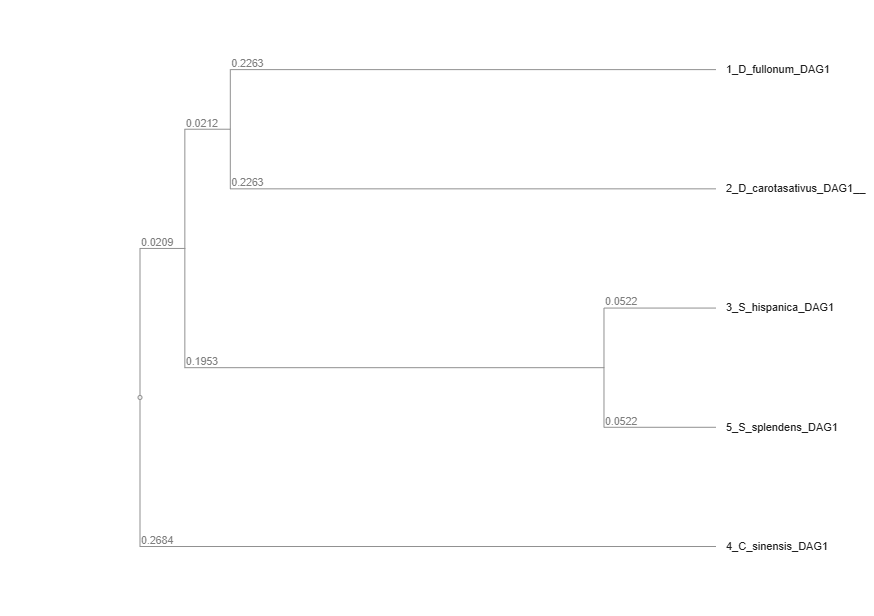


# DfDOG1

Domain: pfam14144

D.fullonum_DOG1 MGFHRFYESWFDELHVLVHQLNKAPKPAITREHHTFHLQLLNTVISHFVDYYQVKNAAVK

L.ferocissimum_DOG1 MSFQRFYQTWFDQLKEIVHQLNQAPKPATNDQHRELHQQLVQKVMSHYYEYYRVKSLAAK

D.lotus_DOG1 MSFHGFYQTWFDHLRRMVDQLAEARRPPATQEDHHALRQLVHKVMTHYAEYYRVKSLAAK

I.triloba_DOG1 MSFQSFHETWFEQLRQVVHELGQAPRPATSAEHHHLQQQLVQKVMSHCHDYYRLKSIAAK

C.sinensis_DOG1 MSFHRFYQTWFDHLRRLVDQLTQAPRPPTTEEDHHILHQLVHMVTSHYAEYYRVKSLSSK

N.attenuata_DOG1 MSFQSFYETWFEQLKGMVHQLNQAPKPATNDQNHELHQQLVQKVMSHYSEYYRVKSLAAK

*.*: *:::**:.*: :*.:* :* :*. . :.: **:: * :* :**::*. : *

D.fullonum_DOG1 HDVLSVYAAPWCTSLERSLQWIGGWRPTTAFHLLYTQSSILFESNILDILNGVRTGGLGD

L.ferocissimum_DOG1 NDILSVFAAPWSTSLERSLHWIAGWRPTTAFHLIYTESSILFESHIIDILRGLRYGDLGD

D.lotus_DOG1 HDVVSVFAEPWCTALERSLHWIGGWRPTTAFHLVYTESSILFESHVIDILRGLRTGDLGD

I.triloba_DOG1 RDILGVFTAPWATSLERSLHWIGGWRPTTVFHLIYTESSILFESHIMDILRGLRNGDLGD

C.sinensis_DOG1 HDVFSLFAAPWSTSLERSLHWIAGWRPTTAFHLIYTESSIRFESHVVDILRGLRTGDLGD

N.attenuata_DOG1 NDILSVFYAPWCTSLERSLHWIAGWRPTTAFHLIYTESSVLFESHIIDILRGLRYGDLGD

.*:..:: **.*:*****:**.******.***:**:**: ***:::***.*:* *.***

D.fullonum_DOG1 LTPTQFNRVSELQCQTVDEENFISTQLSNWQDGSSDGKDEY-FELSEKLQRLVSIVEKAD

L.ferocissimum_DOG1 LSPDQLSRVSELQCEAVHEENAISDELSDWQDGASEIIGLM-GDIDTKMEMLVEILEKAD

D.lotus_DOG1 LSPAQFRRVSELQCDTVREENSITDELSDWQDCSSDLLGVC-TNVDGKIERLVRILEKAD

I.triloba_DOG1 LTPSQLRRVSELQCQTVQEENYIADQLSDWQDGGSDIIGMMSGNLEAKMEKLAEILEKAD

C.sinensis_DOG1 LSPGQFRRVSELQCDTVREENAITDELSEWQV----------------------------

N.attenuata_DOG1 LSPDQLRRVSELQCQAVLEENAIAHELSEWQDGASEVIGLM-GDIDAKMEGLVSVLERAD

*:* *: *******::* *** *: :**:**

D.fullonum_DOG1 ELRLRTITRVVELLTPQQSIEFLIVASELHSGIHGWESDNEHRKK-----

L.ferocissimum_DOG1 KLRMKTIENLVELLTPQQAVEFLIASAYLQFGIRRWGINHDRQRGNP---

D.lotus_DOG1 DLRLRTVRKVVDLLTPQQGVEFLVAAAEFHFGVRSWGLNHDRLRRK----

I.triloba_DOG1 ELRMKTITNLVELLTAQQAVEFLIAAAQLMFGIRRWGINHDRRRENNVSQ

C.sinensis_DOG1 --------------------------------------------------

N.attenuata_DOG1 KLRMKTIENLVQLLSPQQAVEFLIAAAHLQFGIRRWGINHDRQRGNL---


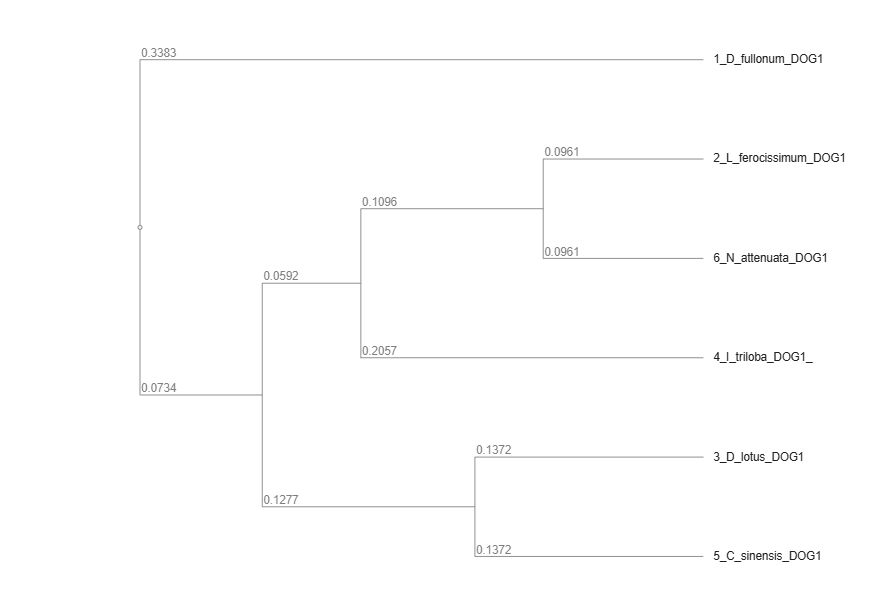


# DfEM6

Domain: pfam00477

D.fullonum_EM6 MASDQDRKGLDERAKHGETVVPGGTGGKSLEAQEHLAEGRSKGGQTRKGQIGTEGYQEMG

T.pratense_EM6 MASQQNRKELDEKAKQGETVVPGGTGGKSLEAQEHLAEGRSKGGQTRKEQLGTEGYQQMG

Q.robur_EM6 MSSQQKRQELDARARQGETVVPGGTGGKSLEAQEHLAEGRSRGGQTRRKQLGTEGYQEMG

E.grandis_EM6 MASRQEREELDRRAKEGETVVPGGTGGKSLEAQEHLAEGRSKGGQTRKEQLGTEGYQQMG

M.rubra_EM6 MSSKQERAELDARARQGETVIPGGTGGKSLEAQEHLAEGRSRGGQTRKEQLGTEGFQEMG

*:* *.* ** :*:.****:********************:*****: *:****:*:**

D.fullonum_EM6 RKGGLSTNEESGRERAEREGIPIDESKFRNKS-

T.pratense_EM6 RKGGLSTMEKSGGERAEEEGIDIDESKFKNQNK

Q.robur_EM6 RKGGLSTGDMSGEERAKEEGIPIDESKFRTKS-

E.grandis_EM6 RKGGLSTGDESGGERTAREGIEIDESKFRTKS-

M.rubra_EM6 RKGGLSTTDKSGEERAREERIPIDESKFRTNS-

******* : ** **: .* * ******:.:.


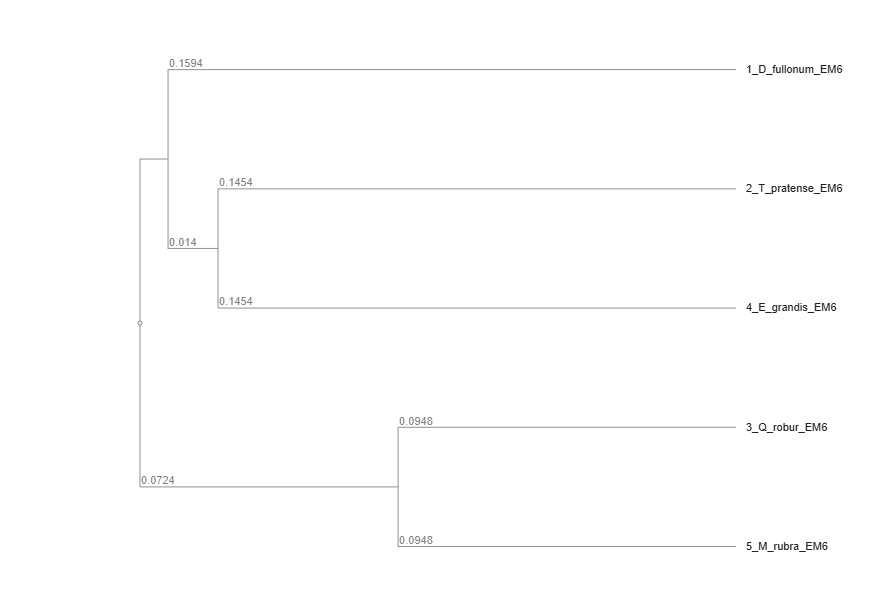


# DfGAI

Domain: pfam12041 (yellow); pfam03514 (blue)

A.thaliana_GAI --MKRDHHHHHHQDKKTMMMN-----------EEDDGNGMDELLAVLGYK

C.florida_GAI --MKREREGHHHDKGQTYPSSSMAAAGKQKLWEEDSAGGMDELLAVLGYK

A.eriantha_GAI ----MKRDRDRDKAESSSMAATAAEKGKMWTEEQADVGGMDELLAVLGYK

S.miltiorrhiza_GAI --------MKRDRDRGKAAAAGKPK----MWAEPQADAGMDDLFAVLGYK

M.indica_GAI -MKKDHQESCGGGGVGGGNIKGESSSKAKMWEDKQDTGGMDELLEALGYK

D.fullonum_GAI MVNKRDRERDKGGGSSSMAAPAAVKPKIWEEVPLDAAAGMDELLAILGYK

***:*: ****

A.thaliana_GAI VRSSEMADVAQKLEQLEVMMSN--VQEDDLSQLATETVHYNPAELYTWLD

C.florida_GAI VRSSDMADVAQKLEQLEMVMS----QEDGISHLSCDTVHYNPSDLSGWVQ

A.eriantha_GAI VKASDMLEVAHKLEQLEMVME-----DDGISHLSSDTVHYNPSDLSGWVQ

S.miltiorrhiza_GAI VKSSDMADVAEKLEQLEMAMGT--TADDGVSVLANDSVHYNPSDLSGWVE

M.indica_GAI VRASDMADVALKLEQLEMVMGT--AQEDGISHLATDTVHYNPSDLSGWVE

D.fullonum_GAI VRSSEMSDVADKLEQLEMAMGT--TMKDGVSHLATDTVHYNPSDVSGFVD

*::*:* :** ******: * .*.:* *: :::****::: :::

A.thaliana_GAI SMLTDLNPP-----------------------------------------

C.florida_GAI SMLSEFNTSPLCNFDAIQSQNSTVDDAMLIPGESSTNINGFDYYSNSGQL

A.eriantha_GAI SMLSELNGTG----FDIGPAESTIIDDSLLVPSDNNSSIVSSSIDFAPSQ

S.miltiorrhiza_GAI SMLSELSSSCG-------------FDNDMG----------------ISGE

M.indica_GAI SMLSELNNTTFDTQ-----------------PRPIPDPAESSTFNPIQQA

D.fullonum_GAI NMLSELSTGNFES-------------------------STMIDFSNSNQV

.:*:::

A.thaliana_GAI -------SSNAEYDLKAIPGDAILN-----QFAIDSASSSNQGGGGDTYT

C.florida_GAI ENQSRIFEDNSEYDLRAIPGGAILKN----REAPGLERESGIKRLKSSIG

A.eriantha_GAI SQQSQIYEDNSEYDLSAIPGGAICK------------QNESSAENGNKRR

S.miltiorrhiza_GAI SSSSKFINFSGDDDLRAIPGGAVFG---------NKQKEESE--------

M.indica_GAI NNQSRIFNDDSEYDLRAIPGVAAYP----------PQPDSEAENNRNKRV

D.fullonum_GAI DQRGSIFNDDSEYDLRAIPGGAIYGNNSDSKSDGSKRMKSVGSEFGTNTV

: ** **** * ..

A.thaliana_GAI TNKRLKCSNGVVETTTATAESTRHVVLVDSQENGVRLVHALLACAEAVQK

C.florida_GAI SECALAPVQPVPEIGAVAAEPPRPVVLVDSQETGIRLVHSLMACAEAVQE

A.eriantha_GAI KTVGNRSGEAVLAEVCGGPTAARPVVLVDSQETGVRLVHTLMACAEAVQQ

S.miltiorrhiza_GAI NGSKRMKGSEFAENCSPASATR--SVVVDSQETGVRLVHTLMACAEAVQQ

M.indica_GAI KTSVNPSGASGSGVAGSNPESTRPVMVIDSQEAGVQLVHTLLACAEAVQQ

D.fullonum_GAI STPVDVPVVPPVVQEQPQPQQQQRSLVVDSQETGIRLVHTLMACGEAVQQ

. :::**** *::***:*:**.****.

A.thaliana_GAI ENLTVAEALVKQIGFLAVSQIGAMRKVATYFAEALARRIYRLSPSQSPID

C.floridaGAIL DNLKLADALVKHIGLLAVSQAGAMRKVANYFAQALARRIYKIYP-QTSVD

A.eriantha_GAI GNLKLADALVKHIGVLAVSQAGAMRKVATYFAEALARRIYKIYP-QESND

S.miltiorrhiza_GAI ENMKLADALVKHVGLLAVSQVGAMRKVATYFAEALARRIYKIYP-HETLD

M.indica_GAI DNLKLADALVKHIGLLAASQAGAMRKVATYFAEALARRIYRIYP-QDALE

D.fullonum_GAI ENLKLADALLKHIVYLAVSQTGAMRKVATYFAEALARRIYKIYP-QDSLE

*:.:*:**:*:: **.** *******.***:*******:: * : . :

A.thaliana_GAI HSLSDTLQMHFYETCPYLKFAHFTANQAILEAFQGKKRVHVIDFSMSQGL

C.florida_GAI NSYSDMLQMHFYETCPYLKFAHFTANQAILEAFAGANRVHVIDFSLKQGM

A.eriantha_GAI TSYSDLLEMHFYETCPYLKFAHFTANQAILEAFAGATRVHVIDFSLKQGM

S.miltiorrhiza_GAI SSLSDILQMHFYETGPYLKFAHFTANQAILEAFAGANRVHVIDFSLRQGM

M.indica_GAI SSYNDILQMHFYETCPYLKFAHFTANQAILEAFATANRVHVIDFSLKQGM

D.fullonum_GAI TSYSDILEMHFYETCPYLKFAHFTANQAILEAFAGAI-------------

* .* *:*****: ******************

A.thaliana_GAI QWPALMQALALRPGGPPVFRLTGIGPPAPDNFDYLHEVGCKLAHLAEAIH

C.florida_GAI QWPALMQALALRPGGPPAFRLTGIGPPQPDNTDALRQVGWKLAQLAETIG

A.eriantha_GAI QWPALMQALALRPGGPPALRLTGIGPPQPDNTDALQQVGWKLAQLAERIG

S.miltiorrhiza_GAI QWPALMQALALRPGGPPAFRLTGIGPPQPDNSDALQQVGWKLAQLAETIG

M.indica_GAI QWPALMQALALRPGGPPAFRLTGIGPPQPDNTDALQQVGWKLAQFADNIG

D.fullonum_GAI --------------------------------------------------

A.thaliana_GAI VEFEYRGFVANTLADLDASMLELRPSEIESVAVNSVFELHKLLGRPGAID

C.florida_GAI VEFEFRGFVTNSLADLDAAMLDIRPSEVEAVAVNSVFELHRLLARPGAID

A.eriantha_GAI VQFEFRGFVANSLADLDAAILDIRPSAAEAVAVNSMFELHRLLARAGAID

S.miltiorrhiza_GAI VEFEFRGFVTNSLADLDADMLEIRGGDEETVAVNSVFELHQLLARPGAIE

M.indica_GAI VEFEFRGFVANSLADLEPEMLDIRP-EVETVAVNSVFELHRLLARPGGIE

D.fullonum_GAI --------------------------------------------------

A.thaliana_GAI KVLGVVNQIKPEIFTVVEQESNHNSPIFLDRFTESLHYYSTLFDSLEG--

C.florida_GAI KVLSSIKAVKPKIVTIVEQEANHNEPVFVNRFNEALHYYSTMFDSLEGSG

A.eriantha_GAI KVLASIKAMRPKIVTVVEQEANHNGSGLVERFNEALHYYSTMFDSLESSG

S.miltiorrhiza_GAI KVLDSVKAMRPKIVTVVEQEANHNGSAFLDRFNEALHYYSTMFDSLECSE

M.indica_GAI KVVASIKAMKPKIITIVEQEANHNSPVFLDRFTEALHYYSSLFDSLEGSS

D.fullonum_GAI --------------------------------------------------

A.thaliana_GAI ---VPSGQDKVMSEVYLGKQICNVVACDGPDRVERHETLSQWRNRFGSAG

C.florida_GAI LTQPNTHDDLAMSELYLGRQICNVVACEGTDRVERHETLTQWRARMSSSG

A.eriantha_GAI -LTQVSNQDLVMSEAYLGRQICNVVACEGTDRVERHETLSQWRTRMGSAG

S.miltiorrhiza_GAI STQPDGTQDLMMTEVYLGRQICNVVACDGAERVERHETLAQWRGRMNSAG

M.indica_GAI --VTPGSQDLAMSEVYLGMQICNVVAYEGSDRTERHETLTQWRARLTSAG

D.fullonum_GAI --------------------------------------------------

A.thaliana_GAI FAAAHIGSNAFKQASMLLALFNGGEGYRVEESDGCLMLGWHTRPLIATSA

C.florida_GAI FDPAHLGSNAFKQASMLLALFAGGDGYRVEENNGCLMLGWHTRPLIATSA

A.eriantha_GAI FDPAHLGSNAFKQASMLLALFAGGDGYRVEENDGCLMLGWHTRPLIATSA

S.miltiorrhiza_GAI FDPVHLGSNAFKQASMLLALFSSGDGYRVEESDGSLMLGWHTRPLIATSA

M.indica_GAI FDPVHLGSNAFKQASMLLALFAGGDGYRVEENNGCLMLGWHTRSLIATTA

D.fullonum_GAI --------------------------------------------------

A.thaliana_GAI WKLSTN----

C.florida_GAI WKLGTGE---

A.eriantha_GAI WQLAATAS--

S.miltiorrhiza_GAI WRITAADQ--

M.indica_GAI WQLAGSES--

D.fullonum_GAI ----------


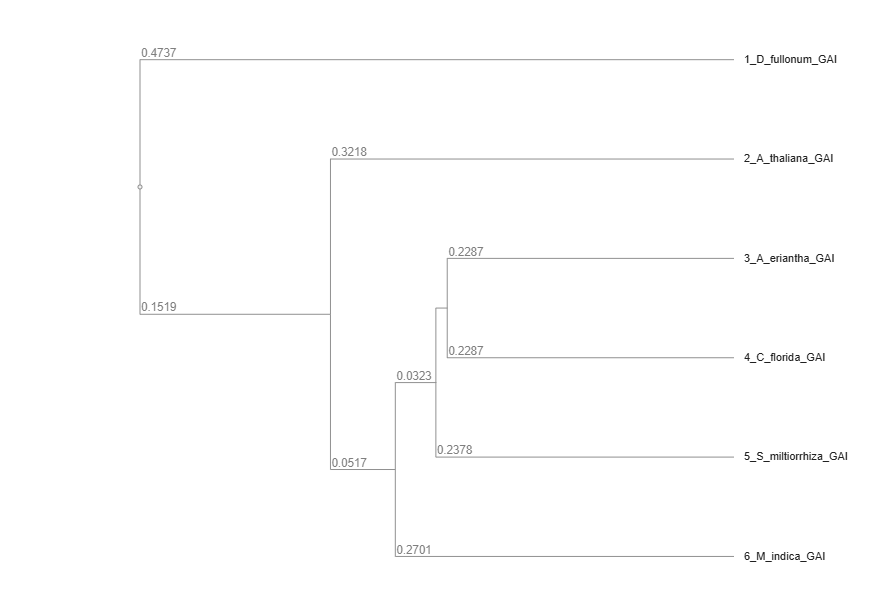


# DfGA3ox1

Domain: PLN02254

D.fullonum_GA3ox1 ------------------------------------------------------------

C.lanceoleosa_GA3ox1 MPSRVPEA-------SHHKHLDFNSVKELPESHAWTSLLGDYPSGPDSCGVGSVPIIDLD

C.sinensis_GA3ox1 MPSRVPDA-------SHHKHLDFNSVKELPESHAWTSLLGDYPSGPDSCGVGSVPIIDLD

Q.lobata_GA3ox1 MPSRLSDAFKAHPVQLHPKHQDFNSLQELPDSYKWTQ-LDEYPS-CESYSSESVPLIDVT

D.fullonum_GA3ox1 --------------------------------MEASGRKLFSLPIQQKLKAARAPDGVSG

C.lanceoleosa_GA3ox1 DPNAQKLVGHACSTWGVFQVTNHGIKNSLLNGIEAAGKSLFSLPIQQKLKAARSPDGVSG

C.sinensis_GA3ox1 DPNAQKLVGHACSTWGVFQVTNHGVKNSLLNSIEAAGKSLFSLPIQQKLKAARSPDGVSG

Q.lobata_GA3ox1 DPNAHTLIGHACKTWGVFQVTNHGIPTKLLDDIESVSRCLFSLPQQQKLKAARSPDGVSG

:*: .: ***** ********:******

D.fullonum_GA3ox1 YGVARISSFFPKLMWSEGFTILGSPLEHARQLWPQDYSEFCDVIQEYEKEMKRLAGRLMW

C.lanceoleosa_GA3ox1 YGVARISSFFPKLMWSEGFTIFGSPLEHARQLWPQDHNQFCDVIEEYEKVMQQLAGRLMW

C.sinensis_GA3ox1 YGVARISSFFPKLMWSEGFTIFGSPLEHARQLWPQDHNQFCHVIEEYEKVMQQLAGRLMW

Q.lobata_GA3ox1 YGLARISSFFPKLMWSEGFTIVGSPLEHFRQLWPQEYSKFCDIIEEYEKEMKKLAGRLMW

**:******************.****** ******::.:**.:*:**** *::*******

D.fullonum_GA3ox1 LMLGSLGIATEDVKWAGPKEEFNASSAALQLNSYPACPDPERAMGLAAHTDSTLLTLLHQ

C.lanceoleosa_GA3ox1 LMLGSLGITKEDIKWAGPKANFKGASAAIQLNSYPACPDPDRAMGLAAHTDSTLLTILHQ

C.sinensis_GA3ox1 LMLGSLGITKEDIKWAGPKADFKGASAAIQLNSYPACPDPDRAMGLAAHTDSTLLTILHQ

Q.lobata_GA3ox1 LILGSLGICNEDIKWAGPKGEFNEASAALQLNSYPACPDPDQAMGLAAHTDSTLLTILHQ

*:****** .**:****** :*: :***:***********::**************:***

D.fullonum_GA3ox1 NNTSGLQVHREGSGWVTVDPLPGALVVNIGDLFHILSNGLYPSVLHRAVVNRTHHRLSIA

C.lanceoleosa_GA3ox1 NNTSGLQVLREGAGWVTVPPRPGTLVVNIGDLLHILSNGLYPSVLHRAMVNRTQHRLSIA

C.sinensis_GA3ox1 NNTSGLQVLREGAGWVTVPPQPGTLVVNIGDLLHILSNGLYPSVLHRAMVNRTQHRLSIA

Q.lobata_GA3ox1 NNTSGLQVLREGTGWVTVEPLQGALVVNIGDLLHILSNGLYPSVLHRAMVNRTRYRLSVA

******** ***:***** * *:********:***************:****::***:*

D.fullonum_GA3ox1 YLYGPPSSVQISPLSKLVDNGHPPLYRPVTWSEYLGTKAKHFNKALSSVRLCVPLNG---

C.lanceoleosa_GA3ox1 YLYGPPTNVQISPLSKLVDPSHPPLYRPVTWTEYLGTKAKHFNKALSSVRLCIPLNGLVD

C.sinensis_GA3ox1 YLYGPPTNVQISPLSKLVDPSHPPLYRPVTWTEYLGTKAKHFNKALSSVRLCIPLNGLVD

Q.lobata_GA3ox1 YLYGPPASVQISPLSKLLGPSHPPLYRPVTWNEYLGTKAKHFNQALSSVRLCVPLNGLVD

******:.*********:. .**********.***********:********:****

D.fullonum_GA3ox1 --------------------

C.lanceoleosa_GA3ox1 VNDHNRVEVSWRQLPYIKMM

C.sinensis_GA3ox1 VNDHNRVEVSWRQLPYIKMM

Q.lobata_GA3ox1 VNDHNRVQVG----------


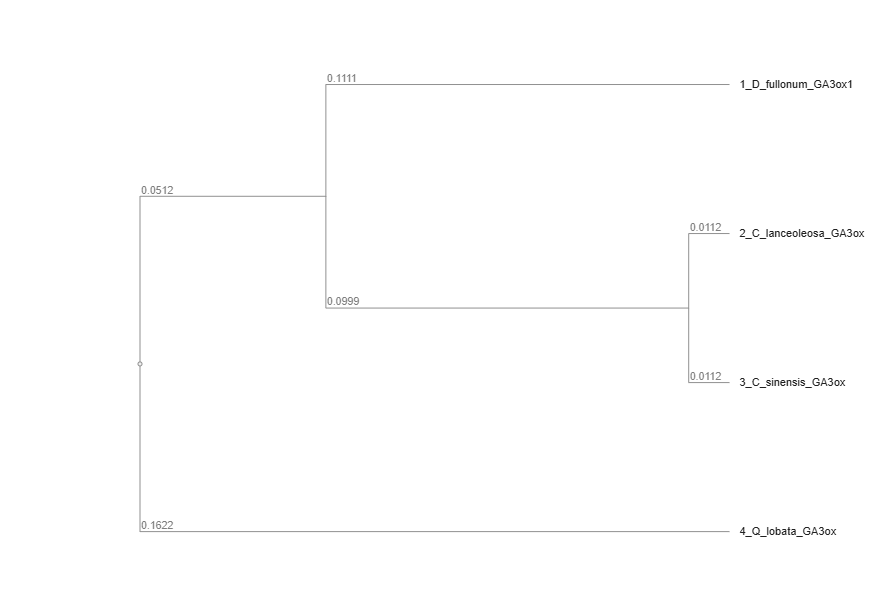


# DfGSTU

Domain: cd03185

D.fullonum_GSTU ---------------MFGMRVRIALAEKGIEYEYKEEDLSNKSPLLLKMNPVHKKIPVLI

C.florida_GSTU --MADEVILLDSLPSMFGMRVRIALAEKGIEYERKEEDLSNKSPLLLKMNPVHKKIPVLI

I.trifida_GSTU MAGGEEVVLLDSYASMFGMRVKVALAEKGIQYVHKEEDLRNKSPLLLEMNPVHKKIPVLI

I.triloba_GSTU MAGGEEVVLLDSYASMFGMRVKVALGEKGIQYVHKEEDLKNKSPLLLEMNPVHKKIPVLI

V.vinifera_GSTU --MADEIILLDFWPSMFGMRVKVALAEKGLEYEFREEDLRNKSPLLLEMNPVHKKIPVLI

******::**.***::* :**** *******:************

D.fullonum_GSTU HNGRPVCESLVEVQYIDEVWKDKSPLLPSHPYQRAQARFWADYVDKKIYDAGRKIWTTNG

C.florida_GSTU HNGKPVCESLIIVQYIDEVWKHKAPLLPSEPYPRAQARFWADYIDKKIYDAGKKIWTTKG

I.trifida_GSTU HNGKPVCESLIIVQYIDEVWKDKSPLLPSHPYNRAQARFWADFVDKKIYDCGRRIWATKG

I.triloba_GSTU HNGKPVCESLIIVQYIDEVWKDKSPLLPSHPYNRAQARFWADFVDKKIYDCGRRIWATKG

V.vinifera_GSTU HNGKPICESLIIVQYIDEVWKDKSPLLPSDPYQRAQARFWADYIDKKIFELGRKIWTTKG

***:*:****: *********.*:*****.** *********::****:: *::**:*:*

D.fullonum_GSTU EEQETAKNELIDCLKVLEGELGDKPYFGGESFGFVDIALLPFSTWFYAYETLGNFSIEAE

C.florida_GSTU KEQEAAKEEFIECLKVLEGVLGDNPYFGGEKFGFVDVALVPFYSWFYAYETLGNFSIESE

I.trifida_GSTU EEQEAAKKELIDCLKLLEGELGDKPFFGGESFGFVDVALIPFYTWFYTYEKHGNFSIEAH

I.triloba_GSTU EEQEAAKKELIDCLKLLEGELGDKPFFGGESFGFVDVALIPFYTWFYTYEKHGNFSIEAH

V.vinifera_GSTU EDQEAAKKEFIECLKLLEGELGEKPYFGGENFGFVDVALVTFSCWFYAYETFGNFSIEAE

::**:**:*:*:***:*** **::*:****.*****:**:.* ***:**. ******:.

D.fullonum_GSTU CPKLIAWVNRCKEKETVSKSLSDPHKVYEFVLRLRKKLGI-

C.florida_GSTU CPQLVAWAKRCMEKESVSKSLPDPHKIYDFVLGMKKKFGLD

I.trifida_GSTU CPKLVEWAKRCMQKDSVSTSLADPHKIHEFVIQLKKRLGIE

I.triloba_GSTU CPKLVEWAKRCMQKDSVSTSLADPHKIHEFVIQLKKRLGID

V.vinifera_GSTU CPKLIAWTKRCMERESVSSSLADPHKVHGFVVVLRKKFGIE

**:*: *.:** ::::**.**.****:: **: ::*::*:


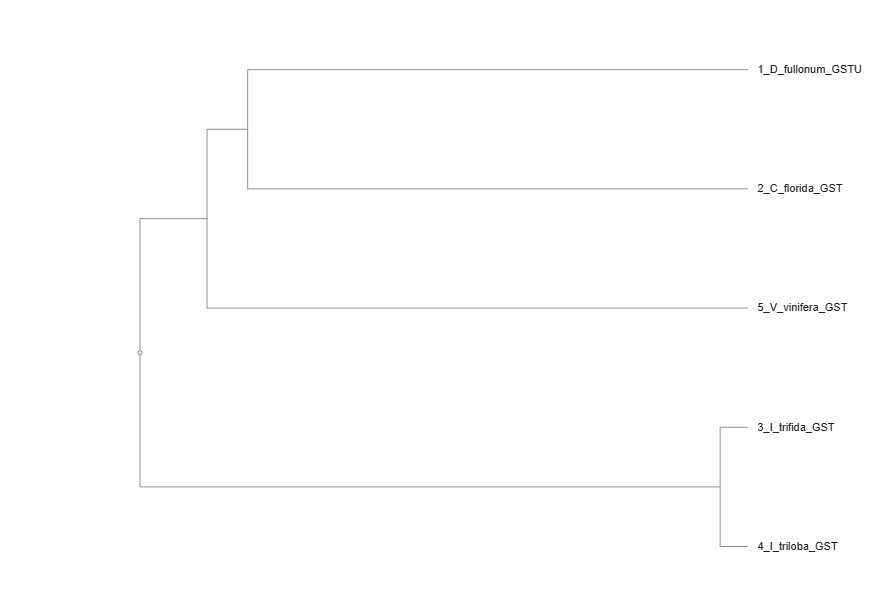


# DfHDA19

Domain: cd10005

D.fullonum_HDA19 METGGNSLPSGPDGVKRKVFYFYDPEVGNYYYGQGHPMKPHRIRMTHALLAHYGLLQHMH

C.florida_HDA19 MEIGGNSLPSGPDGVKRKVCYFYDPEVGNYYYGQGHPMKPHRIRMTHALLAHYGLLQHMN

S.oleosum_HDA19 MDTGGNSLPSGPDGVKRKICYFYDPEVGNYYYGQGHPMKPHRIRMTHALLAHYGLLQHMQ

E.grandis_HDA19 MDTGGNSLPSGPDGVKRKVCYFYDPEVGNYYYGQGHPMKPHRIRMTHALLAHYGLLQHMQ

A.eriantha_HDA19 MEAGGNSLPSGPDGVKRKVFYFYDPEVGNYYYGQGHPMKPHRIRMTHALLAHYGLLQHMH

*: ***************: ***************************************:

D.fullonum_HDA19 VVKPVPARDKDLQRFHADDYVGFLRGITPETQQDQLRQLKRFNVGEDCPVFDGLYSFCQT

C.florida_HDA19 VLKPCPARDRDLCRFHADDYVAFLRGITPETQQDQLRQLKRFNVGEDCPVFDGLYSFCQT

S.oleosum_HDA19 VLKPIPARDRDLCRFHADDYVSFLRSITPETQQDQLRQLKRFNVGEDCPVFDGLYSFCQT

E.grandis_HDA19 VLKPVPARDRDLCRFHADDYVAFLRSITPETQQDQLRQLKRFNVGEDCPVFDGLHSFCQT

A.eriantha_HDA19 VVKPALARDRDLCRFHADDYVAFLRSITPETQQDQLRQLKRFNVGEDCPVFDGLYSFCQT

*:** ***:** ********.***.****************************:*****

D.fullonum_HDA19 YAGGSVGGAVKLNHGSCDIAINWAGGLHHAKKCEASGFCYVNDIVLAILELLKVHEXXRV

C.florida_HDA19 YAGGSVGGAVKLNHG-CDIAINWAGGLHHAKKCEASGFCYVNDIVLAILELLKMHE--RV

S.oleosum_HDA19 YAGGSVGGAVKLNHGLCDIAINWAGGLHHAKKCEASGFCYVNDIVLAILELLKQHE--RV

E.grandis_HDA19 YAGGSVGGAVKLNHGLCDIAINWAGGLHHAKKCEASGFCYVNDIVLGILELLKQHE--RV

A.eriantha_HDA19 YAGGSVGGAVKLNHG-CDIAINWSGGLHHAKKCEASGFCYVNDIVLAILELLKVHE--RV

*************** *******:**********************.****** ** **

D.fullonum_HDA19 LYVDIDIHHGDGVEEAFYTTDRVMTVSFHKFGDYFPGTGDIRDIGHAKGKYYSLNVPLDD

C.florida_HDA19 LYVDIDIHHGDGVEEAFYTTDRVMTVSFHKFGDYFPGTGDVRDIGYGKGKYYSLNVPLDD

S.oleosum_HDA19 LYVDIDIHHGDGVEEAFYTTDRVMTVSFHKFGDYFPGTGDIRDVGYGKGKYYSLNVPLDD

E.grandis_HDA19 LYVDIDIHHGDGVEEAFYTTDRVMTVSFHKFGDYFPGTGDIRDIGYGKGKYYSLNVPLDD

A.eriantha_HDA19 LYVDIDIHHGDGVEEAFYTTDRVMTVSFHKFGDYFPGTGDIRDVGYAKGKYYSLNVPLDD

****************************************:**:*:.*************

D.fullonum_HDA19 GIDDESYQSLFKPIMGKVMDVFRPGAVVLQCGADSLSGDRLGCFNLSIKGHAECVKYMRS

C.florida_HDA19 GIDDESYQSLFKPIMGKVMEVFRPGAVVLQCGADSLSGDRLGCFNLSIKGHAECVKYMRS

S.oleosum_HDA19 GIDDESYHSLFKPIIGKVMEVFKPGAVVLQCGADSLSGDRLGCFNLSIKGHAECVRYMRS

E.grandis_HDA19 GIDDESYHSLFKPIIGKVMEVFKPGAVVLQCGADSLSGDRLGCFNLSIKGHAECVRYMRS

A.eriantha_HDA19 GIDDESYQSLFKPIMGKVMEVFKPGAVVLQCGADSLSGDRLGCFNLSIKGHAECVRYMRS

*******:******:****:**:********************************:****

D.fullonum_HDA19 FNVPILLLGGGGYTIRNVARCWCYETGVALGAELDDKMPQHEYYEYFGPDYTLHVAPSNM

C.florida_HDA19 FNVPILLLGGGGYTIRNVARCWCYETGVALGLEVDDKMPQHEYYEYFGPDYTLHVAPSNM

S.oleosum_HDA19 FNVPVLLLGGGGYTIRNVARCWCYETGVALGLEVEDKMPQHEYYEYFGPDYTLHVAPSNM

E.grandis_HDA19 FNVPVLLLGGGGYTIRNVARCWCYETGVALGLEVDDKMPQHEYYEYFGPDYTLHVAPSNM

A.eriantha_HDA19 FNVPLLLVGGGGYTIRNVARCWCYETGVALGVELEDKMPQHEYYEYFGPDYTLHVAPSNM

****:**:*********************** *::*************************

D.fullonum_HDA19 ENKNSRQQLDEIRSGLLQNLSQLQHAPSVQFQERPPDTEFAEEDEDQDDEDERWDPDSYM

C.florida_HDA19 ENKNSRPLLEDIRVKLLDNLSKLQHAPSVQFQERPPETELPEADEDQDDEDERWDPESDM

S.oleosum_HDA19 ENKNSRQLLEEIRSKLLDNLSKLQHAPSVQFQERPPDTELPEADEDQEDPDERWDPDSDM

E.grandis_HDA19 ENKNSRQLLEDIRSKLLENLSKLQHAPSVPFQERPPDTELPEADEDQEDPDERWDPDSDM

A.eriantha_HDA19 ENKNSRQQLDEIRAKLLDYLSKLQHAPSVQFQERPPDTELPEVDEDQEDEDGRWDHDCDM

****** *::** **: **:******* ******:**:.* ****:* * *** :. *

D.fullonum_HDA19 DVDAECKPLSSRV-------------------------------------------LSFK

C.florida_HDA19 DVDDNCKPLPGRVKREF-PEPEHNDAEDLKVP-ERARETDAPPAETATLKVSNPDSMSMD

S.oleosum_HDA19 DVDEDRKPLPSRVKRELIVEPEVKEQDSQKASIDHGRALDAAQEDNASLKVSDMNSMITD

E.grandis_HDA19 DVDEDRKPLPSRVKRELIVEPEVKDQDSQKASIDHGRGLDTTQEDNASIKVSDMNSMITD

A.eriantha_HDA19 DGVDDCKHVSGRVKREY-LEPK--DTEDQKEK-EYARETDATFMETTFLKASYSGPMSID

* : * :..** : .

D.fullonum_HDA19 -------------------------

C.florida_HDA19 EPEIKVEQENSNKPPDQ--PAEMSS

S.oleosum_HDA19 EQSVKMEQDNMNKPSEQIFPK----

E.grandis_HDA19 EQSVKMEQDNVNKPSEQIFPK----

A.eriantha_HDA19 EPQIKLEQDNLNKPSDQ--PVEMN-


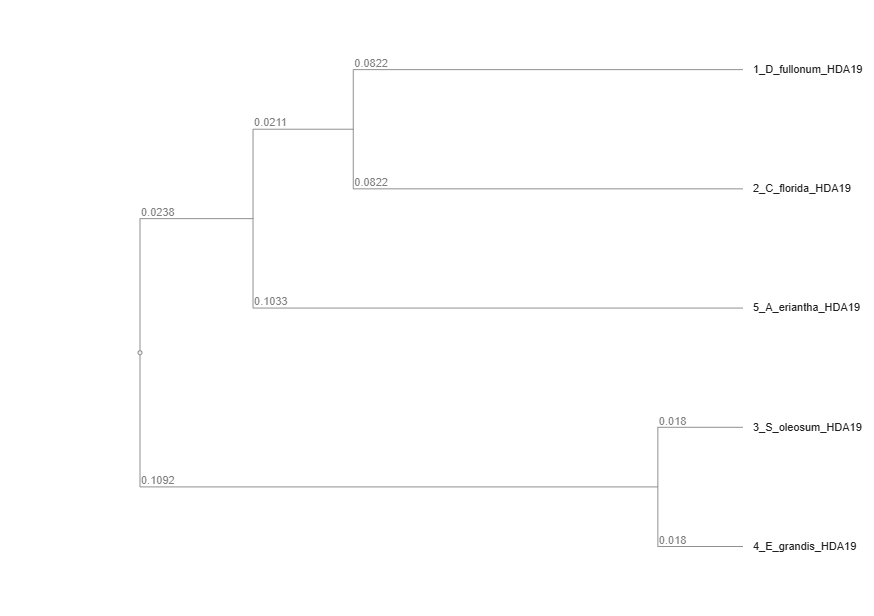


## Comparison across A. thaliana HDAs:

- HDA5; TAIR Protein ID: AT5G61060.2
- HDA19; TAIR Protein ID: AT4G38130.1
- HDA6; TAIR Protein ID: AT5G63110.1
- HDA9; TAIR Protein ID: AT3G44680.1
- HDA7; TAIR Protein ID: AT5G35600.1
- HDA17; TAIR Protein ID: AT3G44490.1
- HDA8; TAIR Protein ID: AT1G08460.1
- HDA5; TAIR Protein ID: AT5G61060.2

#
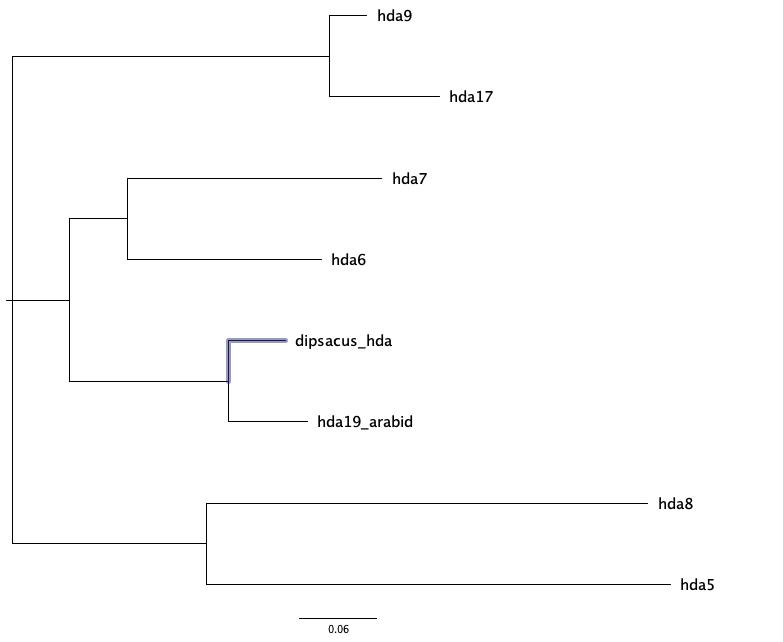


# DfMSD1

Domain: PLN02471

C.arabica_MSD1 MALRTLVTRKALGNSVAFRQQLR---GLQTYTLPDLPYDYGAIEPAISGEIMQLHHQKHH

V.vinifera_MSD1 MALRTLITRKSLGLGLGVSQSVR---GLQTVSLPDLPYDYGALEPAISGEIMKLHHQKHH

H.annuus_MSD1 MALRALTNVKTLGR--LRHQQIR---GLQTFTLPDLSYDYGALEPAISGEIMQLHHQKHH

E.salsugiueum_MSD1 MAIRSVATRKTLAGLKETSSRLLGFRGIQTFTLPDLPYDYSALEPAISGEIMQLHHQKHH

B.napus_MSD1 MAIRSVLSTRTLSGLKETSSKLLGFRGIQTFTLPDLPYDYSALEPAISGEIMQIHHQKHH

D.fullonum_MSD1 MAHRMFATTKPGRVGREIHQQLR---GLQSATLPDLPYDYEDLEPVIDGETMRFNHLKHH

** * . . :. . : *:*: :****.*** :**.*.** *:::* ***

C.arabica_MSD1 QTYVTNFNKALEQLDDAINKGDAPTVVKLQSAIKFNGGGHINHSIFWKNLAPIREGGGEP

V.vinifera_MSD1 QTYITNYNKALEQLHEAMEKGDSPTVVKLQGAIKFNSGGHGNHSIFWKNLTPVHEGGGEP

H.annuus_MSD1 QTYITNYNKALEQLDDAIAKGDASTAVKLQSAIKFNGGGHVNHSIFWKNLAPTREGGGEP

E.salsugiueum_MSD1 QTYVTNYNNALEQLDQAVNKGDASTVVKLQSAIKFNGGGHVNHSIFWKNLAPVNQGGGEP

B.napus_MSD1 QTYVTNYNNALEQLDQAVNKGDASTVVKLHSAIKFNGGGHVNHSIFWKNLAPVNQGGGEP

D.fullonum_MSD1 QTYVTNYNRALEHLEDAINEGDASTVLKLTSPINFNGGGHVNHSIFWKNLAPIREGGGEP

***:**:*.***:*.:*: :**:.*.:** ..*:**.*** *********:* .:*****

C.arabica_MSD1 PKGSLGWAIDNHFGSLEALVQKMNADGAGLQGSGWVWLGLDKELKRLVVETTANQDPLVT

V.vinifera_MSD1 PKGSLGWAIDTHFGSMEALVAKINSEGAAVQGSGWVWLGLDKDLKKLVVETTANQDPLVT

H.annuus_MSD1 PHGSLGWAIDQHFGSMEKLVAKMNAEGAAVQGSGWVWLAVDKELKRLVVETTANQDPLVT

E.salsugiueum_MSD1 PKGALGGAIDTHFGSLEGLVKKMNAEGAALQGSGWVWLGLDKELKKLVVDTTANQDPLVT

B.napus_MSD1 PKGALGGAIDTHFGSLEGLVKKMSAEGAALQGSGWVWLGLDKELKKLVVDTTANQDPLVT

D.fullonum_MSD1 PIGTLDGAINAHFGSMANLIQNMTVQGAALQGSGWVWLGLDKDFKKLVVETTLNQDPLYS

* *:*. **: ****: *: ::. :**.:********.:**::*:***:** ***** :

C.arabica_MSD1 KGSSLVPLLGIDVWEHAYYLQYKNVRPDYLKNIWKVINWKYASDIYEKECP

V.vinifera_MSD1 KGPNLVPLLGIDVWEHAYYLQYRNVRPDYLKNVWKVIDWKYASEVYEKECP

H.annuus_MSD1 KGASLVPLVGIDVWEHAYYLQYKNVRPDYLKNIWKVINWKYASEVYEKECP

E.salsugiueum_MSD1 KGASLVPLVGIDVWEHAYYLQYKNVRPDYLKNVWKVINWKYASEVYEKECK

B.napus_MSD1 KGASLVPLVGIDVWEHAYYLQYKNVRPDYLKNVWKVINWKYASEVYESECK

D.fullonum_MSD1 KGPSLVPLIGIDVWEHAYYLKVYMLHTFVIAMLKFY---------------

**..****:***********: ::. : :


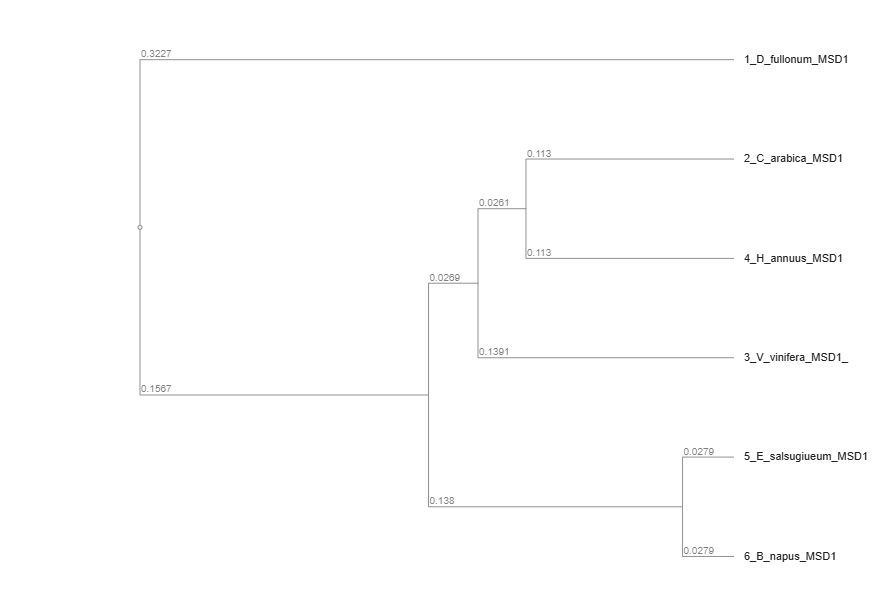


# DfNCED6

Domain: cd03016

V.vinifera_NCED6 ---------MQASPGFSSTAITSAPESPAPTKLHPPPSKLLIQTSNPPLPLKLIAPLT--

C.florida_NCED6 MQASYHFCTTQTTTKLRNQTKPHTPIRKFHLNAPSYTCKILINPSKNTVPRKLTTPAQSE

D.fullonum_NCED6 --------MSTHTMQYFHPFHNPPPTNNTRKYTPQITCKIFVDPGKSTTHRKLSIPPLPE

H.syriacus_NCED6 -------------------MQLQPSFPLLQPRSPLLTCKFLVNPSKKTIRPNPHPSRYPK

A.thaliana_NCED6 -------MQHSLRSDLLPTKTSPRSHLLPQPKNANISRRILINPFKIPTLPDLTSPVPSP

. . :::::. : . . .

V.vinifera_NCED6 -------------SPLATKWPSHLNPLQKLAASALDMVECFLIHQLDNKHTLPKPLDPAV

C.florida_NCED6 TNSNPLLKPRISGAPLEPHHSLVLNPIQKLAVSALDFVERSLILELEKKQKLNRTVDPAV

D.fullonum_NCED6 LFRHP-----------KPEYPNGLNRLQQLAASALDMVEKSFILELEKPHKLTKTTDPTV

H.syriacus_NCED6 VERGG----SVLVPPRELNYPAHLNPFQKLAAAALDKIETSMIMPLEKNHVLPKTVDPTV

A.thaliana_NCED6 VKLKP--------------TYPNLNLLQKLAATMLDKIESSIVIPMEQNRPLPKPTDPAV

** :*:**.: ** :* :: ::: : * :. **:*

V.vinifera_NCED6 QLVGNFAPVRECPVQHDLQVLGQLPPALHGVYLRNGANPMLSPAGGHHLFDGDGMIHAVT

C.florida_NCED6 QLEGNFAPVCECPVLHGLEVVGQIPAGLRGVYLRNGANPLFSPINGHHLFDGDGMIHAVT

D.fullonum_NCED6 QLAGNFAPVPECPVEHGLEVVGHIPKDLNGVYLRNGANPMFTPTGGHHLFDGDGMIHAVK

H.syriacus_NCED6 QISGNFAPVGECPVRHGLKVVGHIPACLHGVYVRNGANPMFVPSGGHHLFDGDGMIHAIG

A.thaliana_NCED6 QLSGNFAPVNECPVQNGLEVVGQIPSCLKGVYIRNGANPMFPPLAGHHLFDGDGMIHAVS

*: ****** **** :.*:*:*::* *.***:******:: * *************:

V.vinifera_NCED6 LGPGNRASYSCRFTRTSRLVQEAALGRPLFPKPIGELHGHSGIARLALFYARAAVGLVDG

C.florida_NCED6 FGSPNRPSYSCRFTRTSRLVQESALGRPLFPKPIGELHGHLGLARLSLFFARASIGLVDS

D.fullonum_NCED6 MEPGDHASYCCRFTRTNRLVQESKLGRAIFPKPIGELHGYSGLARLALFYARAAVGLVDP

H.syriacus_NCED6 LGPGNEASYSCRYTRTSRLIQEARLGRCMFPKPIGELHGHLGLARLGLFMARSGLGLVDG

A.thaliana_NCED6 IGFDNQVSYSCRYTKTNRLVQETALGRSVFPKPIGELHGHSGLARLALFTARAGIGLVDG

: :. **.**:*:*.**:**: *** :**********: *:***.** **:.:****

V.vinifera_NCED6 SRGTGVANAGLVYFNGRLLAMSEDDLPYHVKINGDGDLETTGRFDFSGQMDRPMIAHPKV

C.florida_NCED6 SRGTGVANAGLICFNGRVLAMSEDDLPYNVRITADGDLETIGRFDFNGQLENSMIAHPKV

D.fullonum_NCED6 SHGIGVANAGLVYFNGRLLAMSEDDLPYNVRVTGDGDLETIGQFDFDGQLNCPMIAHPKL

H.syriacus_NCED6 SHGTGVANAGLVYFNGRLLAMSEDDLPYHVKINGDADLETVGRFDFDDQIDCPLIAHPKL

A.thaliana_NCED6 TRGMGVANAGVVFFNGRLLAMSEDDLPYQVKIDGQGDLETIGRFGFDDQIDSSVIAHPKV

::* ******:: ****:**********:*:: .:.**** *:*.*..*:: .:*****:

V.vinifera_NCED6 DPITGELFSLSYNVVKKPYLKYYKFGTCGEKSREVSISLQQPTIIHDFALTETSVVIPDH

C.florida_NCED6 DPVTGDLYSLSYNVLKKPYLKYLKFDACGRKSRDVPISVQQPTMIHDFAITESHVIIPDH

D.fullonum_NCED6 DPVTHELYTLSYNVVSKPYLKFFKFDTSDQKSRDVPISLEQPTMIHDFAITESHVIIPDQ

H.syriacus_NCED6 DPVTGDLHTLNYNVLKKPYLKYFKLDKFGRKSRDLHVDIEQATIVHDFAITENFVIIPDH

A.thaliana_NCED6 DATTGDLHTLSYNVLKKPHLRYLKFNTCGKKTRDVEITLPEPTMIHDFAITENFVVIPDQ

*. * :*.:*.***:.**:*:: *:. ..*:*:: : : :.*::****:**. *:***:

V.vinifera_NCED6 QVVFKLSEMFRGGSPVIHDPNKISRFGVLPRNDPDGSRIQWIDVPDCFCFHLWNAWDE-R

C.florida_NCED6 QVVFKLSEMVRGGSPVIHDPNKTSRFGVLPKEDDNESGIRWVEVPDCFCFHLWNAWEE-Y

D.fullonum_NCED6 QVVFKLSEMIRGKSPVIHDPNKMSRFGVLSKTDADGSSIHWIDVPDCFCFHLWNSWDE-R

H.syriacus_NCED6 QMVFKLSEMIRGGSPVVYNKKKTSRFGVLSQNDAVGSGIRWIDVPDCFCFHLWNAWEEDN

A.thaliana_NCED6 QMVFKLSEMIRGGSPVIYVKEKMARFGVLSKQDLTGSDINWVDVPDCFCFHLWNAWEE-R

*:*******.** ***:: :* :*****.: * * *.*::***********:*:*

V.vinifera_NCED6 SSSGDRIVVVIGSCMSPADSIFTERVDPLRSELSEIRLNLTTGGSSRRVIVAGMNLEAGQ

C.florida_NCED6 NKHGDPIIVVIGSCMTPPDSIFKEGDSPIRSELSEIRLNLNTGGSTRRVIVAGLNLEAGQ

D.fullonum_NCED6 TENGETEIVVIGSCMTPADSIFTEQSDPLRAELSEIRLNLNTGESTRRVIVSGMNLEVGH

H.syriacus_NCED6 TETGDKTVVVIGSCMNPPDSIFNESGDTLRSELSEIRMNLRTGESTRRVIVSGMNLEAGQ

A.thaliana_NCED6 TEEGDPVIVVIGSCMSPPDTIFSESGEPTRVELSEIRLNMRTKESNRKVIVTGVNLEAGH

.. *: :*******.*.*:**.* .. * ******:*: * *.*:***:*:***.*:

V.vinifera_NCED6 VDKRRLGRKTRYIYLAIAEPWPKCSGMAKVDLVTGEVTKMMYGEGRFGGEACFVG----A

C.florida_NCED6 VDKRRLGRKSRYVYLAVAEPWPKCSGIAKVDLATGEVTHFLYGAGRFGGEPCLVA----D

D.fullonum_NCED6 VNSNRLGRKNAICIFSNC------------------------------------------

H.syriacus_NCED6 VNGQYLGRKTRFVYLAIADPWPKCSGIAKVDLRTGEVTEFMYGACRFGGEPFFVPENKRM

A.thaliana_NCED6 INRSYVGRKSQFVYIAIADPWPKCSGIAKVDIQNGTVSEFNYGPSRFGGEPCFVP-----

:: :***. :: .

V.vinifera_NCED6 EEG-GEGEGWLMSIVRDEKRERSELIVVAADDIKQVASVRLPTRVPYGFHGTFVDSQQLR

C.florida_NCED6 ETSEDEDKGWMMSFVRDEKRDRSELVIADASNMKQVASVKLPTRVPYGFHGTFVSSQCLK

D.fullonum_NCED6 ------------------------------------------------------------

H.syriacus_NCED6 NRDNDEDEGYIMGLVRDEEKETSEMAIVKAGNMKQVGTVRLPTRVPYGFHGTFVSQQDLH

A.thaliana_NCED6 EGEGEEDKGYVMGFVRDEEKDESEFVVVDATDMKQVAAVRLPERVPYGFHGTFVSENQLK

V.vinifera_NCED6 GQRVC

C.florida_NCED6 EQALY

D.fullonum_NCED6 -----

H.syriacus_NCED6 RQVV-

A.thaliana_NCED6 EQVF-


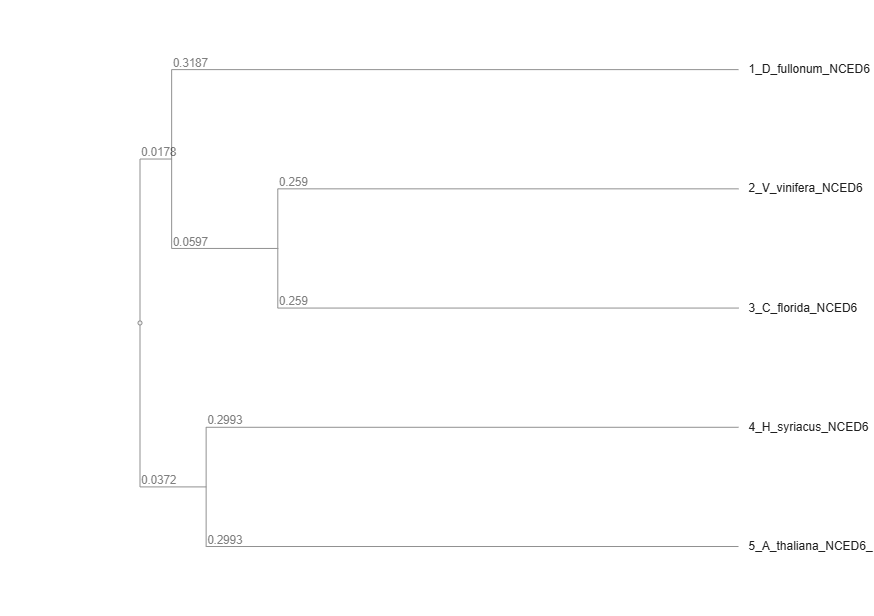


# DfNIA1

Domain: PLN02252

C.arabica_NIA1 MAASVENRQFHHLEPALPRFRAG----PNHRSDSPVRTFN----IHPNKPTVNTVTSNVH

O.europaea_NIA1 MAASVENRQFNRPS-FKPTPPAG----QHHRSDSPIRGCN----FPSNSSSEFKKINGST

L.sativa_NIA1 MAASVENRQFRHELGVSAAGVVRSFSPNHRRSDSPIRGYN----YPAAAREFMTPKKLPP

D.fullonum_NIA1 ------------------------------------------------------------

P.granatum_NIA1 MAASVDKRQFSHLEPGMNGVVRSFKPGPNHASRTDSSPLRNRTTTGFTRKKVAFFNDNAE

V.riparia_NIA1 MAASVDNRQFSRLESSLNGVVRSFKTGSNHRSDSPVRGGG----CNFPSNSEFTRPKKSV

C.arabica_NIA1 GDYPSSDDEEENDENGYEDAIKKGKAELENSVLDARDEGTADNWIERNPSMVRLTGKHPF

O.europaea_NIA1 VFDYSSSEDDDDDENEYVNAIKKAKSELEP-SVDSRDEGTADNWVERNPSMVRLTGKHPF

L.sativa_NIA1 ETYDSSEDEE--DEGHYRDAIKKSNSELESSVFDSRDQGTADQWIERNPSMVRLTGKHPF

D.fullonum_NIA1 ------------------------------------------------------------

P.granatum_NIA1 EEEETSSDDELSELETYRDLIKKGNSELEPSTLDARDEGTADHWIERHPTMVRLTGKHPF

V.riparia_NIA1 AAVEDDSSSDDENEPDWKDLVRKGNSELEPSVLDSRDEGTADNWIQRNPSMVRLTGKHPF

C.arabica_NIA1 NAEPPLTRLMHHGFITPVPLHYVRNHGPVPTATWDGWTVEVCGLVKRPMKFSMEKLVNEF

O.europaea_NIA1 NAEAPLTRLMHHGFITPVPLHYVRNHGYVPRARWDEWTVEITGLVKRPMKLTMQQLVDEF

L.sativa_NIA1 NSEPPLTKLMQHGFITPAPLHYVRNHGPVPNATWEDWTVEICGLVKRPARFSMAQLVNEF

D.fullonum_NIA1 ------------------------------------------------------------

P.granatum_NIA1 NAEAPLGHLMHHGFITPVPLHYVRNHGPVPKAAWQDWTVEVTGLVKRPTRFTMDQLVNDF

V.riparia_NIA1 NSEAPLNRLMHHGFITPVPLHYVRNHGAVPKGSWDNWTVEVSGLVKRPARFTMDQLVNEF

C.arabica_NIA1 PYREFPATLVCAGNRRKEQNMTKQTIGFNWGAAAVSTSVWRGVPLRAILKRCGILSRKNG

O.europaea_NIA1 PSREFPVTLVCAGNRRKEQNMVKQTIGFNWGAAGISTSVWCGVPLHAILKQCGIYSRKKG

L.sativa_NIA1 PSREFPVSLVCAGNRRKEQNLTKQTIGFNWGAAGISTSVWKGVPLVSILKRCGIYSRKKG

D.fullonum_NIA1 ------------------------------------------------------------

P.granatum_NIA1 LAREFPTTLVCAGNRRKEQNMVKQTIGFNWGPAGVSTSVWRGVPLREVLKRVGIMSRKSG

V.riparia_NIA1 PTREFPVTLVCAGNRRKEQNMVKQTIGFNWGAAGVSTSVWRGVRLRDVLKRCGIMSRKQG

C.arabica_NIA1 ALNVCFEGAENLPGGGGSKYGTCVKKGVAMDASRDIILAYMQNGELLTPDHGFPVRMIIP

O.europaea_NIA1 GLNVCFEGAEDLPGGGGAKYGTSIKKEFAMDPSRDIILAYMQNGEKLAPDHGFPVRMIIP

L.sativa_NIA1 ALNVCFEGAEDLPGGGGSKYGTSIKIEVAMDPARDIILAYMQNGEKLLPDHGFPVRMIIP

D.fullonum_NIA1 ------------------------------------------------------------

P.granatum_NIA1 AMNVCFEGAEDLPGGGGSKYATSITKEWAMDPARDIILAYMQNGEPLSPDHGFPVRMIIP

V.riparia_NIA1 GLNVCFEGAEDLPGGGGSKYGTSIKKEIAMDPSRDIILAYMQNGERLLPDHGFPVRMIIP

C.arabica_NIA1 GFIGGRMVKWLSRIIVTTQESDSYYHFKDNRVLPSHVDAELANSEAWWYKPEFIINELNI

O.europaea_NIA1 GFIGGRMVKWLKRIIVTTEESNNYYHYKDNRVLPSHVDAELANAEAWWYKPEYIINELNI

L.sativa_NIA1 GCIGGRMVKWLKRIIVTTPESENYYHFKDNRVLPSHVDAELANSEGWWYKPEYIINELNI

D.fullonum_NIA1 ------------------------------------------------------------

P.granatum_NIA1 GCIGGRMVKWLKRIIVTTKESDNYYHYKDNRVLPSHVDAELANAEAWWYKPEYIINELNI

V.riparia_NIA1 GFIGGRMVKWLKRIIVTTQESDSYYHYKDNRVLPSHVDAELANAEAWWYKPECIINELNI

C.arabica_NIA1 NSIITTPCHDEILPINSWTTQRPYTLRGYAYSGGGKKVTRVEVTMDGGDTWHVCAVDHPE

O.europaea_NIA1 NSVITTPYHEEILPINSWTTQRPYTLRGYAYSGGGKKVTRVEVTMDGGETWQIAALDHTE

L.sativa_NIA1 NSVITTPCHEEILPINSWTTQRPYTLRGYAYSGGGKKVTRVEVTMDGGETWNVCTLDHKE

D.fullonum_NIA1 ------------------------------------------------------------

P.granatum_NIA1 NSVITTPSHEEVLPINSWTTQRPYTLKGYAYSGGGKKVTRVEVTMDGGETWQVCDLDQPE

V.riparia_NIA1 NSVITTPCHEEILPINSWTTQRPYTLKGYAYSGGGKKVTRVEVTMDGGETWQVCRLDHPE

C.arabica_NIA1 KPTKYGKYWCWCFWSLDVEVLDLLGAKEIAVRAWDESTNTQPEKLIWNVMGMMNNCWFRV

O.europaea_NIA1 KPNKYGKYWCWCFWSLEVEVLVLLAAKEVAVRAWDQSLNTQPQNLIWNVMGMMNNCWFRV

L.sativa_NIA1 KPTRYAKYWCWCFWSLEVEVLDLLGAKEIAVRAWDETLNTQPDKLIWNLMGMMNNCWFRV

D.fullonum_NIA1 ------------------------------------------------------------

P.granatum_NIA1 KPNKYGKYWCWCFWSLEVEVLNLLGTKEIAVRAWDETLNTQPEKLIWNVMGMMNNCWFRV

V.riparia_NIA1 KPNKYGKYWCWCFWSLEVEVLDLIGAKEIAVRAWDETLNTQPEKLIWNVMGMMNNCWFRV

C.arabica_NIA1 KTNVCKPHKGEIGIVFEHPTQPGNQSGGWMAKEKHLEKSSDSNQ-TLKKSVSSPFMNTSS

O.europaea_NIA1 KTNVCKPHKGEIGIAFEHPTQPGNQSGGWMAKERHLEKSSEANQ-TLKKSVSSPFMNTSS

L.sativa_NIA1 KTNMCKPHKGEIGIVFEHPTQPGNQSGGWMAREKHLEISSELAHPTLKKSVSSPFMNTTS

D.fullonum_NIA1 ------------------------------------------------------------

P.granatum_NIA1 KTNVCKRHKGEIGIVFEHPTQPGNQSGGWMAKEKHLELSTDAGQ-TLKKSVSTPFMNTSS

V.riparia_NIA1 KTNVCKRHRGEIGIVFEHPTLPGNQSGGWMAREKHLVQSSDANS-TLKKSVSSPFMNTSF

C.arabica_NIA1 KMFSMSEVKKHNSADSAWIIVHGHVYDTTRFLKDHPGGSDSILINAGTDCTEEFEAIHSD

O.europaea_NIA1 KMYSMSEVKKHNSSDSAWIIVHGHVYDCTRFLKDHPGGTDSILINSGTDCTEEFDAIHSD

L.sativa_NIA1 LTFTMSEVKKHNSADSAWIVVHGHIYDCSSFLKDHPGGSDSILINAGTDCTEEFDAIHSD

D.fullonum_NIA1 ------------------------------------------------------------

P.granatum_NIA1 KTYPMSEVKKHNSADSAWIIVHGHIYDCTKFLKDHPGGADSILINAGTDCTEEFDAIHSD

V.riparia_NIA1 QMYSMSEVKKHNSADSTWIVVHGHVYDCTRFLKDHPGGTDSILINAGTDCTEEFDAIHSD

C.arabica_NIA1 KAKKLLEDFRIGELITSGYTSDSSTSSPNNTVHGASN--ASHLAPITEIAPARSIALISG

O.europaea_NIA1 KAKKLLEDYRIGELITTGYTSDSSTSSPNNSVHGPSGG-GLYLAPIKEIAPARNVALIPR

L.sativa_NIA1 KAKKLLEEYRIGELITTGYSSDSAASSPNTSVHGATNYMTSHLATIKEIAPTRNVALVPR

D.fullonum_NIA1 ----------------------------------------MHLAPITELAPARSVALVPR

P.granatum_NIA1 KAKKLLEDYRIGELVTSGYTSDQSTASSPNNSVHGSGNLSHLAPIKEVVAPIRSVALVPR

V.riparia_NIA1 KAKKLLEDYRIGELMTTGYTSDSS-ASSPNTSVHGAS-NLTHLAPIKEVTPLRSVALVPR

. ::* *.:**:.

C.arabica_NIA1 QRIPCKLVSKTSISHDVRKFRFALPSEEQVLGLPIGKHIFICATVDEKLCMRAYTPTSGV

O.europaea_NIA1 EKIPCKLVSKTSISHDVRLFRFALPAEDQVLGLPVGKHIFLCATIEDKLCMRAYTPTSGV

L.sativa_NIA1 EKIPCKLVSKTSVSHDVRLFRFALPSSEQVLGLPVGKHIFVCATVDDKLCMRAYTPTSTI

D.fullonum_NIA1 EKIPCKLVAKTSISHDVRLFRFELPMKDQVLGLPVGKHIFLCATINEKLCMRAYTPTSSV

P.granatum_NIA1 EKIPCKLVAKTSISHDVRVFRFALPSEDQVLGLPVGKHIFLCATINDKLCMRAYTPTSSI

V.riparia_NIA1 EKIPCKLVSKDSISHDVRRFRFALPSEDQVLGLPVGKHIFLCAAIDGKLCMRAYTPTSNI

::****** * *:***** *** ** .:******:*****:**::: **********: :

C.arabica_NIA1 EEVGYFELVVKIYFKGVHPRFPNGGVMSQYLDSLSLGSFLEIKGPLGHIEYKGKGNFLVH

O.europaea_NIA1 DTVGYFELVVKIYFKGIHPKFPNGGLMSQHLVSLQVGSFLDVKGPLGHIEYTGKGNFLVH

L.sativa_NIA1 DEVGYFELLVKIYFKGVEPKFPNGGIMSQYLESMEIGSSLEIKGPLGHIEYMGRGTFSVH

D.fullonum_NIA1 DEVGYFELVVKIYFKDENPRFPNGGQMSQYLDTLALNSTIDVKGPLGHIEYKGRGNFMVN

P.granatum_NIA1 DEVGYFDLVVKVYFKNVHPKFPNGGMMSQYIDSLPLGSTLDVKGPLGHIEYTGRGNFTVH

V.riparia_NIA1 DEVGFFELVVKIYFKGVHPKFPNGGLMSQYLDSLPLGATLDVKGPLGHIEYTGRGNFLVH

: **.*:*:**:***. .*:***** ***:: :: :.: :::********* *:*.* *:

C.arabica_NIA1 GKHKFAKKLAMLAGGTGITPIYQVMQAILKDSEDDTEMFVVYANRTEDDILLRDELDAWA

O.europaea_NIA1 GKHKFAKKLALLAGGTGITPVYQVMQAILKDPEDETEMFVVYANRTEDDILLKDELDEWA

L.sativa_NIA1 GKQKFARKLAMFAGGTGITPIYQVMQAILKDPEDDTEMYVVYANRTEDDILLREELDAWA

D.fullonum_NIA1 GKQKFAKKLAMLAGGTGITPIYQVMQAIH-------------------------------

P.granatum_NIA1 GKPKFAKKLAMLAGGTGITPIYQVCQAILKDPEDQTEMYVVYANRTEDDILLRDELDEWA

V.riparia_NIA1 GKPKFAKKLAMIAGGSGITPIYQIIQAVLKDPEDDTEMYVVYANRTEDDILLWEELDAWA

** ***:***::***:****:**: **:

C.arabica_NIA1 EKYPERVKVWYVVEKSIKEGWNYSLGFVTESILREHVPLASETTLALACGPPPMIQFAIN

O.europaea_NIA1 EKYPDRVKVWYVVQESIKEGWRYSLGFITESILREHIPEASKTTLALACGPPPMIQFAIN

L.sativa_NIA1 DKYGDRVKVWYVVAKSIREGWKYSEGFITEDIMREHIPEVSEDTLALACGPPPMIQFAIN

D.fullonum_NIA1 ------------------------------------------------------------

P.granatum_NIA1 KKH-DRFKVWYVVQESIREGWQYGLGFITEDILREHIPEGSADTLALACGPPPMIQFAVQ

V.riparia_NIA1 AKH-ERLKVWYVVGESIRKGWKYSLGFITESILREHIPSASGDTLALACGPPPMIQFAVQ

C.arabica_NIA1 PNLEKMGYDIKDSLLIF

O.europaea_NIA1 PNLEKMGYDIKNSLLVF

L.sativa_NIA1 PNLEKMGYDIKNSLLVF

D.fullonum_NIA1 -----------------

P.granatum_NIA1 PNLEKMNYDVKDSLLVF

V.riparia_NIA1 PNLEKLGYDIKNSLLVF


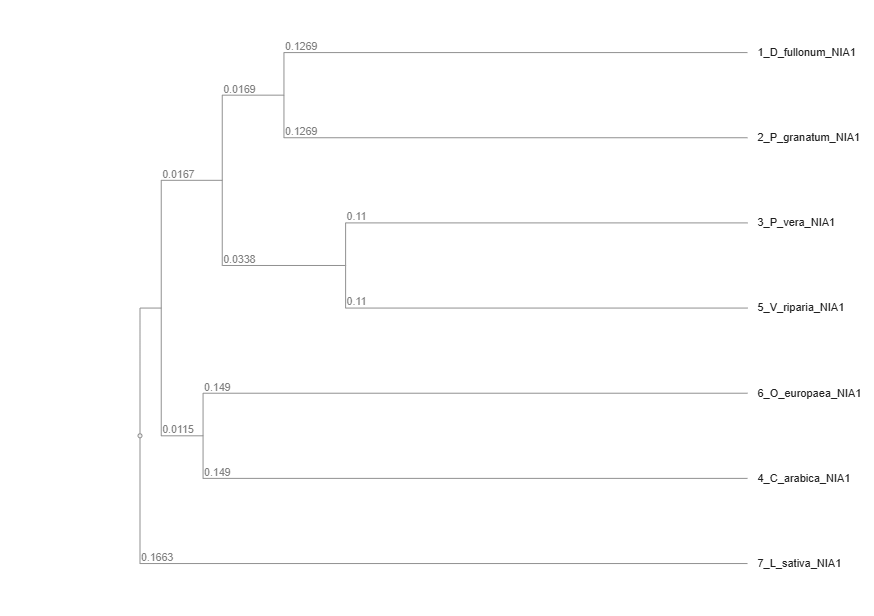


# DfPRX1

Domain: cd03016

D.fullonum_PRX1 MPGLTIGDTVPNLDVQTTHGKINLHQYVANSYTILFSHPGDFTPVCTTELGAMAVYADKF

H.annuus_PRX1 MPGLTIGDSLPNLQVDTTHGKINLHDYVGDSFTIIFSHPGDFTPVCTTELGAMAAYADKF

L.ferocissimum_PRX1 MPGLTIGDDLPNLEVETTHGKMKLHDYVGDSYTILFSHPGDFTPVCTTELGMMAAYASKF

H.annuus_PRX1 MPGLTIGDSLPNLQVDTTHGKINLHDYVGDSFTIIFSHPGDFTPVCTTELGAMAAYADKF

S.asiatica_PRX1 MPGLTIGDTLPDLEVDTTHGKIKLHDYVADSFTIIFSHPGDFTPVCTTELGMMAAYADKF

******** :*:*:*:*****::**:**.:*:**:**************** **.**.**

D.fullonum_PRX1 AQRGAKLLGLSCDDIQSHVEWIKDIEAYNSGHKVTYPIIADPNREIIKQLNMVDPDEKDS

H.annuus_PRX1 AQRGVKLLGLSCDDVQSHKEWIKDIEAYNKGKKVTYPIAADPNREIIKQLNMVDPDEKDA

L.ferocissimum_PRX1 AERGVRLLGFSCDDVQSHKEWIKDIEAYNKGHKVTYPIIADPKRELIKQLNMVDPDETDS

H.annuus_PRX1 AQRGVKLLGLSCDDVQSHKEWIKDIEAYN-GKKVTYPIAADPNREIIKQLNMVDPDEKDA

S.asiatica_PRX1 AERGVKLLGLSCDDVESHNTWIQDIEAYNKGAKVTYPIAADPNRHIIEQLNMVDPDEKDS

*:**.:***:****::** **:****** * ****** ***:*.:*:*********.*:

D.fullonum_PRX1 SRSQVPSRALHIVGPDKKIKLSFLYPASTGRNMDEVVRVLDSLQKAAKHKVATPVNWRQG

H.annuus_PRX1 SGQNLPSRALHIVGPDKKIKLSFLYPASTGRNMDEVVRALDSLIKASQHKIATPVNWKEG

L.ferocissimum_PRX1 TGQKVPSRALHIVGPDKKIKLSFLYPASTGRNMDEVLRVVDSLQKAAKHKVATPVNWKPG

H.annuus_PRX1 SGQNLPSRALHIVGPDKKIKLSFLYPASTGRNMDEVVRALDSLIKASQHKIATPVNWKEG

S.asiatica_PRX1 SGGHVPSRALHIVGPDKKIKLSFLYPASTGRNMDEVVRVLDSLQRACKHKIATPANWKPG

: ::*******************************:*.:*** :*.:**:***.**: *

D.fullonum_PRX1 EQVVISPNVSGDQAKEMFPQGYKTVDMPSKKEFLRFT---

H.annuus_PRX1 EPVVIAPSVSNDEARKMFPKGFQTVDLPSNKDYLRFTSV-

L.ferocissimum_PRX1 DPVVISPSVSNEQAKEMFPQGYDTANLPSGKDYLRFTNV-

H.annuus_PRX1 EPVVIAPSVSNDEARKMFPKGFQTVDLPSNKDYLRFTSV-

S.asiatica_PRX1 DKVVISPSVSSDEAKSMFPQGYESADLPSKKDYLRFTNVD

: ***:*.**.::*:.***:*:.:.::** *::****

#
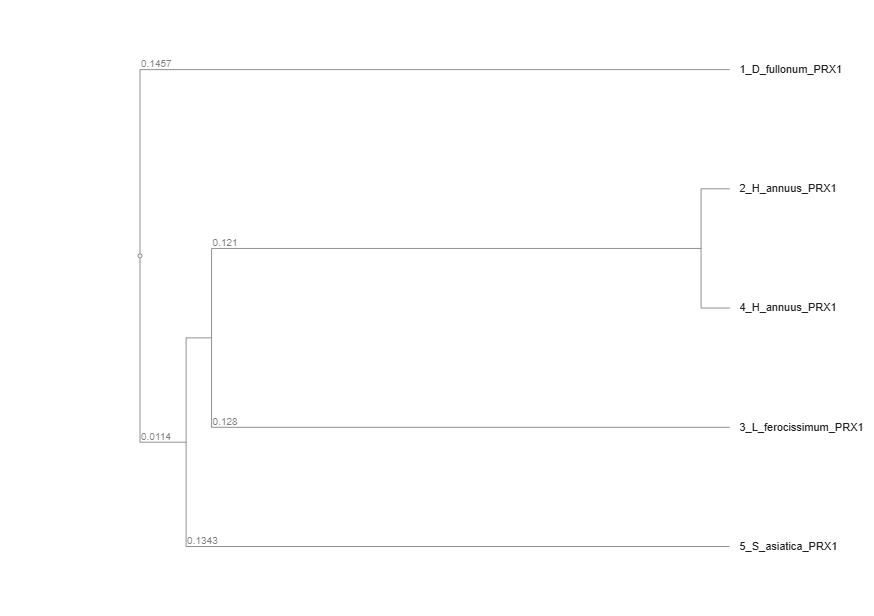


# DfPIF1

Domain: cd11445

D.zibethinus_PIF1 --------MNHCVPDFEMEEDYS---ILSSSSLTRPKKPSMPE-DEIMEL

H.umbratica_PIF1 --------MHHCVPDFEMGDDYS---IPSSSSLTRPKKPPMPE-DEIMEL

H.syriacus_PIF1 --------MNHCVPDSEMEDDDS---IPSSS-LTRSKKPSIPE-DEVMEL

C.illinoinensis_PIF1 --------MYHCVPDFEMDDDYS---LPSSSGAARPRKSSLPE-DEIMEL

A.eriantha_PIF1 MLGFLLQTMNHCVPDFEMDEDYA---IPTSSGLSRPKKSPMPE-EEIMEL

D.fullonum_PIF1 --------MNQYVPDFGIPDDYS---VRQSSGAMKS--SSTMQQQHVVEL

A.thaliana_PIF1 --------MHHFVPDFDTDDDYVNNHNSSLNHLPRKSITTMGEDDDLMEL

* : *** :* . : .. : :.::**

D.zibethinus_PIF1 LWQNGQVVMQSQNHRSFKKYTPFKFHDADQSAPREIRSSSSLQHQQQQQQ

H.umbratica_PIF1 LWQNGQVVVQSQNQRSFKKSPPFKFHDADQSAPKEIRSSSSHHQHHQQQQ

H.syriacus_PIF1 LWQNGQVVMHSQNQRSTKLSPPFKFQDSNQSAPREIRSSSYHHHHQQQQQ

C.illinoinensis_PIF1 LWQNGQVVMHSQNQRSMRRSPPSKYDN--AVCPADQSANREIRSSQEEQE

A.eriantha_PIF1 LWQNGQVVMQSQNQRSLRRSQIG-----DAVLPQEQSAAR-------QIR

D.fullonum_PIF1 KWQNGPVEMQNQNQNQTS-----------LRRSDDNGIVTGQSNGSKDFR

A.thaliana_PIF1 LWQNGQVVVQNQR-----------------LHTKKPSSSPPKLLPSMDPQ

**** * ::.* . . : .

D.zibethinus_PIF1 QQSITD-HLFMQEDEMASWLHYPLSDAN--FDHDFCADLLYPSSSAAAPA

H.umbratica_PIF1 QQSITD-HLFMQEDEMASWLHYPLNDAN--FDHDFCADLLYPSSSAAAAT

H.syriacus_PIF1 QQLLTENHLFMQEDEMASWLHYPLGDAS--FDREFCADLLYPSS--AAAA

C.illinoinensis_PIF1 SAAHQQQHFFMQEDEMASWLHYPLVDDDPALDHNFCADLLYPTPNQSNNI

A.eriantha_PIF TEEESATQLFMQEDEMASWLHCTIDDDS--FERDLYADLIYQAPCAPAAP

D.fullonum_PIF1 SEEETVNHLFMQEDEMSSWLHYPLDETP---FDSDITDLLYSTPPAPLTA

A.thaliana_PIF1 QQPSSDQNLFIQEDEMTSWLHYPLRDDD------FCSDLLFSAAPTATAT

.:*:*****:**** .: : :**:: .

D.zibethinus_PIF1 VAPSVTSTATTSAPPPLGRVSQVSG----SASAMASASRPPIPPERRNEL

H.umbratica_PIF1 PCITST---ATAAPPPLGRVSQVSASASASAAAAASAPRPAIPPARRNGS

H.syriacus_PIF1 PCVTSVTATTSSAPPPLGTVSQFSAS----------ASRPPVPPSRRNEL

C.illinoinensis_PIF1 TNTNANPVFRTNGVTELRQKVTGGVG----------TSRPPIPPGRRTDL

A.eriantha_PIF1 IPDVCAPEVRIPPPN-------------------AAASRPPIPPAR----

D.fullonum_PIF1 PPSTTPEALPALPPLKPPQSAVTFAP------------RPPVPPMR--HL

A.thaliana_PIF1 ATVSQVTAARPPVSSTNE-------------------SRPPVR-------

**.:

D.zibethinus_PIF1 ESTRIQNFVHFSRHK-----TARAEQSGPSNSKSLVRESTVVDSSDTPAV

H.umbratica_PIF1 EPTRTKNFVHFSRHK-----TATVEQSGPSNSKSVVRESTVVDSSDTPAM

H.syriacus_PIF1 ESSRIQNFVHFSTNK-----ATRAEQSAPSNFKSAVRESTVVDSSDTPTM

C.illinoinensis_PIF1 ADSNVQNFVHFSRPKGKLAAGVDKTRPLSSKSMAVVRESTVVDSSETPVV

A.eriantha_PIF1 ------NFLHFSRPK-------GKMEPGPSNSNKAVRETTVVDSSETPAP

D.fullonum_PIF1 ESQSFRNFAHFSRHS------WRSSEFGASTSMRTVRDCNGIESNETPVM

A.thaliana_PIF1 ------NFMNFSRLRG-DFNNGRGGESGPLLSKAVVRESTQVSPSATPSA

.* :** . **: . :... **

D.zibethinus_PIF1 APESAASQVMPSNTEEASGGNNNNACANMSAAAVVNTQSAAVSVGASKDN

H.umbratica_PIF1 APESRASQAVPSNTEAASGGNNNNACATVSAAAVASRQSAGVSVGATKDN

H.syriacus_PIF1 APESGASWAMPSNTEGASGGNNNTDCANMSVAAGVSG-------SASKDN

C.illinoinensis_PIF1 GPESWTRSAAEASDFG---NRGCPTMSCDAVAGTSSAGAGGK-------E

A.eriantha_PIF1 --ESRVSHATAQVSAANAGICAMSGGGAVAVTSSAAGGGG--------RE

D.fullonum_PIF1 EPEPRVTVVDNTAQVSGGNSPNGGG-------------------------

A.thaliana_PIF1 AASESGLTRRTDGTDSSAVAGGGAYNRKG--------------------K

.

D.zibethinus_PIF1 LATCEVTVTSSPG--GSSASAEPTAQKAAPAEDRKRKG-REPDDAECHS-

H.umbratica_PIF1 LATCEVTVTSSPG--GSSASAEQATQKAAPAEDRKRKG-IEPDDAECHS-

H.syriacus_PIF1 LDSFEVTVTSSPG--GSSASAEPTAQKAAPAEDRKRQG-RELDDAECHR-

C.illinoinensis_PIF1 MMSCEVTLTSSSG--CSSGSASAEPARKPPVEYNRKRKGIEAEDADCHC-

A.eriantha_PIF1 AGTCELTVTSSPGGSGASVSANAEAVYKPMATEGRKRKGLETDDTECQSE

D.fullonum_PIF1 IVLGKLPMTSSPGSGGSASGSAELSKMATPAKEDLKRKAIEIDDTECHS-

A.thaliana_PIF1 AVAMTAPAIEITGTSSSVVSKSEIEPEKTNVDDRKRKEREATTTDETES-

. . .* : . . :: : .

D.zibethinus_PIF1 EDAEFESTD-TKKQTRGSISTKRSRAAEVHNLSERRRRDRINEKMRALQE

H.umbratica_PIF1 EDAEFESAD-IKKQTRGSTSTKRSRAAEVHNLSERRRRDRINEKMRALQE

H.syriacus_PIF1 EDFEFESPE-TKKQTRGSTSTKRSRAAEVHNLSERRRRDRINEKMRALQE

C.illinoinensis_PIF1 EDVEFESAA-ANKQVHGSTSTKRSRAAEVHNLSERRRRDRINEKMKALQE

A.eriantha_PIF1 QDVEFESPD-AKKQVRGSTSTKRSRAAEVHNLSERRRRDRINEKMRALQE

D.fullonum_PIF1 EGVEFESADGKKKISHGSRSTKRSRAAEVHNLSERRRRDRINEKMKALQE

A.thaliana_PIF1 ------RSEETKQARVSTTSTKRSRAAEVHNLSERKRRDRINERMKALQE

. :: .: ****************:*******:*:****

D.zibethinus_PIF1 LIPRCNKSQSDKASMLDEAIEYLKSLQLQVQMMSMGCGMVPMMFPGVQQY

H.umbratica_PIF1 LIPRCNKS--DKASMLDEAIEYLKSLQLQVQMMSMGCGMVPMMFPGVQQY

H.syriacus_PIF1 LIPRCNKS--DKASMLDEAIEYLKSLQLQVQMMSMGCGMVPMMFPGVQQY

C.illinoinensis_PIF1 LIPRCNKFQSDKASMLDEAIEYLKSLQLQVQMMSMGCGMVPMMFPGVQQY

A.eriantha_PIF1 LIPRCNKS--DKASMLDEAIEYLKSLQLQVQMMSMGCSMVPMMFPGVQQY

D.fullonum_PIF1 LIPGCNKA--DKASMLDEAIEYLKALQLQVQVMSMGCNMVPVMFPGAPQY

A.thaliana_PIF1 LIPRCNKS--DKASMLDEAIEYMKSLQLQIQMMSMGCGMMPMMYPGMQQY

*** *** ************:*:****:*:*****.*:*:*:** **

D.zibethinus_PIF1 MPT-----IGMGIGMGMGMDMGI----SRPMMPFPNVLAGL-ALPTPAAA

H.umbratica_PIF1 MPT-----MGMGIGMGMGMDMGI----SRPMLPFPNVLAGS-VLPTPAAA

H.syriacus_PIF1 MPT-----MGMGTGMGMDIGR--------PMTPFPNVLAGS-PLPTPASA

C.illinoinensis_PIF1 MPA-----MGMGIGMGMGMDMG------RSMIPFPNMLAGS-ALQTSAAA

A.eriantha_PIF1 MPQ-----MGMGMGMGMGMEMGMN----RPMMPFPSVLAGS-ALQTQAAA

D.fullonum_PIF1 MPMGMGMGMGMGMGMGMGMGMGMGMSMNRPVVPFSAVPGGA-AMQNPAAA

A.thaliana_PIF1 MPH-------MAMGMGMNQPIPPP-----SFMPFPNMLAAQRPLPTQTHM

** *. ****. .. **. : .. : . :

D.zibethinus_PIF1 AHLGPRFPIPAFHMPPPVPAP-------NNQPDATINQLGMQNTNQ-PRV

H.umbratica_PIF1 AHLGPRFPLPAFHMPPPVPPPDPSRIQPNNQSEAMLNTLGMQNPTQ-PRA

H.syriacus_PIF1 AHLGPRFPMPAFHMAPPAPPTDPSRIQPNNQSDTMLNPLSMQNPNQQLCV

C.illinoinensis_PIF1 AHLGPRYPIPAFHMP-PVPAPDPSRIQATNRPQQMLHSIGTQHTSQ-PRF

A.eriantha_PIF1 AHMGPPFPMQAFHMP-PVPLPDPSRPQATGQSDLMLFSMGTQNPNQ-PRM

D.fullonum_PIF1 VAQIP------------------------------------------PRF

A.thaliana_PIF1 AGSGPQYPVHASDPS----------------RVFVPNQQYDPTSGQPQYP

. *

D.zibethinus_PIF1 LNFADPYQQYIG-LHQIQLPPPLSRAMAQTSSNKPSNSREAENLENQPPE

H.umbratica_PIF1 PNFADPYQQYLG-LQQMQLPPLQSQGMAQPSSRKPSSSRGAENLKNHPSG

H.syriacus_PIF1 PKFSDPYQQYYIGLHPMQLPSPQNQTMAQPSSSRPGTSKGADNLENHPSG

C.illinoinensis_PIF1 PNFVDPYQQYVA-THQMQLSRPQNQALAQPSTSKPNTIKGPEIPDNHQSG

A.eriantha_PIF1 PNFADPYQQYLG-LHQMQLPLPQNQAMMQPSMSKPSSSRDVETRETPPSG

D.fullonum_PIF1 RVTPPPFPMQPP-LDLSRVPPTKPDSISTMCYSRPHTSTFTSESSTSTTY

A.thaliana_PIF1 AGYTDPYQQFRG-LHPTQPPQFQNQATSYPSSSRVSSSKESEDHGNHTTG

*: . : . . : . . . .

D.zibethinus_PIF1 SERGLDRRISDKKTKPSLGTWGTVVR

H.umbratica_PIF1 EMTR----------------------

H.syriacus_PIF1 DMGR----------------------

C.illinoinensis_PIF1 T-------------------------

A.eriantha_PIF1 --------------------------

D.fullonum_PIF1 YRQ-----------------------

A.thaliana_PIF1 --------------------------


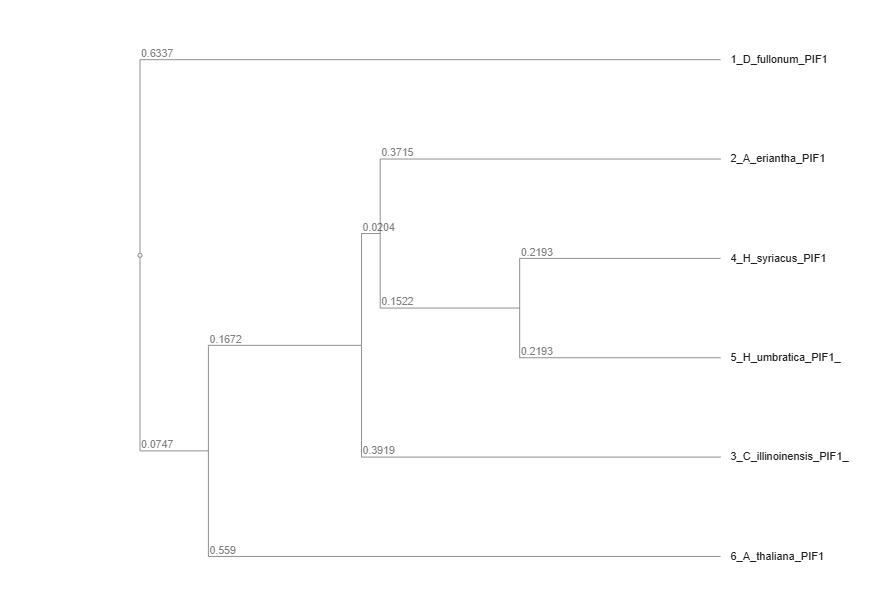


# DfPYL4

Domain: cd07821

D.fullonum_PYL4 -----MHTNQQRSSLLLDRINT-----SSPCLKQSPQKSPTTLHRRAPLHCSTPVPESVE

C.florida_PYL4 -----MPANPQKSSLLLHRINYATTTNTSTCHKQSQQQQPTTLLRRNPLTCTTPVPDSVG

M.esculenta_PYL4 -----MPSNPHKSSLLLHRINTTPTNSTTAASTSACQT------RWSPLSCATLIPETVA

H.brasiliensis_PYL4 -----MPSNRPKSSLLQHRINTTPTNSTIAATTAACQR------RRSPLSCATPVPETVA

M.indica_PYL4 MISQKMPSNPPKSSPLLHRINNKATATAITA-NMLCQK---GFQKRFPLTSATPVPDTVA

P.vera_PYL4 MISQKMPSNPPKSSILLQRINNTATTTATTA-NRLCQK---GFQKRFPLTSATPVPDTIA

* :* :** * .*** : .. . * : ** .:* :*:::

D.fullonum_PYL4 RYHIHAVAPNQCCSAVIQQISAPVSTVWSVVRRFDNPQAYKHFVKSCQVTVGEGNVGTLR

C.florida_PYL4 RYHIHTVSPNQCCSAVVQQIAAPLSTVWPVVRRFDNPQAYKHFVKSCHVIFGEGDVGTLR

M.esculenta_PYL4 RYHNHVVGPNQCCSFEVQQIAAPVSIVWSVVRRFDNPQAYKHFVKSCHVIVGDGNVGTLR

H.brasiliensis_PYL4 RYHNHVVGPNQCCSVEVQQIAAPVSTVWSVVRRFDNPQAYKHFVKSCHVIVGDGDVGTLR

M.indica_PYL4 RYHVHAVGPNQCCSSVIQQIAAPVSTIWSVVRRFDNPQAYKHFVKSCHVIDGDGDVGTVR

P.vera_PYL4 RYHVHAVGPNQCCSSVVQLIAAPVSTVWSVVRRFDNPQAYKHFVKSCHVINGDGDVGTLR

*** *.*.****** :* *:**:* :*.******************:* *:*:***:*

D.fullonum_PYL4 EVHVISGLPAASSTERLEILDDERHVISFSVVGGDHRLANYRSVTTLHPSPTGNGTVVIE

C.florida_PYL4 EIHVISGLPAGHSTERLEILDDERHVISFSVVGGDHRLANYRSVTTLHPTPAGDGTVVVE

M.esculenta_PYL4 EVHVVSGLPAANSTERLEILDDERHVISFSVVGGDHRLANYRSVTTLHPSPAGNGTVLVE

H.brasiliensis_PYL4 EVHVVSGLPAANSTERLEILDDERHVISFSVVGGDHRLANYRSVTTLHPSLAGNGTVVVE

M.indica_PYL4 EVHVISGLPAANSTERLEILDDESHVISFSVVGGDHRLSNYKSVTTLHPSPSGNGTVVIE

P.vera_PYL4 EVHVISGLPAANSTERLEILDDERHVISFSVVGGDHRLANYKSVTTLHPSPAGNGTVVIE

*:**:*****. *********** **************:**:*******: :*:***::*

D.fullonum_PYL4 SYVVDTPQGNTKEETCVFVDTIVRCNLQSLTQIAENLAPRNT----

C.florida_PYL4 SYVVDIPPGNTKEDAFAFIDTIVRCNLQSLAQIADNSGRRNSL---

M.esculenta_PYL4 SYVVDIPPGNTKEDTCVFVDTIVRCNLQSLTQIAENLARNNTSSS-

H.brasiliensis_PYL4 SYVVDIPPGNTKEDTCVFVDTIVRCNLQSLTQIAENLAGSNKSSS-

M.indica_PYL4 SYAVDVPPGNTKEDTCVFVDTIVRCNLQSLSQIAENLARRNLNNAT

P.vera_PYL4 SYVVDVPPGNTKEDTCVFVDTIVRCNLQSLAQIADNLARRNLN---

**.** * *****:: .*:***********:***:* . *


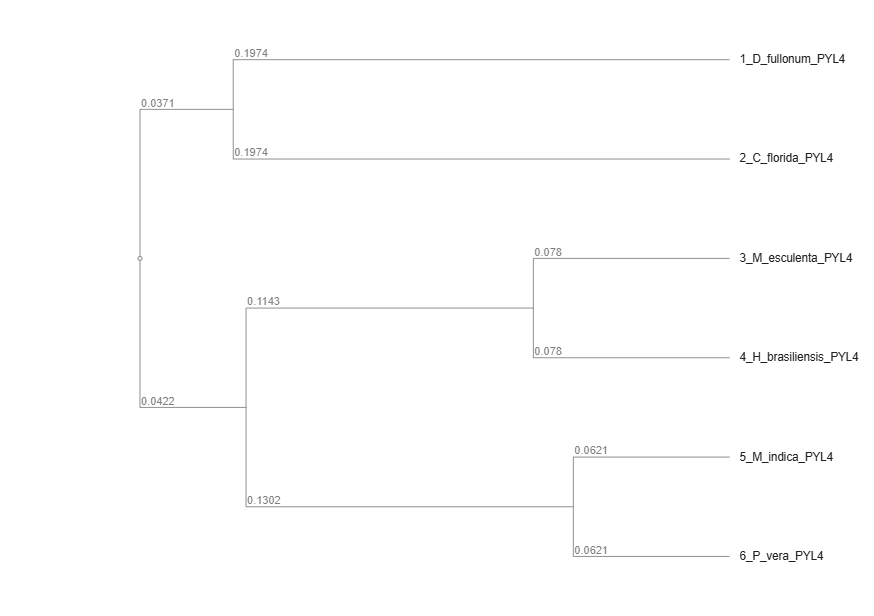


# DfPYL8

Domain: cd07821

D.fullonum_PYL8 MNGSRYSVMEGEYIRKHHRHEIGENQCSNTLVRHIKAPVHLVWSLVRRFDQPQKYKPFVS

A.eriantha_PYL8 MNSNGFSSMEIEYIRKHHRHHLTENQCSSALVKHIKAPVHLVWSLVRRFDQPQKYKPFIS

V.vinifera_PYL8 MNGNGLSSMESEYIRRHHRHEPAENQCSSALVKHIKAPVPLVWSLVRRFDQPQKYKPFIS

L.ferocissimum_PYL8 MNANGYSVMEKEYIRKHHIHEVKENRCSSFLVKHIRAPVHLVWSLVRRFDQPQKYKPFIS

R.vialii_PYL8 MNANGFSSVEKEYIRKHHRHYPAENQCSSTLVKHIKAPVHLVWSLVRRFDQPQKYKPFIS

L.barbarum_PYL8 MNANGYSVMEKEYIRKHHIHEVKENRCSSFLVKHIRAPVHLVWSLVRRFDQPQTYKPFIS

**.. * :* ****:** * **:**. **:**:*** *************.****:*

D.fullonum_PYL8 RCVVQGNLEIGSLREVDVKSGLPATTSTERLELLDDDEHILSVRIVGGDHRLRNYSSVIS

A.eriantha_PYL8 RCVVQGNLEIGSLREVDVKSGLPATTSTERLELLDDDEHILSIRIVGGDHRLRNYSSIIS

V.vinifera_PYL8 RCVVQGNLEIGSLREVDVKSGLPATTSTERLELLDDDEHILSMRIIGGDHRLRNYSSIIS

L.ferocissimum_PYL8 RCIVQGDLEIGSLREVDVKSGLPATTSTERLELLDDEEHILSVRIVGGDHRLRNYSSIIS

R.vialii_PYL8 RCVVKGNLEIGSLREVDVKSGLPATTSTERLEILDDEEHILSIRIVGGDHRLRNYSSILS

L.barbarum_PYL8 RCIVQGDLEIGSLREVDVKSGLPATTSTERLELLDDEEHILSVRIVGGDHRLRNYSSIIS

**:*:*:*************************:***:*****:**:***********::*

D.fullonum_PYL8 VHPEVIDGRPGTLVIESFVVDVPEGNTKDETCYFVEALIKCNLKSLADVSERLAVQDRTE

A.eriantha_PYL8 VHPEVIDGRPGTLVIESFVVDVPEGNTKDETCYFVEALIKCNLKSLADVSERLALQDRTE

V.vinifera_PYL8 LHPEIIDGRPGTMVIESYVVDVPEGNTKDETCYFVEALIKCNLKSLADVSERLAVQDRTE

L.ferocissimum_PYL8 VHPEVIDGRPGTLVIESFVVDVPEGNTKDETCYFVEALIKCNLKSLADVSERLAVQDRTE

R.vialii_PYL8 VHPEVIDGRPGTLVIESFVVDVPDGNTQDETCYFVEALIKCNLKSLADVSERLAVQDRTE

L.barbarum_PYL8 VHPEVIDGRPGTLVIESFVVDVPEGNTKDETCYFVEALIKCNLKSLADVSERLAVQDRTE

:***:*******:****:*****:***:**************************:*****

D.fullonum_PYL8 PIDRM

A.eriantha_PYL8 PIDRM

V.vinifera_PYL8 PIDRM

L.ferocissimum_PYL8 PIDQV

R.vialii_PYL8 PIDRM

L.barbarum_PYL8 PIDQV

***::


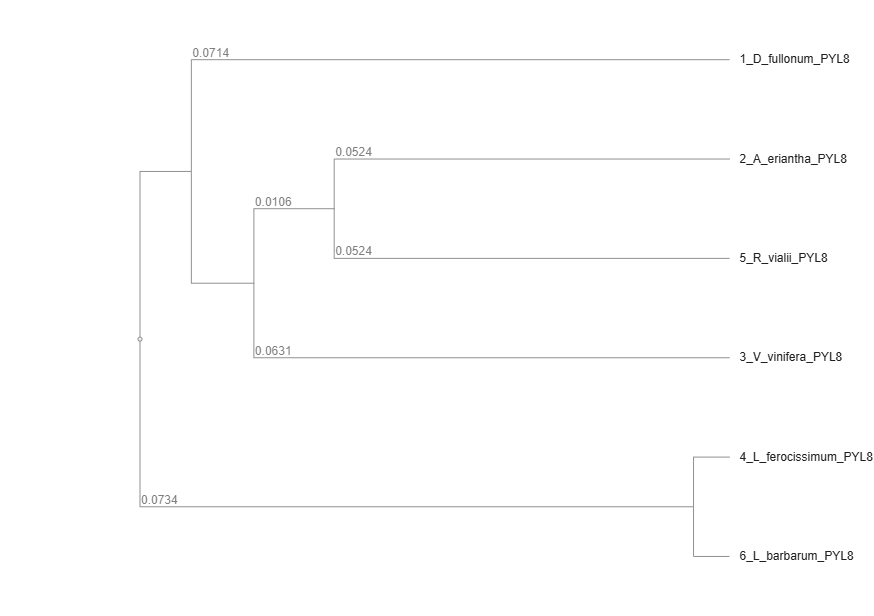


# DfPYL9

Domain: cd07821

D.lotus_PYL9 ---------------MEIQYIQRHHKHEPKENQCTSVLFKHIKAPTDLVWTLVRRFDQPQ

A.thaliana_PYL9 MMDGVEGGTAMYGGLETVQYVRTHHQHLCRENQCTSALVKHIKAPLHLVWSLVRRFDQPQ

C.sinensis_PYL9 ---------------MEAQYIRRYHKHEPKENQCTSEFVKHIKAPVDLVWSLVRRFDQPQ

N.attenuata_PYL9 ---------------MEAQFIARYHSHQPSDHQCSSSIVKHIKAPVDIVWSLVRRFDQPQ

N.sylvestris_PYL9 ---------------MEAQFIARYHSHQPSDHQCSSSIVKHIKAPVDIVWSLVRRFDQPQ

D.fullonum_PYL9 -------------MEVEAQYLSAHHRHEPKDYQCTSYVVKHIKAPVDIVWSLVRRFDKPQ

*:: :* * : **:* ..****** .:**:******:**

D.lotus_PYL9 KYKPFVSRCIVQGDLMIGSLREVNVKSGLPATTSTERLELLDDNEHILGIRIIGGDHRLK

A.thaliana_PYL9 KYKPFVSRCTVIGDPEIGSLREVNVKSGLPATTSTERLELLDDEEHILGIKIIGGDHRLK

C.sinensis_PYL9 RYKPFVSRCVMQGDLNIGSLRQVNVKSGLPATTSTERLELLNDEEHILGIRIIGGDHRLK

N.attenuata_PYL9 KYKPFVSRCTVKGDLRIGSVREVNVKSGLPATTSTERLELLDDEEHILGIRIVGGDHRLK

N.sylvestris_PYL9 KYKPFVSRCTVKGDLRIGSVREVNVKSGLPATTSTERLELLDDEEHILGIRIVGGDHRLK

D.fullonum_PYL9 KYKPFVRGCIVQGDLKIGSVRKVNVKTGLPATTSTERLELLDDEERILCTRIVGGDHRLK

:***** * : ** ***:*:****:**************:*:*:** :*:*******

D.lotus_PYL9 NYSSIITVHPEVIDGRPGTLVIESFLVDVPDGNTKDETCYFVKALINCNLKSLADVSERM

A.thaliana_PYL9 NYSSILTVHPEIIEGRAGTMVIESFVVDVPQGNTKDETCYFVEALIRCNLKSLADVSERL

C.sinensis_PYL9 NYSSIITVHPEMIDGRPGTLVIESFVVDVPEGNTTDETCYFVKALLNCNLKSLADVSERM

N.attenuata_PYL9 NYSSVITVHPEILDGTPGTLVIESFMVDVPEGNTQEETCYFVKALINCNLKSLADVSERM

N.sylvestris_PYL9 NYSSVITVHPEILDGTPGTLVIESFMVDVPEGNTQEETCYFVKALINCNLKSLADVSDRM

D.fullonum_PYL9 NYSSVITVHPEMIDGKPGTTVVESFLVDVPEGNTKEETGYFVKALINCNHKCLSEVSERM

****::*****:::* .** *:***:****:*** :** ***:**:.** *.*::**:*:

D.lotus_PYL9 AMQGWGLINRA----------

A.thaliana_PYL9 ASQDITQ--------------

C.sinensis_PYL9 AMQDRARLVNQI---------

N.attenuata_PYL9 AMHSGGLPISVNWPSSNQIKT

N.sylvestris_PYL9 SMPVEVFPSA-----------

D.fullonum_PYL9 AMNQSRV--------------

:


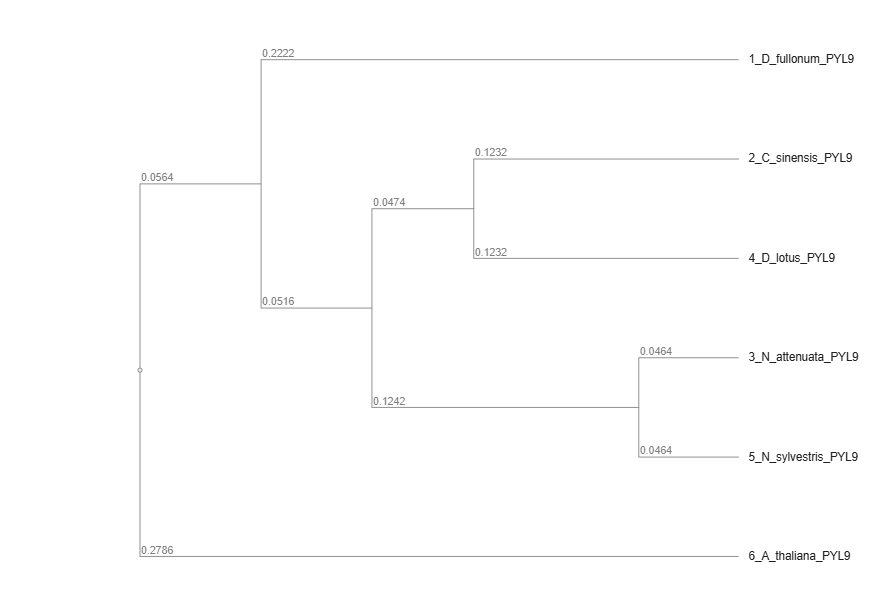


# DfTPL

Domain: pfam17814

D.fullonum_TPL MSSLSRELVFLILQFLDEEKFKETVHKLEQESGFYFNMKYFEEEVHNGNWDEVERYLSGF

S.indicum_TPL MSSLSRELVFLILQFLDEEKFKETVHKLEQESGFFFNMKYFEDEVHNGNWEEVERYLSGF

A.anserina_TPL MSSLSRELVFLILQFLDEEKFKETVHKLEQESGFFFNMKYFEDEVHNGNWDEVEKYLSGF

D.carotasativus_TPL MSSLSRELVFLILQFLDEEKFKETVHKLEQESGFYFNMKYFEEEVHSGNWDEVEKYLSGF

C.sinensis_TPL MSSLSRELVFLILQFLDEEKFKETVHKLEQESGFFFNMRYFEDEVHNGNWDEVERYLSGF

**********************************:***:***:***.***:***:*****

D.fullonum_TPL TKVDDNRYSMKIFFEIRKQKYLEALDKHDRSKAVDILVKDLKVFASFNEELFKEITQLLT

S.indicum_TPL TKVDDNRYSMKIFFEIRKQKYLEALDKHDRSKAVEILVKDLKVFASFNEELFKEITQLLT

A.anserina_TPL TKVDDNRYSMKIFFEIRKQKYLEALDKHDRSKAVDILVKDLKVFATFNEELFKEITQLLT

D.carotasativus_TPL TKVDDNRYSMKIFFEIRKQKYLEALDKNDRSKAVEILVKDLKVFASFNEDLFKEITQLLT

C.sinensis_TPL TKVDDNRYSMKIFFEIRKQKYLEALDKHDRSKAVDILVKDLKVFASFNEELFKEITQLLT

***************************:******:**********:***:**********

D.fullonum_TPL LENFRENEQLSKYGDTKSARAIMLVELKKLIEANPLFRDKLQFPNLKNSRLRTLINQSLN

S.indicum_TPL LENFRENEQLSKYGDTKSARAIMLVELKKLIEANPLFRDKLQFPNLKNSRLRTLINQSLN

A.anserina_TPL LDNFRENEQLSKYGDTKSARAIMLVELKKLIEANPLFRDKLQFPNLKNSRLRTLINQSLN

D.carotasativus_TPL LENFRENEQLSKYGDTKSARAIMLVELKKLIEANPLFREKLQFPNLRNSRLRTLINQSLN

C.sinensis_TPL LENFRENEQLSKYGDTKSARAIMLVELKKLIEANPLFRDKLQFPNLKNSRLRTLINQSLN

*:************************************:*******:*************

D.fullonum_TPL WQHQLCKNPRPNPDIKTLFVDHSCGQPNGARAPSPANNPLL-GSLPKAGVFPPLGAHGPF

S.indicum_TPL WQHQLCKNPRPNPDIKTLFVDHSCGQPNGARAPSPANNPLLGGAVPKPGGFPPLGAHVPF

A.anserina_TPL WQHQLCKNPRPNPDIKTLFVDHSCGQPNGARAPSPANNPLL-GSLPKAGGFPPLGAHGPF

D.carotasativus_TPL WQHQLCKNPRPNPDIKTLFVDHSCAQPNGARAPSPAPNPLL-GPLPKAGGFPPLGAHGPF

C.sinensis_TPL WQHQLCKNPRPNPDIKTLFVDHSCGQPNGARAPSPVNNPLI-GSLPKAGAFPPLGAHGPF

************************.**********. ***: *.:**.* ******* **

D.fullonum_TPL QPTPAPVPTQLAGWMSNPPTVTHPAVS-DGVIGLGGPSITAALKHPRTQPTNPSLDYPSG

S.indicum_TPL QPTPAPVPTPLAGWMSNPPTGTHPAVS-GGPIGLGAPTIPAALKHPRTPPTNPSVDFPSA

A.anserina_TPL QPTPAPVPIPLAGWMSNASTVTHPTVSEGGAIGLGGPSITAALKHPRTPPTNPSVDYPSG

D.carotasativus_TPL QPAAAPVPTPLAGWMSNPPTVAHPAVS-GGAIGLGGPSMPTALKHPRTPQTNPSLDYPSV

C.sinensis_TPL QPTPAAVPAPLAGWMSNPPTVTHPAIS-GGAIGLGGPSISAALKHPRTPPTNPSVDYPSG

**:.*.** *******..* :**::* .* ****.*::.:******* ****:*:**

D.fullonum_TPL DSDHISKRTRPMGISDEISLPVNVMAMSFPGHSHGQAFSPPDDLPKTVARTLNQGSSPMS

S.indicum_TPL DSEHASKRTRPLGISDEVNLPVNVLPVSFPGHAHSQTFSAPDDLPKTVARTLNQGSSPMS

A.anserina_TPL DSDHVSKRTRPMGLSNEVNLPVNILPVSFPGHSHGQALNAPDDLPKNVARNLNQGSSPMS

D.carotasativus_TPL DSDHVAKRTRPLGLSDEVNLPINVLPMSFSGHGHSQAFSAPDDLPKNVARTLNQGSSPMS

C.sinensis_TPL DSDHVSKR-RAMGISDEVNLPVNVLPVSFSGHGHSQALNAPDDLPKTVARTLNQGSAPMS

**:* :** *.:*:*:*:.**:*::.:**.**.*.*::..******.***.*****:***

D.fullonum_TPL MDFHPIQQTLLLVGTNVGDTGLWEVGSRERLVLRNFKVWDLSSCTMPLQAALAKDPVVSV

S.indicum_TPL MDFHPIQQTLLLVGTNVGDLGLWEVGSRERLVQRNFKVWDLGSCTMPLQAALVKDPGVSV

A.anserina_TPL MDFHPLQQTLLLVGTNVGDIGLWEVGSRERLLLRNFKVWDLSSCSMPLQAALVKDPGVSV

D.carotasativus_TPL MDFHPVQQTLLLVGTNVGDIGLWEVGSRERLVLKNFKVWDLSACSVPLQAALVKDPGVSV

C.sinensis_TPL MDFHPIQQTLLLVGTNVGDIALWEVGSRERLVLRNFKVWDLSVCSMPLQAALVKDPSVSV

*****:************* .**********: :*******. *::******.*** ***

D.fullonum_TPL NRVIWSPDGSLFGVAYSRHIVQIYSHHGGGDVRQHLEIDAHIGGVNDLAFSHPNKQLCVI

S.indicum_TPL NRVIWSPDGSLFGVAYSRHLVQIFSYHGNDDVRQHLEIDAHIGGVNDLAFSHPNKQLSVI

A.anserina_TPL NRVIWSPDGSLFGVAYSRHIVQIYSYHGSDDIRQHLEIDAHVGGVNDLAFSHPNKQLCVI

D.carotasativus_TPL NRVIWSPDGSLFGVAYSRHIVQIYSYHGGEDMRQHLEIDAHVGGVNDLAFSHPNKQLCVI

C.sinensis_TPL NRIIWSPDGSLFGVAYSRHIVQIYSYHGADDVRQHLEIDAHVGGVNDLAFSHPNKQLCVI

**:****************:***:*:** *:*********:***************.**

D.fullonum_TPL TCGDDKTIKVWDAATGARQHIFEGHEAPVYSVCPHYKENIQFIFSTALDGKIKAWLYDNL

S.indicum_TPL TCGDDKFIKVWDATTGTKQYTFEGHEAPVYSVCPHHKENIQFIFSTALDGKIKAWLYDNL

A.anserina_TPL TCGDDKTIKVWDAATGSKQYTFEGHEAPVYSVCPHYKENIQFIFSTALDGKIKAWLYDNL

D.carotasativus_TPL TCGDDKTIKVWDATTGAKQYTFEGHEAPVYSVCPHYKENIQFIFSTALDGKIKAWLYDNL

C.sinensis_TPL TCGDDKTIKVWEAATGAKQFTFEGHEAPVYSVCPHYKENIQFIFSTALDGKIKAWLYDNM

****** ****:*:**::*. **************:***********************:

D.fullonum_TPL GSRVDYDAPGRWCTTMAYSADGTRLFSCGTSKEGESHIVEWNESEGAVKRTYQGFRKLSL

S.indicum_TPL GSRVDYDAPGRWCTTMAYSADGTRLFSCGTSKDGESHIVEWNESEGAVKRTYQGFRKRSL

A.anserina_TPL GSRVDYDAPGRWCTTMAYSADGTRLFSCGTSKEGESYIVEWNESEGAVKRTYQGFRKRSF

D.carotasativus_TPL GSRVDYDAPGRWCTTMAYSADGTRLFSCGTSKDGESHIVEWNESEGAVKRTYLGFRKRSL

C.sinensis_TPL GSRVDYDAPGRWCTTMAYSADGTRLFSCGTSKDGESHIVEWNESEGAVKRTYQGLRKRSM

********************************:***:*************** *:** *:

D.fullonum_TPL GVVQFDTTKNRFLAAGDDYTIKFWDMDNVQLLTSIDADGGLPASPRIRFNKDGALLAVSA

S.indicum_TPL GVVQFDTTKNRFLAAGDDFSVKFWDMDNTQLLTSTDADGGLPASPRIRFNKDGTLLAVSA

A.anserina_TPL GVVQFDTTKNRFLAAGDDFSIKFWDMDNVQLLTTVDADGGLPASPRIRFNKDGTLLAVSA

D.carotasativus_TPL GVVQFDTTKNRFLAAGDEFTIKFWDMDSVQILTSIDADGGLPASPRIRFNKDGSLLAVST

C.sinensis_TPL GVVQFDTTKNRFLAAGYDFAIKFWDMDNVQLLTSIDADGNIPASPRIRFNKDGSLLAVSA

**************** ::::******..*:**: ****.:************:*****:

D.fullonum_TPL NENGIKILANSDGLRLLRTFENLSFDASRASE-AVKPSVHPIS-----AAAAS--SAGHA

S.indicum_TPL NENGIKILANTDGLRLLRTFENLAFDASRASE-AAKPTVNPIS-----AAAAS--SAGLT

A.anserina_TPL NENGIKILGNADGVRLLRTFENLSYDASRTSEVVAKPAMNPISVAAAAAAATS--SAGLA

D.carotasativus_TPL NDNGIKILVNSDGLRLLRTIENLSYDASRAPE-ALKPSINTIS-----AAAAAAGTSGLG

C.sinensis_TPL NDNGIKILANSDGLRWLRTFENPSYDAARASENAIKPTINAMS--AAAAAAAT--SAGLG

*:****** *:**:* ***:** ::**:*:.* . **:::.:* ***:: ::*

D.fullonum_TPL DRVASAVAISGVNGDSRNMGDVKPRITEESNDKSKIWKLTEIGEPSQCRSLKLPENLRVT

S.indicum_TPL ERVASVVSISAMNGDARNLGDVKPRITEETNDKSKIWKLSEISEPSQCRSLKLPENLRVT

A.anserina_TPL ERNASAVAISGMNGDARNLGDVKPRITEESNDKSKIWKLTEINEPSQCRSLRLPENMRVT

D.carotasativus_TPL DRVSSAVAISAMNGDARNLGDIKPRITEESNDKSKIWKLTEVTEPSQCRSMKLPENMRVT

C.sinensis_TPL DRAASVVAISAMNGDARNMGDVKPRITEEMNDKSKIWKLTEISDPSQCRSLKLPEHMRAT

:* :*.*:**.:***:**:**:******* *********:*: :******::***::*.*

D.fullonum_TPL KISRLIYTNSGNAILALASNAIHLLWKWQRSDRNSSGKATANVSPQLWQPSSGILMTNDV

S.indicum_TPL KISRLIYTNSGNAILALASNAIHLLWKWQRSERNSSGKATATVPPQLWQPSSGILMTNDV

A.anserina_TPL KISRLIYTNSGNAILALASNAIHLLWKWQRNDRTSVSKATASVSPQLWQPSSGILMTNDV

D.carotasativus_TPL KISRLIYTNSGNAILALASNAVHLLWKWQRSERNSNGKATASVSPQLWQPSSGILMTNDI

C.sinensis_TPL KISRLIYTNSGNAILALASNAIHLLWKWQRSDRNSGGKATANVSPQLWQPSSGILMTNDV

*********************:********.:*.* .****.*.***************:

D.fullonum_TPL TDINPEEAVPCFALSKNDSYVMSASGGKISLFNMMTFKTMTTFMPPPPAATFLAFHPQDN

S.indicum_TPL SDTNPEEAVPCFALSKNDSYVMSASGGKISLFNMMTFKTMTTFMPPPPAATFLAFHPQDN

A.anserina_TPL TDTSPEEAVPCFALSKNDSYVMSASGGKISLFNMMTFKTMTTFMPPPPAATFLAFHPQDN

D.carotasativus_TPL ADSNPEDAVACFALSKNDSYVMSASGGKISLFNMMTFKTMTTFMPPPPAATFLAFHPQDN

C.sinensis_TPL TDTNPEDAVPCFALSKNDSYVMSASGGKISLFNMMTFKTMTTFMPPPPAATFLAFHPQDN

:* .**:**.**************************************************

D.fullonum_TPL NIIAIGMDDSTIQIYNVRVDEVKSKLKGHSKRITGLAFSHVLNVLVSSGADAQLCVWSSD

S.indicum_TPL NIIAIGMDDSTIQIYNVRVDEVKSKLKGHSKRITGLAFSHVLNVLVSSGADAQLCVWSSD

A.anserina_TPL NIIAIGMDDSTIQIYNVRVDEVKSKLKGHSKRITGLAFSHVLNVLVSSGADSQVCVWNSD

D.carotasativus_TPL NIIAIGMDDSTIQIYNVRVDEVKSKLKGHSKRITGLAFSHVLSVLVSSGADAQLCVWSSD

C.sinensis_TPL NIIAIGMDDSTIQIYNVRVDEVKSKLKGHSKRITGLAFSHVLNVLVSSGADAQLCVWSSD

******************************************.********:*:***.**

D.fullonum_TPL GWEKQRARYLQVPAGRTPTAQSDTRVQFHQDQIHFLVVHETQLAIYETTKLDSVKQWVPR

S.indicum_TPL GWEKQKSRFLQLPSGRSPAAQSETRVQFHQDQIHFLVVHETQLAIYETTKLECVKQWVPR

A.anserina_TPL GWEKQKSRFLQLPAGRTPSSQSDTRVQFHQDQTHFLVVHETQLAIFETTKLECVKQWVPR

D.carotasativus_TPL GWEMQKSRYLQVPAGRTATAQSDTRVQFHQDQLHFLVVHESQLAIYETTKLECVKQWVPR

C.sinensis_TPL GWEKQKNRFLQMPSGRTTTAQSDTRVQFHQDQTHFLVVHETQLAIYEATKLDCVKQWVPR

*** *: *:**:*:**:.::**:********* *******:****:*:***:.*******

D.fullonum_TPL ESAAPISHATFSCDSQLVYASFLDATLCVFSASHLRLRCRINPLAYLSPNVSNSNVHPLV

S.indicum_TPL ESAAPISHATFSCDSQLVYASFLDATVCIFTAAHLRLRCRINPSAYLSPGVS-SNVHPLV

A.anserina_TPL DSAAPISHATFSCDSQLIYASFLDATVCVFSAANLRLRCRINPPAYLPANVSSSNVQPLV

D.carotasativus_TPL ESAAPISHATFSCDSQLVYASFLDASLCIFTATHLRLRCRISPLAYLSPNVSNANIHPLV

C.sinensis_TPL EAAAPISHATFSCDSQLVYASFFDATVCVFTAANLRLRCRINPPSYLSPSVSNSNVHPLV

::***************:****:**::*:*:*::*******.* :**...** :*::***

D.fullonum_TPL VAAHPQEPNQFALGLSDGGVYVFEPLESDGKWGVPPPVENGAASSVPTTAPSVGA-SGSD

S.indicum_TPL IAAHPQEPNQFALGLSDGSVHVFEPLESEGKWGVPPPAENGSTSSVPTT-PLVGA-SASD

A.anserina_TPL IAAHPQEPNQFALGLSDGAVHVFEPLESEGKWGVPPPVENGSTSSVPAT--QVGN-SSSE

D.carotasativus_TPL IAAHPQEPNQFALGLSDGGVHVFEPLESEGKWGVPPPAENGSATSGTNT-PSVGA-SGPD

C.sinensis_TPL IAAHPQEPNQFALGLSDGGVHVFEPLESEGKWGVPPPVENGSASSVPAT-PSVGGPSGSD

:*****************.*:*******:********.***:::* . * ** *..:

D.fullonum_TPL QPQR

S.indicum_TPL QAQR

A.anserina_TPL QAQR

D.carotasativus_TPL QPQR

C.sinensis_TPL QPQR

*.**


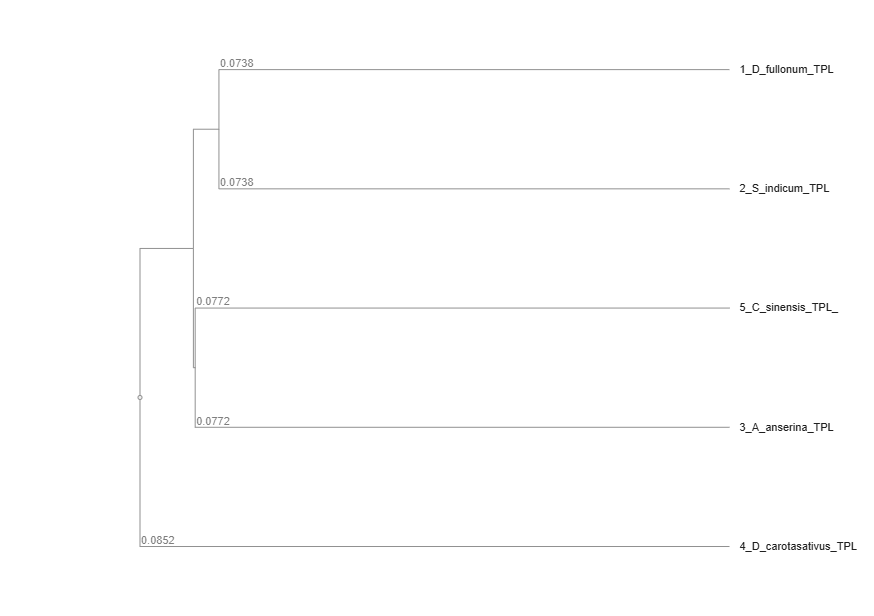


# DfXTH9

Domain: cd02176

D.fullonum_XTH9 ------------------------------------------------------------

D.stramonium_XTH9 ------------MA----SFS------------------------------------WVF

T.subulata_XTH9 ------------MAIALPKMS------------------------------------VLL

N.attenuata_XTH9 ------------MVSFPMEFK------------------------------------WVF

S.alatum_XTH9 MTRHTGFPLKPTLINAAPYINPSSSPPLLQPPPLPPLYPPLRIQKKKAAKMVKGYAIWVV

D.fullonum_XTH9 ------------------------------------------------------MFGKVT

D.stramonium_XTH9 FGFSLMMVGLVSSSRFEELYQPSWALDHLTTEGEILRMKLDHLSGTGFQSKSKYMFGKVT

T.subulata_XTH9 CFCVLVMVGLVSSARFDELFQPSWALDHFIYEGELLKLKLDNYSGSGFQSKSKYMFGKVT

N.attenuata_XTH9 LGISLMLVGLVSSSRFEELYQPSWATDHLTNEGEILRMKLDNLSGAGFSSKNKYMFGKVT

S.alatum_XTH9 IFAALAVVACVSSSKFDELFQPSWAVDHFTYEGEVLRMKLDNYSGAGFSSKSKYMFGKVT

******

D.fullonum_XTH9 IQIKLVEGDSAGTVTAFYMSSEGPKHHEFDFEFLGNTSGEPYLVQTNVYVNGVGNREQRL

D.stramonium_XTH9 VQIKLVEGDSAGTVTAFYMSSEGPTHNEFDFEFLGNTSGEPYTVQTNVYVNGVGNREQRL

T.subulata_XTH9 VQIKLVEGDSAGTVTAFYMSSDGPYHNEFDFEFLGNTTGEPYLVQTNVYVNGVGNREQRL

N.attenuata_XTH9 VQIKLVEGDSAGTVTAFYMSSEGPTHNEFDFEFLGNTTGEPYSVQTNVYVNGVGNREQRL

S.alatum_XTH9 IQIKLVEGDSAGTVTAFYMSSDGPYHNEFDFEFLGNTTGEPYLVQTNVYVNGVGNREQRL

:********************:** *:**********:**** *****************

D.fullonum_XTH9 NLWFDPTKDFHSYSILWNQRQVVFLVDETPIRVHLNMEHKGVPYPKDQPMGVYSSIWNAD

D.stramonium_XTH9 KLWFDPSKDFHSYSILWNQRQVVFLVDETPVRVHSNLEHRGIPFPKDQPMGVYSSIWNAD

T.subulata_XTH9 NLWFDPTKDFHSYSILWNQHQVVFLVDETPIRVHTNMESKGVPFPKDQAMGVYSSIWNAD

N.attenuata_XTH9 NLWFDPSKEFHSYSILWNQRQVVFLVDDTPVRVHSNLEHKGIPFPKDQPMGVYSSIWNAD

S.alatum_XTH9 NLWFDPTKDFHSYSILWNQRQVVFLVDETPVRVHSNLEHKGVPYPKDQAMGVYSSIWNAD

:*****:*:**********:*******:**:*** *:* :*:*:****.***********

D.fullonum_XTH9 DWATQGGRVKTDWTHAPFIASYQGFEIDGCECSVTVVDTNNARRCSSS--EKRFWWDEPT

D.stramonium_XTH9 DWATQGGRVKTDWSHAPFVASYRGFEISGCECLATVAATENARRCSSS-GEKRYWWDEPV

T.subulata_XTH9 DWATQGGRVKTDWSHAPFIASYKGFEIDGCECPVSVAAADNAKKCSSSNGEKRYWWDEPT

N.attenuata_XTH9 DWATQGGRVKTDWSHAPFIASYRGFEIDGCECPATVAAAENSKRCSSS-TEKRYWWDEPT

S.alatum_XTH9 DWATQGGRVHTDWTHAPFVASYKGFEIDGCECPDAVAASDNMRRCSSG-GERRYWWDEPT

*********:***:****:***:****.**** :*. ::* ::***. *:*:*****.

D.fullonum_XTH9 LSKLSYHQSHQLLWVRANQMVYDYCVDTVRFPVAPVECKHHHH-----

D.stramonium_XTH9 MSELSVHQSHQLIWVRANHMVYDYCTDTARFPVAPVECQHHQHKTTHN

T.subulata_XTH9 LSELSLHQSHQLLWVKANHMVYDYCSDTARFPVTPVECQHHHH-----

N.attenuata_XTH9 MSELSLHQSHQLIWVRANHMVYDYCTDTARFPVAPVECQHHQHKTRN-

S.alatum_XTH9 MSELSLHQSHQLLWVRANHMVYDYCYDAARFPVRPVECEHHRH-----

:*:** ******:**:**:****** *:.**** ****:**:*


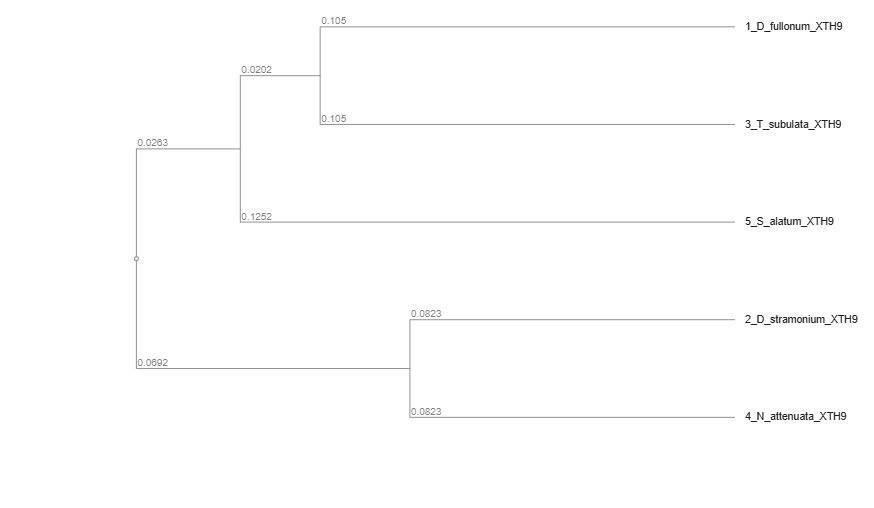

Supplement: Supplementary file 8 — File S1: Alignments of amino acid sequence of the genes analyzed. [file PPL-177-e70642-s003.docx]
